# Discovery of a Potent and Selective JNK3 Inhibitor with Neuroprotective Effect Against Amyloid β-Induced Neurotoxicity in Primary Rat Neurons

**DOI:** 10.3390/ijms222011084

**Published:** 2021-10-14

**Authors:** Joonhong Jun, Jihyun Baek, Songyi Yang, Hyungwoo Moon, Hyejin Kim, Hyunwook Cho, Jung-Mi Hah

**Affiliations:** 1 Department of Pharmacy, College of Pharmacy, Hanyang University, Ansan 15588, Korea; jjh0328@hanyang.ac.kr (J.J.); jhb3534@hanyang.ac.kr (J.B.); songi0822@hanyang.ac.kr (S.Y.); mhu91@hanyang.ac.kr (H.M.); gpwls6121@hanyang.ac.kr (H.K.); lod0201@hanyang.ac.kr (H.C.); 2 Center for Proteinopathy, Institute of Pharmaceutical Science and Technology, Hanyang University, Ansan 15588, Korea

**Keywords:** JNK3, benzimidazole, neurodegenerative diseases, SAR, neuroprotection, Alzheimer’s disease (AD)

## Abstract

As members of the MAPK family, c-Jun-N-terminal kinases (JNKs) regulate the biological processes of apoptosis. In particular, the isoform JNK3 is expressed explicitly in the brain at high levels and is involved in the pathogenesis of neurodegenerative diseases such as Alzheimer’s disease (AD) and Parkinson’s disease (PD). In this study, we prepared a series of five 6-dihydroxy-1*H*-benzo[d]imidazoles as JNK3 inhibitors and found them have potential as neuroprotective agents. Following a previous lead scaffold, benzimidazole moiety was modified with various aryl groups and hydroxylation, and the resulting compounds exhibited JNK3 inhibitory activity with improved potency and selectivity. Out of 37 analogues synthesized, (*S*)-cyclopropyl(3-((4-(2-(2,3-dihydrobenzo[b][1,4]dioxin -6-yl)-5,6-dihydroxy-1H-benzo[d]imidazol-1-yl)pyrimidin-2-yl)amino) piperidin-1-yl)methanone (**35b**) demonstrated the highest JNK3 inhibition (IC_50_ = 9.7 nM), as well as neuroprotective effects against Aβ-induced neuronal cell death. As a protein kinase inhibitor, it also showed excellent selectivity over other protein kinases including isoforms JNK1 (>1000 fold) and JNK2 (−10 fold).

## 1. Introduction

c-Jun N-terminal kinase is a member of a large group of serine/threonine-inducing kinases known as mitogen-activated protein kinases (MAPK) [1]. Various stress factors such as oxidative stress, cytokines, and ultraviolet rays can activate the JNK signaling pathway, inducing the apoptotic pathway of cells [2,3,4,5]. Activated JNK promotes the phosphorylation of a variety of transcription factors, most notably the c-Jun component of AP-1 and cellular proteins, particularly those associated with apoptosis (e.g., Bcl2, p53). In addition, JNK genes form different types of isoforms by splicing, and there are three human JNK genes, jnk1, jnk2, and jnk3, which encode ten diverse splice JNK variants (four JNK1/2 isoforms and two JNK3 isoforms) [1]. While JNK1/2 are widely expressed, JNK3 is expressed explicitly in the brain at high levels and in the heart and testes at low levels. Various studies have been conducted on the relationship between JNK3 and neurodegenerative diseases such as Alzheimer’s disease (AD).

In particular, it has been reported that JNK3 phosphorylates and activates amyloid precursor protein (APP), and the phosphorylation of APP results in its location in cell membranes to promote its conversion to amyloid β, resulting in apoptosis of neuron cells. Additionally, the toxicity of oligomeric amyloid β is known to be mainly propagated by reactivation of JNK3 [6]. More convincingly, a dramatic decrease in oligomeric amyloid β and an increase in cognitive ability caused by the removal of jnk3 are observed in mice with familial Alzheimer’s disease (FAD). Furthermore, not only in amyloid β pathology, JNK3 also directly phosphorylates Tau protein, facilitating the formation of neurofibrillary tangles, which is positively correlated with cognitive impairment and neuronal loss [7].

**Figure 1:**
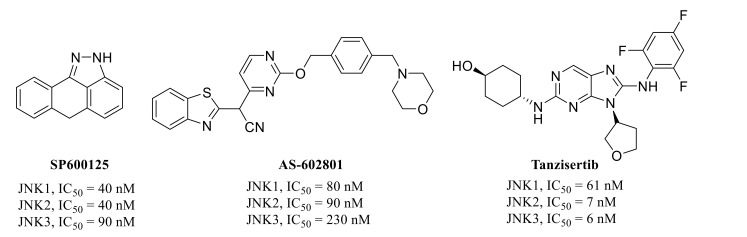
Chemical structures of pan-JNK inhibitors investigated in clinical trial.

Three pan-JNK inhibitors, SP600125, AS-602801, and Tanzisertib have been introduced and suggested to target JNK3 in neurodegenerative disease (Figure 1). SP600125 was the first reported potent pan-JNK inhibitor with poor selectivity over other MAPKs, such as p38 and Erk. Studies have showed that SP600125 leads to decreased formation of neurofibrillary tangles and oligomeric amyloid β plaques and improves AD-associated cognitive declines in APPswe/PS 1dE9 double transgenic mice [8]. AS602801 was another pan-JNK inhibitor identified in the process of drug development, but for other diseases. In 2012, this compound reached phase 2 clinical trial studies to evaluate its ability to treat inflammatory endometriosis [9]. Tanzisertib, another potent pan-JNK inhibitor, was investigated for treatment of discoid lupus erythematosus and IPF in clinical trials in 2011, which were terminated at the phase 2 clinical trial stage [10].

Even though it has shown its potential as a therapeutic target for AD through many studies, the failure of pan-JNK inhibitors in clinical trials has brought our attention to the development of highly selective JNK3 inhibitors for AD therapeutics [11]. However, all three JNK isoforms have an ATP binding pocket with a highly conserved sequence; thus, few compounds exhibiting high selectivity for JNK3 have been discovered. Due to side effects in response to these selectivity issues, there is increasing interest in finding a JNK3 selective inhibitor.

**Figure 2:**
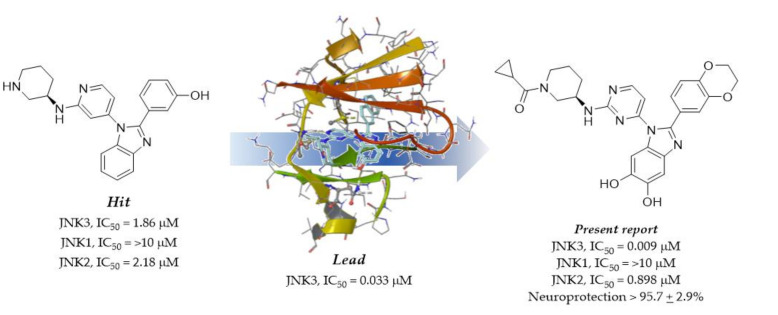
Discovery of JNK3 selective inhibitors from previous lead based on co-crystal structure.

Previously, we have found 1-pyridyl-2-aryl-1*H*-benzimidazole as a hit from our library and its derivatives that display selectivity and activity for JNK3 through optimization [12]. Based on its co-crystal structures, we continued our efforts to develop new JNK3 inhibitors with better potency and isoform selectivity. During the further optimization, we sought to maintain three interactions of the previous scaffold-JNK3 complex, two hydrogen bonds in the hinge region with Met149, a hydrophobic interaction of the large aromatic ring with residues such as Met148, Val79, Val145, Leu144, Ala91, Ile92, Ile124, and Leu128, and the hydrogen bond of a hydroxyl group in benzimidazole with Asn152. Therefore, we investigated more diverse larger aromatic rings for hydrophobic interaction and additional hydrogen bond moieties on the benzimidazole ring for further SAR. Finally, we obtained 1-(2-aminopyrimidin-4-yl)-2-aryl-1*H*-benzo[d]imidazole-5, 6-diol derivatives as potent and selective JNK3 inhibitors (Figure 2).

## 2. Results and Discussion

**Scheme 1:**
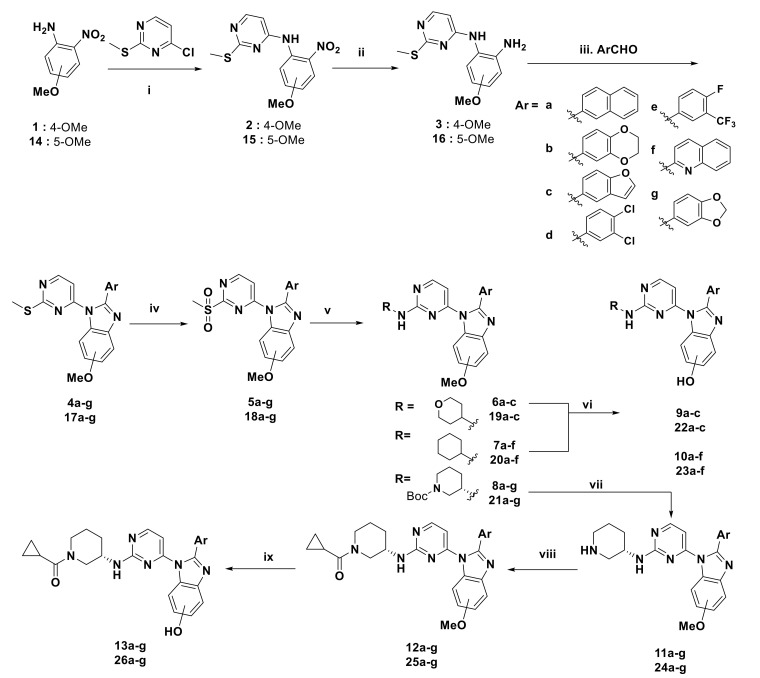
Reagents and reaction condition (**i**) 60% NaH, DMF, 0 °C to rt, 2 h; (**ii**) Pd/C, H_2_, MeOH, rt, 6 h; (**iii**) ArCHO, Na_2_S_2_O_5_, DMF, mw (120 °C, 150 W, 30 min); (**iv**) oxone, MeOH:H_2_O = 1:1, rt, 1 h; (**v**) NH_2_R, THF, 60°C, 5 h; (**vi**) BBr_3_, DCM, −78 °C to rt, 2 h; (**vii**) 4N-HCl in 1,4-dioxane, rt, 20 min; (**viii**) cyclopropanecarbonyl chloride, TEA, THF, 0 °C to rt, 1 h; (**ix**) BBr_3_, DCM, −78 °C to rt.

The synthetic process to 1-(2-(alkylamino)pyrimidin-4-yl)-2-aryl-1H-benzo[d]imidazol-5- or 6-ol derivatives is shown in Scheme 1. Commercially available starting materials 4- or 5-methoxy-2-nitroaniline (**1**, **14**) were treated first with sodium hydride and then reacted with 4-chloro-2-methylthiopyrimidine to produce **2**, **15** [13]. The nitro group was reduced to amine by hydrogenation [14] for benzimidazole formation (**4**, **17**). Various aryl moieties were introduced at the 2-position of the benzimidazole through oxidative cyclization with diaminobenzene and aryl aldehydes (**a**-**g**). After formation of the core benzimidazole scaffold, oxidation of the methyl sulphide in the pyrimidyl ring to methyl sulfone (**5**) was accomplished using mCPBA [15], and several kinds of amino groups were introduced to the pyrimidyl moiety (S_N_Ar) [16] to supply 2-aryl-1H-benzo[d]imidazol-1-yl)pyrimidin-2-yl)amino analogues. When the Boc-protected amino-piperidine group was substituted, deprotection and subsequent acylation were carried out to form products **12** and **25**. Finally, demethylation [17] was performed using BBr_3_ to obtain the final target compounds **13a-p**, **26a-p**.

**Scheme 2:**
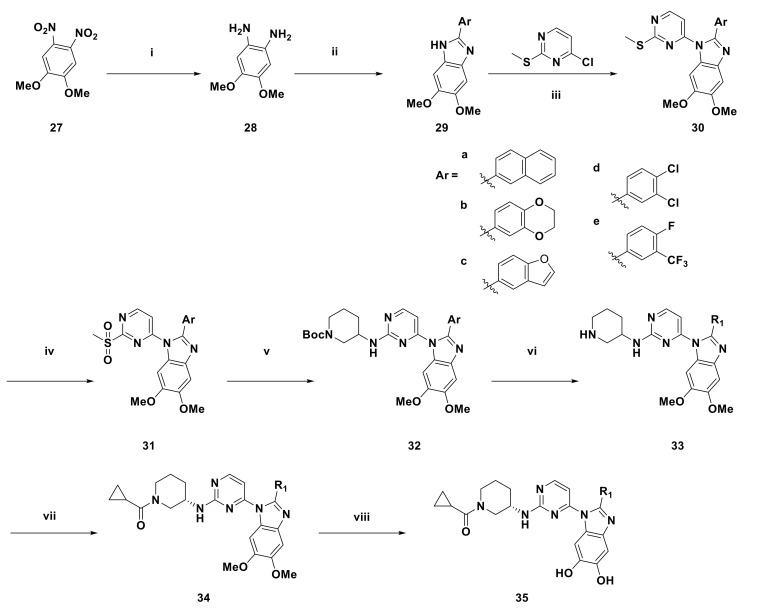
Reagents and reaction condition (**i**) Pd/C, H_2_, MeOH, rt, 6 h (**ii**) ArCHO, Na_2_S_2_O_5_, DMF, MW (120 °C, 150 W, 1.5 hr); (**iii**) 4-Cl-2-(methylthio)pyrimidine, Pd(OAc)_2_, X-Phos, Cs_2_CO_3_, toluene (**iv**) oxone, MeOH:H_2_O = 1:1, rt, 1 h; (**v**) NH_2_R, THF, 60 °C, 5 h; (**vi**) 4N-HCl in 1,4-dioxane, rt, 20 min; (**viii**) cyclopropanecarbonyl chloride, TEA, THF, 0 °C to rt, 1 h; (**ix**) BBr_3_, DCM, −78 °C to rt.

In case of 5, 6-dihydroxy-2-aryl-1H-benzo[d]imidazol-1-yl)pyrimidin-2-yl)amino) piperidin-1-yl derivatives, oxidative cyclization from 1,2-dimethoxy-4,5-dinitrobenzene (**28**) with aryl aldehydes was accomplished to produce the benzimidazole core, as shown in Scheme 2. Then, 2-aryl benzimidazoles (**29**) were coupled with 4-chloro-2-(methylthio)pyrimidine using modified Buchwald conditions, and the remaining steps proceeded the same as for Scheme 1 to obtain five additional final products (**35a-e**).

All of the monohydroxy-benzimidazole compounds, **9a-9c**, **10a-10e**, **13a-13g**, **22a-22c**, **23a-23e**, **26a-26g**, were evaluated for their inhibitory activity against JNK3, and the data are shown in Table 1. Overall, for aromatic substitutions at position 2 of the benzimidazole, the 6-hydroxybenzimidazole (**22a-22c, 23a-23e, 26a-26g**) series was more potent than the 5-hydroxybenzimidazole analogues (**9a-9c**, **10a-10e**, **13a-13g**). We introduced the hydroxyl group at the 5- or 6-position for hydrogen bonding with Asn152 or Ser193, respectively, based on speculation of the co-crystal structure of a previous lead with JNK3. It seems that other interactions of the inhibitors exert an effect that pulls the inhibitors toward the hinge region with preference of the hydrogen bond between the 6-hydroxyl group and Asn152.

Second, we investigated the change in activity with various aromatic substituents (Ar). Larger aryl groups, such as naphthyl and dichlorophenyl, at position 2 of the benzimidazole elicited more potent activity toward JNK3 (**a**, **b**, **d** vs. **e**, **f**, **g**). This effect seems to be related to the electron density and planarity of the aromatic ring due to a Met115 sulphur-π interaction in the active site of JNK3. Compared with mono-substituted phenyl groups, the π-rich dichlorophenyl and naphthyl groups could form a stronger π-π interaction, which might affect the activity as well as selectivity. Nevertheless, even in the case of aromatic groups with hydrophilic substituents (**f**, **g**), the maintained activity implies possible polar interactions such as hydrogen bonding.

**Figure 3:**
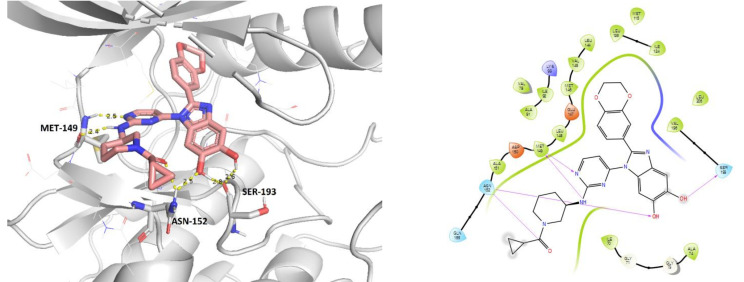
Molecular docking model of compound 35b bound with JNK3.

Next, to investigate the effect of the R substituent at the solvent exposure part of the structure, cyclohexyl, pyranyl, and (S)-cyclopropyl(3-methylpiperidin-1-yl)methanone moieties were introduced, and the inhibitory activity was best for (S)-cyclopropyl(3-methylpiperidin-1-yl)methanone throughout the analogues. This result also suggested that the configuration of the amino group in the ring is important for binding, even in the solvent exposure part, for optimal extra-hydrogen bonding. This extra hydrogen bonding seemed more plausible for the (S)-piperidine in docked structures (Figure 3).

From all these investigations, we decided to synthesize five 6-dihydroxy benzimidazoles with the same solvent exposure group R (**35a**-**35e**). As expected, all dihydroxy analogues with five kinds of aromatic ring substituents showed much higher inhibitory activity than the corresponding mono-hydroxy analogue, implying two hydrogen bonds. Moreover, the five compounds were all very selective JNK3 inhibitors when their inhibitory activities toward JNK1 and JNK2 were compared, and the best compound was (S)-cyclopropyl(3-((4-(2-(2,3-dihydrobenzo[b][1,4]dioxin-6-yl)-5,6-dihydroxy-1H-benzo[d]imidazol-1-yl)pyrimidin-2-yl)amino)piperidin-1-yl)methanone (**35b**), with an IC_50_ value of 9.7 nM for JNK3 and excellent selectivity over JNK2 and JNK1(Table 2).

A docking study was conducted to understand the binding mode of the novel JNK3 inhibitors (Figure 3). When we performed the docking experiment of **35b** with a known JNK3 structure (4KKH), we observed many interactions that could contribute to complex stabilization. First, the aminopyrimidine used as the hinge binder formed two hydrogen bonds with Met149 of JNK3, and two additional hydrogen bonds were plausible between the hydroxyl oxygens of **35b** with Asn152 or Ser193. Moreover, the cyclopropylcarboxamide group in **35b** was in close proximity to Gln155 in the extended hinge region. Lastly, the benzdioxyl ring at position 2 of the benzimidazole fits into the hydrophobic pocket formed by residues Met148, Val79, Val145, Leu144, Ala91, Ile92, Ile124, and Leu128.

**Figure 4:**
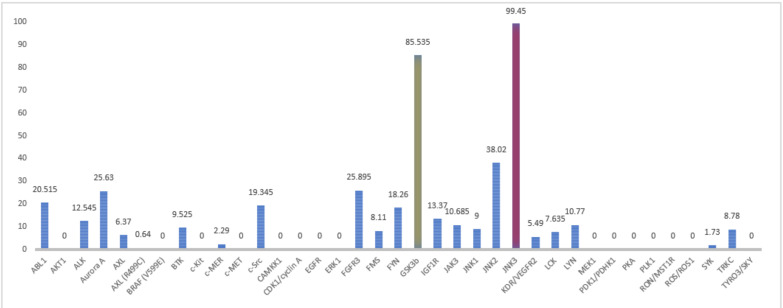
Percentages of enzymatic inhibition exerted by 1 μM of **35b** on 38 selected protein kinases^24^.

Next, we performed kinase panel screening in duplicate for compound **35b** with over 38 kinases at a single-dose concentration of 1 μM (Table 3 Figure 4). This compound had an inhibitory activity of 90% on JNK3 at a concentration of 1 μM; the inhibition activity was less than 15% for most other kinases, an excellent selectivity profile with only slight activities on JNK2 and GSK3β. On further determination of the IC_50_ of **35b** on GSK3β in comparison with JNK3, the selectivity was more than 600-fold higher (Table 3). Since GSK3β is reported to be associated with neurodegenerative disease caused by neuronal apoptosis [19,20], we can manipulate these characters of **35b** for further developments.

To establish whether the derived selective JNK3 inhibitor actually has the ability to protect neurons from amyloid β-induced neuronal cell death, which is known as the pathogenesis of Alzheimer’s disease, we performed cytotoxicity experiments using Aβ 42, which is known as the most toxic fragment of the amyloid protein. The amyloid-β is known to mainly cause apoptosis during cell death, so it was confirmed whether the derived selective JNK3 inhibitors affect the activation (cleavage) of caspase-3, which are apoptosis-related signaling substances. The signaling activation of caspase-3 was confirmed by Western blot and confirmed that the cleaved form, an activated form of caspase-3 was increased when amyloid-β was treated in neurons, and JNK3 inhibitors inhibited the activation of caspase-3 and PARP by amyloid-β treatment (Supplementary Figure S1). This means that selective JNK3 inhibitors can inhibit apoptosis signaling by amyloid-β in neurons, thereby inhibiting apoptosis. Additionally, we confirmed that JNK activation was induced by amyloid-β in neurons, and the derived selective JNK3 inhibitors could inhibit JNK activation induced by amyloid-β. It was confirmed that they inhibit phosphorylation of c-jun induced by amyloid-β in neurons (Supplementary Figure S1). Then, the experiments for JNK3 inhibitors’ effect on the viability of neurons were conducted for five 6-dihydroxy benzimidazoles (**35a**-**35e**) by comparing it with previously published resveratrol as a positive control. On the 5th day of rat primary cortical neuron differentiation, each compound was pre-treated for 90 min and then treated with 10 μM Aβ 42 (HIFP-treated) for 24 or 48 h, and cell viability was measured by MTT assay. All 5, 6-dihydroxy benzimidazoles (**35a**-**35e**) showed neuroprotective effects against Aβ 42 treated neurons in a concentration-dependent manner (Table 4). The neuroprotective activity of **35b** was significantly superior to that of the pan-JNK inhibitor, SP600125, and little less than known resveratrol.

## 3. Materials and Methods

### 3.1. Molecular Modelling

Compounds were docked into the JNK3 structure (PDB: 4KKH). Protein and ligand preparations were performed with Schrödinger’s tools using standard settings, and Glide was used for docking and scoring. 3D X-ray protein structures of JNK3 as a complex with ligands were obtained from the PDB (code: 4KKH) and were prepared using the Protein Preparation Wizard of the Schrödinger Maestro program. All water molecules were removed from the structure, and it was selected as a template. The structures of inhibitors were drawn using Chemdraw, and their 3D conformations were generated using the Schrödinger LigPrep program with the OPLS 2005 force field. Molecular docking of compounds into the structure of JNK3 (PDB code: 4KKH) was carried out using Schrodinger Glide (Version 12.7).

### 3.2. Evaluation of IC_50_ on JNK3 and Selected Kinase Profiling

We used Reaction Biology Corp.’s Kinase HotSpot^SM^ service (Reaction Biology Corp. Malvern, PA) for IC_50_ determination of all compounds and kinase profiles. Assay protocol: in a final reaction volume of 25 μL, substrate ATF2 5 μM, ATP 10 μM, and JNK3(h) (5–10 mU) were incubated with 25 mM Tris (pH 7.5), 0.02 mM EGTA, 0.66 mg/mL myelin basic protein, 10 mM Mg acetate, and [γ-33P-ATP] (specific activity approx. 500 cpm/pmol, concentration as required). The reaction was initiated by the addition of the Mg-ATP mix. After incubation for 40 min at room temperature, the reaction was stopped by the addition of 5 μL of a 3% phosphoric acid solution. Then, 10 μL of the reaction were spotted onto a P30 filtermat and washed three times for 5 min in 75 mM phosphoric acid and once in methanol prior to drying and scintillation counting. Base reaction buffer: 20 mM Hepes (pH 7.5), 10 mM MgCl_2_, 1 mM EGTA, 0.01% Brij35, 0.02 mg/mL BSA, 0.1 mM Na_3_VO_4_, 2 mM DTT, 1% DMSO, Required cofactors are added individually to each kinase reaction. Procedure step-by-step: ① Prepare substrate in freshly prepared base reaction buffer. ② Deliver any required cofactors to the substrate solution above. ③ Deliver indicated kinase into the substrate solution and gently mix. ④ Deliver compounds in 100% DMSO into the kinase reaction mixture by Acoustic technology (Echo550; nanoliter range); incubate for 20 min at room temperature. ⑤ Deliver 33P-ATP into the reaction mixture to initiate the reaction. ⑥ Incubate kinase reaction for 2 h at room temperature. ⑦ Detect kinase activity by P81 filter-binding method.

### 3.3. Cell Viabolity Assays

In initial experiments, rat hippocampal cells grown in serum-free neurobasal media containing B27 supplements at day 6 were treated with different concentrations of Aβ_1–42_ or Aβ_1–40_ for 24 h and then the cell viability was measured by colorimetric MTT assay. Aggregated Aβ_1–42_ and Aβ_1–40_ caused up to 40–60% cell death at concentrations ranging from 5 to 20 mM. In this study, resveratrol, an active component from grapes, was shown to concentration-dependently protect against Aβ-induced toxicity in cultured hippocampal neurons. Resveratrol was active against various amyloid-related peptides including Aβ_1–42_, the most neurotoxic amyloid derivative present in the AD brain. Interestingly, resveratrol was able to block Aβ-induced toxicity not only following a pre- or co-treatment with the toxic peptide, but even to rescue neurons post-Aβ exposure. Primary Rat Cortex Neurons, Sprague Dawley (Gibco, A36512, Fisher Scientific, Göteborg, Sweden), were cultured in Neurobasal™ Plus culture medium (Gibco, A3582901 Fisher Scientific, Göteborg, Sweden), supplemented with B-27™ Supplement (Gibco, A3582801 Fisher Scientific, Göteborg, Sweden) and 0.5 mM GlutaMAX™ Supplement (Gibco, 35050061 Fisher Scientific, Göteborg, Sweden) at 37 °C in a humidified 5% CO_2_ atmosphere. We plated –2 × 105 live cells per well in a poly-D-lysine/laminin coated 24-well plate. For neural differentiation, half of the medium was replaced with fresh complete medium every third day. On day 6, we removed half the volume of media from the culture plate, added an equal amount of complete culture media containing test compounds or vehicle to each well, and incubated them for 90 min at 37 °C and 5% CO2. Immediately prior to use, amyloid β-Protein (1–42) (HFIP-treated) (Bachem, 4090148.0100) was dissolved in 1% NH4OH, further diluted with culture medium, and added into the plates to a final concentration of 10 μM. Cells were incubated for 24 h with vehicle control or test compounds in the presence or absence of Aβ42. Cell viability was measured using the MTT [3-(4, 5-dimethylthiazol-2-yl)-2,5-diphenyltetrazolium bromide] assay. MTT solution was added into each well at a final concentration of 0.5 mg/mL, and cells were incubated at 37 °C for 4 h. The absorbance was detected at 540 nm (reference 650 nm) with a microplate reader. All results were normalized to OD values measured from the vehicle control (DMSO).

### 3.4. Chemistry

All chemicals were of reagent grade and were purchased from Aldrich (USA), TCI (Rep of Korea), Alfa Aesar, Acros. Purification of the compounds by column chromatography was carried out with silica gel 60 (200–300 mesh ASTM, E. Merck, Germany). The quantity of silica gel used was 50–100 times the weight charged on the column. Thin layer chromatography (TLC) was run on the silica gel-coated aluminum sheets (silica gel 60 GF254, E. Merck, Germany) and visualized under ultraviolet (UV) light (254 nm). ^1^H NMR and ^13^C NMR spectra were recorded on a Brucker model digital AVANCE III 400 MHz spectrometer at 25 °C using tetramethylsilane (TMS) as an internal standard. High-resolution MS (HR/MS) experiments were conducted with a Q-TOF/Mass spectrometer 6530 (Agilent Technologies, Santa Clara, CA, USA) operated in positive-ion electrospray mode.

#### 3.4.1. Syntheses of 2-Aryl-1-(2-((tetrahydro-2H-pyran-4-yl)amino)pyrimidin-4-yl)-1H-benzo[d]imidazol-5-ol (9a-9c, 22a-22c)

After dissolving compound 6a (24 mg, 0.053 mmol) in methylene chloride (0.5 mL), BBr3 (25 μL) was added slowly at −78 °C, and the reaction was stirred for 1 h and then at room temperature for 2 h. After confirming completion of the reaction, MeOH was added to quench the reaction, the organic solvent was removed in vacuo, and the residue was extracted with methylene chloride and washed with saturated NaHCO_3_. The extracted organic layer was dried over anhydrous magnesium sulfate, filtered, concentrated, and purified by preparative chromatography (silica gel, methylene:MeOH = 20:1) to obtain the title compound 9a, 20 mg, yield 86%. ^1^H NMR (400 MHz, DMSO-d_6_) δ 9.34 (1H, s), 8.45 (1H, s), 8.21 (1H, s), 7.92–8.00 (3H, m), 7.37–7.61 (5H, m), 7.13 (1H, d, J = 2.0 Hz), 6.86 (1H, dd, J = 8.8 Hz, J = 2.0 Hz), 4.78 (1H, brs), 3.00–3.03 (1H, m), 2.60–2.66 (1H, m), 1.96 (1H, d, J = 13.6 Hz), 1.75–1.78 (1H, m), 1.45 (1H, s), 1.23–1.34 (2H, m), 1.14 (2H, m); HRMS (ESI^+^) calcd for C_26_H_24_N_5_O_2_ [M+H]+: 438.1925, found 438.4379.

2-(2,3-Dihydrobenzo[b][1,4]dioxin-6-yl)-1-(2-((tetrahydro-2H-pyran-4-yl)amino) pyrimidin-4-yl)-1H-benzo[d]imidazol-5-ol **(9b)** as a white solid, yield 36%; ^1^H NMR (400 MHz, MeOD) δ 8.17 (d, J = 8.9 Hz, 1H), 8.07 (d, J = 7.2 Hz, 1H), 7.21 (d, J = 2.1 Hz, 1H), 7.15–7.08 (m, 2H), 6.99 (d, J = 8.4 Hz, 1H), 6.93 (dd, J = 8.9, 2.4 Hz, 1H), 6.33 (d, J = 7.2 Hz, 1H), 4.35–4.30 (m, 4H), 4.09–4.01 (s, 1H), 3.68 (m, 1H), 2.47–2.34 (m, 2H), 2.28–2.20 (m, 1H), 2.13–1.98 (m, 3H), 1.55 (m, 1H), 1.29 (m, 2H). HRMS (ES^+^) calcd for C_26_H_24_N_5_O_4_ [M+H]+: 446.1823, found 446.3474.

2-(Benzofuran-5-yl)-1-(2-((tetrahydro-2H-pyran-4-yl)amino)pyrimidin-4-yl)-1H-benzo[d] imidazol-5-ol **(9c)** as a white solid, m.p. yield 93%; ^1^H NMR (400 MHz, CD_3_OD) δ 8.27 (d, J = 1.6 Hz, 1H), 7.99 (dd, J = 8.7, 1.6 Hz, 1H), 77.84 (d, J = 4.5 Hz, 1H), 7.83 (d, J = 2.3 Hz, 1H), 7.61 (d, J = 8.7 Hz, 1H), 7.40 (d, J = 8.6 Hz, 1H), 6.98–6.93 (m, 2H), 6.78 (dd, J = 8.6, 2.3 Hz, 1H), 6.29 (d, J = 7.2 Hz, 1H), 4.17–4.04 (m, 2H), 3.96 (s, 2H), 3.84–3.77 (m, 1H), 3.72 (m, 2H), 2.24 (m, 1H), 1.98 –1.84 (m, 2H), 1.80 –1.70 (m, 1H); HRMS (ES^+^) calcd for C_24_H_22_N_5_O_3_ [M+H]+: 428.1717, found 428.3226

2-(Naphthalen-2-yl)-1-(2-((tetrahydro-2H-pyran-4-yl)amino)pyrimidin-4-yl)-1H-benzo[d]imidazol-6-ol **(22a)** %); ^1^H NMR (400 MHz, DMSO- d_6_) δ 9.54 (s, 1H), 8.47 (s, 1H), 8.16 (s, 1H), 7.91–7.97 (m, 3H), 7.39–7.62 (m, 5H), 7.06 (s, 1H), 6.84–6.87 (m, 1H), 3.03–3.07 (m,1H), 2.63–2.67 (m,1H), 1.98 (d, J = 14.0 Hz, 1H), 1.72–1.75 (m, 1H), 1.46 (s,1H), 1.14–1.24 (m, 2H), 1.02–1.10 (m, 2H); HRMS(ESI) calcd for C_26_H_24_N_5_O_2_ [M+H]+: 438.1925, found 438.3749.

2-(2,3-Dihydrobenzo[b][1,4]dioxin-6-yl)-1-(2-((tetrahydro-2H-pyran-4-yl)amino) pyrimidin-4-yl)-1H-benzo[d]imidazol-6-ol **(22b)** 81%; ^1^H NMR (400 MHz, CD_3_OD) δ 8.37 (s, 1H), 7.53 (d, J = 8.4 Hz, 1H), 7.07 (s, 1H), 7.01 (s, 1H), 6.97 (dd, J = 8.4, 2.1 Hz, 1H), 6.90–6.85 (m, 2H), 6.64 (s, 1H), 4.28 (d, J = 5.0 Hz, 2H), 4.26 (d, J = 5.0 Hz, 2H), 3.90 (m, 2H), 3.62 (s, 1H), 3.39 (s, 1H), 1.29 (m, 4H), 0.91 (m, 3H); HRMS (ES^+^) calcd for C_26_H_24_N_5_O_4_ [M+H]+: 446.1823, found 446.2844.

2-(Benzofuran-5-yl)-1-(2-((tetrahydro-2H-pyran-4-yl)amino)pyrimidin-4-yl)-1H-benzo[d]imidazol-6-ol **(22c)** 30%; 1H NMR (400 MHz, CD_3_OD) δ 8.00 (d, J = 7.1 Hz, 1H), 7.97 (d, J = 1.5 Hz, 1H), 7.91 (d, J = 1.5 Hz, 1H), 7.69 (m, 2H), 7.61–7.56 (m, 2H), 6.99–6.95 (m, 2H), 6.20 (d, J = 7.1 Hz, 1H), 4.28 (m, 2H), 3.99 (s, 1H), 3.86 –3.74 (m, 2H), 2.43–2.34 (m, 1H), 2.14–2.02 (m, 2H), 1.92–1.72 (m, 2H), 1.28 (m, 1H). HRMS (ES^+^) calcd for C_24_H_22_N_5_O_3_ [M+H]+: 428.1717, found 428.3226.

#### 3.4.2. Syntheses of 1-(2-(cyclohexylamino)pyrimidin-4-yl)-2-(aryl)-1H-benzo[d]imidazol-5-ol (10a-10f, 23a-23f)

1-(2-(cyclohexylamino)pyrimidin-4-yl)-2-(naphthalen-2-yl)-1H-benzo[d]imidazol-5-ol 10a Compound 7a (37 mg, 0.082 mmol) was dissolved in methylene chloride (0.8 mL), BBr3 (39 µL) was added at −78 ^O^C, and the reaction was stirred for 1 h and then at room temperature for 2 h. After the reaction was complete, MeOH was added to quench the reaction, the organic solvent was removed under reduced pressure, and the residue was extracted with methylene chloride and washed with saturated NaHCO_3_ aqueous solution. The extracted organic layer was dried with anhydrous magnesium sulfate and filtered, the filtrate was concentrated under reduced pressure, and the residue was purified by column chromatography (silica gel, methylene chloride: MeOH = 20: 1) to give target compound **10a** (21 mg, 58%) was obtained. ^1^H NMR (400 MHz, DMSO d_6_) δ 9.35 (s, 1H), 8.40 (s, 1H), 8.21 (s, 1H), 7.95–8.00 (m, 2H), 7.52–7.80 (m, 4H), 7.29 (s, 1H), 7.14 (s, 1H), 6.87 (dd, J = 8.8 Hz, J = 2.4 Hz, 1H), 6.71 (s, 1H), 5.22 (brs, 1H), 2.90 (brs, 1H), 1.15–1.25 (m, 6H), 0.67–0.91 (m, 4H); HRMS(ESI) calcd for C_27_H_26_N_5_O [M+H]+: 436.2132, found 436.1376.

1-(2-(Cyclohexylamino)pyrimidin-4-yl)-2-(2,3-dihydrobenzo[b][1,4]dioxin-6-yl)-1H-benzo [d]imidazol-5-ol **(10b)** 53%; ^1^H NMR (400 MHz, DMSO) δ 9.30 (s, 1H), 8.39 (m, 1H), 7.44 (s, 1H), 7.05 (s, 1H), 6.96 (s, 1H), 6.90 (d, J = 8.5 Hz, 1H), 6.78 (dd, J = 8.8, 2.3 Hz, 1H), 6.60 (s, 1H), 4.25 (s, 4H), 3.22 (s, 1H), 1.92 (m, 1H), 1.57 (m, 3H), 1.25 (m, 3H), 1.07 (m, 4H), 0.86 (m, 1H); ^13^C NMR (101 MHz, DMSO) δ 161.96 (s), 157.02 (s), 154.11 (s), 144.59 (s), 143.76 (s), 143.11 (s), 128.52 (s), 126.56 (s), 123.93 (s), 122.17 (s), 117.49 (d, J = 5.2 Hz), 117.12 (d, J = 4.7 Hz), 112.99 (s), 103.99 (d, J = 19.2 Hz), 64.19 (d, J = 23.7 Hz), 49.43 (s), 32.30 (d, J = 11.9 Hz), 25.30 (d, J = 2.7 Hz), 24.89 (s); HRMS (ESI) calcd for C_25_H_26_N_5_O_3_ [M+H]^+^: 444.2030, found 444.3306.

2-(Benzofuran-5-yl)-1-(2-(cyclohexylamino)pyrimidin-4-yl)-1H-benzo[d]imidazol-5-ol **(10c)** 60%; ^1^H NMR (400 MHz, CD_3_OD) δ 8.25 (s, 1H), 7.83 (s, 2H), 7.55 (d, J = 8.5 Hz, 2H), 7.42 (d, J = 8.3 Hz, 1H), 7.14 (d, J = 2.1 Hz, 1H), 6.96–6.85 (m, 2H), 6.53 (s, 1H), 4.16–3.53 (m, 1H), 3.15 (s, 1H), 1.51 (s, 5H), 1.26 (s, 1H), 1.18–0.82 (m, 5H); ^13^C NMR (101 MHz, MeOD) δ 163.38 (s), 161.36 (s), 159.05 (d, J = 9.8 Hz), 157.02 (s), 155.93 (s), 154.71 (s), 147.90 (s), 144.57 (s), 129.80 (s), 129.29 (s), 126.75 (s), 123.65 (s), 114.72 (s), 113.29 (s), 112.49 (s), 107.94 (s), 105.37 (s), 104.72 (s), 51.05 (s), 34.17–33.23 (m), 26.65 (s), 26.39–25.87 (m). HRMS (ESI) calcd for C_25_H_24_N_5_O_2_ [M+H]+: 426.1925, found 426.3058.

1-(2-(Cyclohexylamino)pyrimidin-4-yl)-2-(3,4-dichlorophenyl)-1H-benzo[d]imidazol-5-ol **(10d)** 54%; H NMR (400 MHz, DMSO-d_6_) d 9.34 (1H, s),8.10 (1H, d, J = 5.6 Hz), 7.82 (1H, d, J = 2.0 Hz), 7.70 (1H, d, J = 8.4 Hz), 7.44 (1H, dd, J = 8.4 Hz, J = 2.4 Hz), 7.19 (1H, d, J = 8.8 Hz), 7.09 (1H, d, J = 2.4 Hz), 6.83 (1H, dd, J = 8.8 Hz, J = 2.4 Hz), 6.71 (1H, d, J = 7.6 Hz), 6.45 (1H, dd, J = 5.6 Hz, J = 1.6 Hz), 6.38 (1H, d, J = 1.6 Hz), 3.61 (2H, s), 1.83–1.85 (2H, m), 1.66–1.70 (2H, m), 1.55–1.59 (1H, m),1.23–1.32 (3H, m), 1.10–1.19 (3H, m); HRMS(ESI) calcd for C_23_H_22_Cl_2_N_5_O [M+H]+: 454.1196, found 454.3513.

1-(2-(Cyclohexylamino)pyrimidin-4-yl)-2-(4-fluoro-3-(trifluoromethyl)phenyl)-1H-benzo [d]imidazol-5-ol **(10e)** 74%;^1^H NMR (400 MHz, CD_3_OD) δ 8.40 (dd, J = 6.6, 2.0 Hz, 1H), 8.31 (m, 1H), 7.85 (m, 1H), 7.62–7.47 (m, 2H), 7.44 (d, J = 8.7 Hz, 1H), 6.97 (d, J = 2.0 Hz, 1H), 6.82 (dd, J = 8.7, 2.3 Hz, 1H), 3.72 (s, 1H), 1.98–1.93 (m, 1H), 1.76 (m, 2H), 1.70–1.49 (m, 4H), 1.18–1.08 (m, 2H), 0.91–0.82 (m, 3H). HRMS (ESI) calcd for C_24_H_22_F_4_N_5_O [M+H]+: 472.1755, found 472.3441.

1-(2-(Cyclohexylamino)pyrimidin-4-yl)-2-(quinolin-2-yl)-1H-benzo[d]imidazol-5-ol **(10f)**, 51%; ^1^H NMR (400 MHz, CD_3_OD) δ 8.44 (t, J = 8.7 Hz, 1H), 8.37 (t, J = 5.5 Hz, 1H), 8.05 (s, 1H), 7.98–7.92 (m, 1H), 7.71 (dd, J = 9.9, 5.1 Hz, 2H), 7.68–7.54 (m, 2H), (d, J = 2.1 Hz, 1H), 7.00–6.93 (m, 1H), 6.74 (s, 1H), 3.35 (s, 1H), 2.85 (s, 1H), 1.57–1.34 (m, 3H), 1.32–1.09 (m, 3H), 1.07–0.72 (m, 5H); 13C NMR (101 MHz, DMSO) δ 161.93 (s), 155.70 (s), 154.35 (s), 146.52 (s), 143.64 (s), 136.79 (s), 136.09 (s), 130.09 (d, J = 6.1 Hz), 128.75 (d, J = 10.5 Hz), 127.92 (s), 127.33 (t, J = 9.9 Hz), 120.81 (s), 114.53 (s), 113.54 (s), 104.36 (s), 96.28 (s), 48.92 (s), 31.80 (d, J = 8.9 Hz), 25.12 (s), 24.61 (d, J = 4.3 Hz); HRMS (ESI) calcd for C_26_H_25_N_6_O [M+H]+: 437.2084, found 437.3665.

1-(2-(Cyclohexylamino)pyrimidin-4-yl)-2-(naphthalen-2-yl)-1H-benzo[d]imidazol-6-ol **(23a)** (10 mg, 38%); ^1^H NMR (400 MHz, DMSO-d_6_) δ 8.42 (s, 1H), 8.15 (s, 1H,), 7.93–7.95 (m, 4H), 7.28–7.62 (m, 5H), 7.07 (s, 1H), 6.85 (dd, J = 8.8 Hz, J = 2.4 Hz, 1H), 6.72 (s,1H), 2.89 (brs,1H), 1.23 (m, 6H), 0.66–0.85 (m, 4H);HRMS (ESI) calcd for C_27_H_26_N_5_O [M+H]+: 436.2132, found 436.3897.

1-(2-(Cyclohexylamino)pyrimidin-4-yl)-2-(2,3-dihydrobenzo[b][1,4]dioxin-6-yl)-1H-benzo [d]imidazol-6-ol **(23b)** 78%); ^1^H NMR (400 MHz, CD_3_OD) δ 8.22 (s, 1H), 7.42 (d, J = 8.6 Hz, 1H), 6.98 (s, 1H), 6.91 (s, 1H), 6.86 (dd, J = 8.4, 2.1 Hz, 1H), 6.77 (dd, J = 8.6, 2.4 Hz, 2H), 6.46 (s, 1H), 4.17 (d, J = 5.1 Hz, 2H), 4.15 (d, J = 5.1 Hz, 2H), 3.34 (s, 1H), 2.20–1.78 (m, 1H), 1.61 (m, 3H), 1.53 (m, 1H), 1.18 (m, 2H), 1.14–0.74 (m, 4H); HRMS(ESI) calcd for C_25_H_26_N_5_O_3_ [M+H]+: 444.2030, found 444.0155.

2-(Benzofuran-5-yl)-1-(2-(cyclohexylamino)pyrimidin-4-yl)-1H-benzo[d]imidazol-6-ol **(23c,** 57%); ^1^H NMR (400 MHz, CD_3_OD) δ 8.30 (d, J = 1.5 Hz, 1H), 7.85 (dd, J = 6.7, 2.2 Hz, 2H), 7.57 (d, J = 8.6 Hz, 2H), 7.43 (d, J = 8.6 Hz, 1H), 7.15 (s, 1H), 6.90 (dd, J = 10.3, 1.5 Hz, 2H), 6.64 (s, 1H), 3.95–3.81 (m, 1H), 3.56 (s, 1H), 2.00 (m, 2H), 1.57 (m, 5H), 0.96–0.81 (m, 4H); ^13^C NMR (101 MHz, DMSO) δ 171.99 (s), 161.92 (s), 160.78 (s), 154.67 (d, J = 19.3 Hz), 146.98 (s), 136.23 (s), 135.69 (s), 127.37 (s), 126.16 (s), 125.28 (s), 121.93 (d, J = 16.7 Hz), 119.97 (s), 112.78 (s), 111.20 (s), 107.07 (s), 103.79 (s), 96.79 (s), 49.18 (s), 32.07 (d, J = 3.7 Hz), 25.15 (s), 24.63 (d, J = 2.1 Hz); HRMS (ESI) calcd for C_25_H_24_N_5_O_2_ [M+H]+: 426.1925, found 426.3058.

1-(2-(Cyclohexylamino)pyrimidin-4-yl)-2-(3,4-dichlorophenyl)-1H-benzo[d]imidazol-6-ol **(23d,** 76%); ^1^H NMR (400 MHz, CD_3_OD) δ 8.32 (s, 1H), 7.64 (s, 1H), 7.49 (dd, J = 8.5, 3.7 Hz, 2H), 7.28 (d, J = 8.5 Hz, 1H), 6.99 (s, 1H), 6.82 (dd, J = 8.7, 2.3 Hz, 1H), 6.68 (s, 1H), 3.13–2.89 (s, 1H), 1.67–1.39 (m, 6H), 1.04 (m, 5H), 0.79 (m, 1H); HRMS (ESI) calcd for C_23_H_22_Cl_2_N_5_O [M+H]^+^: 454.1196, found 454.4773.

1-(2-(Cyclohexylamino)pyrimidin-4-yl)-2-(4-fluoro-3-(trifluoromethyl)phenyl)-1H-benzo [d]imidazol-6-ol **(23e,** 78%); ^1^H NMR (400 MHz, DMSO) δ 9.64 (s, 1H), 8.53–8.45 (m, 1H), 7.83 (d, J = 5.5 Hz, 1H), 7.61 (dd, J = 13.7, 5.5 Hz, 2H), 7.48 (d, J = 7.9 Hz, 1H), 7.00 (s, 1H), 6.86 (dd, J = 9.0, 2.0 Hz, 1H), 2.93 (s, 1H), 1.79 (s, 1H), 1.50 (m, 3H), 1.30 (m, 3H), 0.97 (m, 4H), 0.85 (m, 1H). HRMS (ESI) calcd for C_24_H_22_F_4_N_5_O [M+H]^+^: 472.1755, found 472.3756.

1-(2-(Cyclohexylamino)pyrimidin-4-yl)-2-(quinolin-2-yl)-1H-benzo[d]imidazol-6-ol **(23f**, 78%); ^1^H NMR (400 MHz, DMSO) δ 8.52 (m, 2H), 8.18 (s, 1H), 8.03 (dd, J = 16.3, 7.8 Hz, 1H), 7.76–7.70 (m, 1H), 7.70–7.65 (m, 1H), 7.65–7.59 (m, 1H), 7.56–7.40 (m, 1H), 7.29 (s, 1H), 6.92 (dd, J = 25.0, 9.5 Hz, 2H), 2.87 (s, 1H), 1.99–1.82 (m, 1H), 1.70 (m, 1H), 1.26 (m, 4H), 1.07 (m, 2H), 0.84 (m, 4H); ^13^C NMR (101 MHz, DMSO) δ 161.53 (s), 160.00 (s), 159.39 (s), 155.75 (s), 150.89 (s), 146.57 (s), 137.23 (s), 136.88 (s), 135.92 (s), 130.13 (d, J = 1.6 Hz), 128.68 (s), 127.95 (s), 127.32 (d, J = 7.0 Hz), 125.93 (s), 123.86 (s), 120.76 (s), 113.64 (s), 96.34 (s), 48.61 (s), 31.81 (d, J = 7.6 Hz), 25.13 (s), 24.59 (d, J = 5.7 Hz).HRMS (ESI) calcd for C_26_H_25_N_6_O [M+H]^+^: 437.2084, found 437.2720.

#### 3.4.3. Syntheses of (S)-cyclopropyl(3-((4-(5-hydroxy-2-(naphthalen-2-yl)-1H-benzo[d]imidazol-1-yl)pyrimidin-2-yl)amino)piperidin-1-yl)methanone (13a)

Compound **12a** (66 mg, 0.127 mmol) was dissolved in methylene chloride (1.3 mL), BBr_3_ (60µL) was added at −78 °C, and the reaction was stirred for 1 h and then at room temperature for 2 h. After confirming the completion of the reaction, MeOH was added to quench the reaction, the organic solvent was removed under reduced pressure, and the residue was extracted with methylene chloride and washed with a saturated NaHCO_3_ aqueous solution. The extracted organic layer was dried with anhydrous magnesium sulfate and filtered, the filtrate was concentrated under reduced pressure, and the residue was purified by column chromatography (silica gel, methylene chloride: MeOH = 20: 1), to give the target compound **13a** (39 mg, 61%) was obtained; ^1^H NMR (400 MHz, DMSO-d_6_) δ 9.36 (s, 1H), 8.42–8.18 (m, 2H), 7.96–7.94 (m, 3H), 7.61–7.54 (m, 5H), 7.11 (d, J = 2.4 Hz, 1H), 6.84 (d, J = 7.6 Hz, 1H), 6.67–6.25 (m, 1H), 4.78 (s, 1H), 4.14–3.84 (m, 2H), 3.17–2.85 (m, 2H), 1.97–1.91 (m, 2H), 1.75 (s, 1H), 1.45–1.14 (m, 4H), 0.85–0.69(m, 2H); HRMS m/z calcd for C_15_H_12_Cl_2_N_4_OS 367.2480, found 368.2729 (M+H^+^). HRMS (ESI) calcd for C_30_H_29_N_6_O_2_ [M+H]+: 505.2347, found 505.2722.

(S)-Cyclopropyl(3-((4-(2-(2,3-dihydrobenzo[b][1,4]dioxin-6-yl)-5-hydroxy-1H-benzo[d] imidazol-1-yl)pyrimidin-2-yl)amino)piperidin-1-yl)methanone **(13b,** 52%);^1^H NMR (400 MHz, CD_3_OD) δ 8.33 (s, 1H), 7.63–7.41 (m, 1H), 7.09 (s, 1H), 7.03 (s, 1H), 6.97 (d, J = 8.4 Hz, 1H), 6.92–6.82 (m, 2H), 6.57 (s, 1H), 4.26 (d, J = 6.3 Hz, 4H), 4.07 (s, 1H), 3.49 (s, 1H), 2.97 (m, 1H), 2.02 (m, 2H), 1.79 (m, 2H), 1.61 (m, 4H), 0.95–0.79 (m, 3H), 0.69 (m, 2H). HRMS (ESI) calcd for C_28_H_29_N_6_O_4_ [M+H]+: 513.2245, found 513.0551.

(S)-(3-((4-(2-(Benzofuran-5-yl)-5-hydroxy-1H-benzo[d]imidazol-1-yl)pyrimidin-2-yl) amino)piperidin-1-yl)(cyclopropyl)methanone **(13c,** 57%); ^1^H NMR (400 MHz, DMSO) δ 9.33 (s, 1H), 8.33 (d, J = 47.4 Hz, 1H), 8.07 (s, 1H), 7.87 (d, J = 23.5 Hz, 1H), 7.65 (s, 2H), 7.45 (d, J = 18.7 Hz, 1H), 7.08 (d, J = 2.2 Hz, 1H), 7.02 (s, 1H), 6.82 (d, J = 7.4 Hz, 1H), 4.08 (s, 1H), 2.95 (s, 1H), 1.95 (m, 2H), 1.76 (m, 2H), 1.56 (m, 2H), 1.23 (m, 2H), 0.89–0.66 (m, 4H), 0.63–0.54 (m, 1H), 0.23 (m, 1H); ^13^C NMR (101 MHz, DMSO) δ 176.04 (s), 174.74 (s), 171.96 (s), 165.03 (s), 154.13 (s), 152.23 (s), 147.17 (s), 146.98 (d, J = 4.0 Hz), 146.35 (s), 143.82 (s), 127.34 (d, J = 6.5 Hz), 125.56 (s), 122.32 (s), 113.03 (s), 107.09 (s), 104.00 (s), 100.58 (s), 91.72 (s), 61.51 (s), 53.26 (s), 50.02 (s), 46.33 (s), 29.73 (s), 10.40 (s), 6.90 (d, J = 6.8 Hz); HRMS (ESI) calcd for C_28_H_27_N_6_O_3_ [M+H]+: 495.2139, found 495.6932.

(S)-Cyclopropyl(3-((4-(2-(3,4-dichlorophenyl)-5-hydroxy-1H-benzo[d]imidazol-1-yl) pyrimidin-2-yl)amino)piperidin-1-yl)methanone **(13d,** 41%): ^1^H NMR (400 MHz, MeOD) δ 8.43 (d, J = 18.2 Hz, 1H), 7.76 (s, 1H), 7.59 (m, 2H), 7.40 (d, J = 8.4 Hz, 1H), 7.14 (s, 1H), 6.92 (d, J = 8.7 Hz, 1H), 6.78 (s, 1H), 4.20 (s, 1H), 4.08 (m, 1H), 3.15 (m, 1H), 2.92 (s, 1H), 2.06–1.93 (m, 1H), 1.83 (m, 2H), 1.57 (m, 3H), 1.28 (m, 1H), 0.92–0.77 (m, 3H), 0.65 (m, 1H), 0.36 (m, 1H). HRMS (ESI) calcd for C_26_H_25_Cl_2_N_6_O_2_ [M+H]+: 523.1411, found 523.3586.

(S)-Cyclopropyl(3-((4-(2-(4-fluoro-3-(trifluoromethyl)phenyl)-5-hydroxy-1H-benzo[d] imidazol-1-yl)pyrimidin-2-yl)amino)piperidin-1-yl)methanone **(13e**, 50%); ^1^H NMR (400 MHz, CD_3_OD) δ 8.49–8.37 (m, 1H), 7.92 (s, 1H), 7.80 (s, 1H), 7.64–7.49 (m, 1H), 7.45 (d, J = 9.5 Hz, 1H), 7.14 (s, 1H), 6.92 (d, J = 8.8 Hz, 1H), 6.77 (s, 1H), 4.19 (s, 1H), 2.93–2.72 (m, 1H), 2.05 (m, 1H), 1.91–1.73 (m, 3H), 1.72–1.64 (m, 1H), 1.61–1.50 (m, 2H), 0.94–0.85 (m, 2H), 0.84–0.78 (m, 2H), 0.74 (m, 1H), 0.62 (m, 1H). HRMS (ESI) calcd for C_27_H_25_F_4_N_6_O_2_ [M+H]+: 541.1970, found 541.3818.

(S)-Cyclopropyl(3-((4-(5-hydroxy-2-(quinolin-2-yl)-1H-benzo[d]imidazol-1-yl)pyrimidin-2-yl)amino)piperidin-1-yl)methanone **(13f,** 30%); ^1^H NMR (400 MHz, CD_3_OD) δ 8.49–8.41 (m, 2H), 8.19 (d, J = 8.5 Hz, 1H), 7.95 (d, J = 7.5 Hz, 1H), 7.74–7.67 (m, 2H), 7.61 (m, 2H), 6.95 (dd, J = 8.5, 2.1 Hz, 2H), 6.76 (s, 1H), 4.02 (s, 1H), 3.18 (s, 1H), 2.06–1.93 (m, 1H), 1.60 (m, 3H), 1.39 (m, 4H), 1.29 (m, 4H), 0.97–0.78 (m, 5H), 0.60 (m, 1H); ^13^C NMR (101 MHz, DMSO) δ 155.75 (s), 148.43 (d, J = 4.0 Hz), 146.17 (s), 136.83 (d, J = 7.0 Hz), 135.99 (s), 135.58 (s), 130.21 (d, J = 2.0 Hz), 128.53 (d, J = 2.9 Hz), 127.99 (s), 127.37 (s), 127.31 (s), 120.76 (d, J = 4.9 Hz), 113.63 (d, J = 7.8 Hz), 113.52 (d, J = 6.7 Hz), 49.28 (s), 30.97 (s), 22.08 (s), 13.98 (s), 10.51 (d, J = 2.8 Hz), 6.84 (d, J = 5.7 Hz); HRMS (ESI) calcd for C_29_H_28_N_7_O_2_ [M+H]^+^: 506.2299, found 506.4381.

(S)-(3-((4-(2-(Benzo[d][1,3]dioxol-5-yl)-5-hydroxy-1H-benzo[d]imidazol-1-yl)pyrimidin-2-yl)amino)piperidin-1-yl)(cyclopropyl)methanone **(13g,** 89%); ^1^H NMR (400 MHz, MeOD) δ 8.41–8.25 (m, 1H), 7.55 (m, 1H), 7.08 (s, 1H), 6.94 (s, 1H), 6.90–6.77 (m, 3H), 6.49 (m, 1H), 4.25 (s, 1H), 4.02 (m, 1H), 3.59–3.36 (m, 1H), 3.08 (m, 2H), 2.06–1.86 (m, 2H), 1.77 (s, 1H), 1.61 (m, 3H), 1.29 (m, 1H), 0.90–0.57 (m, 4H), 0.28 (m, 1H). HRMS (ESI) calcd for C_27_H_27_N_6_O_4_ [M+H]^+^: 506.2299, found 506.4381.

(S)-Cyclopropyl(3-((4-(6-hydroxy-2-(naphthalen-2-yl)-1H-benzo[d]imidazol-1-yl) pyrimidin-2-yl)amino)piperidin-1-yl)methanone **(26a,** 93%); ^1^H NMR (400 MHz, DMSO-d_6_) δ 9.52 (s,1H), 8.3 (m, 2H), 7.92–7.95 (m, H), 7.55–7.61 (m, 5H), 6.84 (dd, J = 8.8, 2.0 Hz, 1H), 6.34–6.67 (m, 1H), 3.87–4.39 (m, 3H), 2.80–3.05 (m, 1H), 1.98 (m, 1H), 1.34–1.51 (m, 4H), 0.70–0.85 (m, 4H); ^13^C NMR (100 MHz, DMSO-d_6_) δ 162.2, 160.4, 157.3, 155.0, 136.1, 132.9, 132.5, 128.4 127.8, 127.6, 127.2, 126.8, 125.7, 120.1, 113.2, 113.0, 105.4, 97.5, 49.1, 48.0, 45.1, 29.6, 22.9, 10.5, 6.9 ppm; HRMS (ESI) calcd for C_30_H_29_N_6_O_2_ [M+H]^+^: 505.2347, found 505.0201.

(S)-Cyclopropyl(3-((4-(2-(2,3-dihydrobenzo[b][1,4]dioxin-6-yl)-6-hydroxy-1H-benzo[d] imidazol-1-yl)pyrimidin-2-yl)amino)piperidin-1-yl)methanone (**26b,** 64%); ^1^H NMR (400 MHz, DMSO) δ 9.47 (s, 1H), 8.51–8.28 (m, 1H), 7.80–7.62 (m, 1H), 7.53 (d, J = 8.6 Hz, 1H), 7.01 (m, 1H), 6.90 (m, 2H), 6.79 (dd, J = 8.6, 2.1 Hz, 1H), 4.26 (m, 4H), 4.19–4.07 (m, 1H), 3.86 (s, br, 1H), 3.52 (m, 1H), 3.05 (m, 1H), 2.63 (m, 1H), 1.96 (m, 1H), 1.81 (m, 2H), 1.64–1.35 (m, 2H), 1.21 (m, 1H), 0.83 (m, 3H), 0.58–0.01 (m, 2H). HRMS (ESI) calcd for C_28_H_29_N_6_O_4_ [M+H]^+^: 513.2245, found 513.3702.

(S)-(3-((4-(2-(Benzofuran-5-yl)-6-hydroxy-1H-benzo[d]imidazol-1-yl)pyrimidin-2-yl) amino)piperidin-1-yl)(cyclopropyl)methanone **(26c,** 18%); ^1^H NMR (400 MHz, CD_3_OD) δ 8.35 (s, 1H), 7.84 (m, 2H), 7.56 (dd, J = 8.4, 3.8 Hz, 2H), 7.42 (d, J = 8.1 Hz, 1H), 7.27–7.14 (m, 1H), 7.04 (dd, J = 14.9, 8.4 Hz, 1H), 6.89 (d, J = 8.2 Hz, 2H), 4.22 (brs, 1H), 4.02 (s, 1H), 3.17 (m, 1H), 2.84 (m, 2H), 2.10–1.96 (m, 2H), 1.81 (m, 2H), 1.56 (m, 2H), 0.88 (m, 5H); HRMS (ESI) calcd for C_28_H_27_N_6_O_3_ [M+H]^+^: 495.2139, found 495.6932.

(S)-Cyclopropyl(3-((4-(2-(3,4-dichlorophenyl)-6-hydroxy-1H-benzo[d]imidazol-1-yl) pyrimidin-2-yl)amino)piperidin-1-yl)methanone **(26d,** 57%); ^1^H NMR (400 MHz, CD_3_OD) δ 8.39 (d, J = 57.2 Hz, 1H), 7.75 (s, 1H), 7.58 (t, J = 8.2 Hz, 2H), 7.37 (d, J = 6.7 Hz, 1H), 7.05 (s, 1H), 6.91 (d, J = 8.8 Hz, 1H), 6.42 (d, J = 5.3 Hz, 1H), 4.24 (d, J = 12.4 Hz, 1H), 3.60 (s, 1H), 3.27–3.13 (m, 1H), 3.09–2.61 (m, 2H), 2.01 (s, 1H), 1.92–1.50 (m, 5H), 1.02–0.81 (m, 3H), 0.66–0.22 (m, 2H). HRMS (ESI) calcd for C_26_H_25_Cl_2_N_6_O_2_ [M+H]^+^: 523.1411, found 523.1561.

(S)-Cyclopropyl(3-((4-(2-(4-fluoro-3-(trifluoromethyl)phenyl)-6-hydroxy-1H-benzo[d] imidazol-1-yl)pyrimidin-2-yl)amino)piperidin-1-yl)methanone **(26e,** 65%); ^1^H NMR (400 MHz, CD_3_OD) δ 8.37 (s, 1H), 7.91 (d, J = 4.7 Hz, 1H), 7.77 (s, 1H), 7.67–7.54 (m, 1H), 7.42 (m, 1H), 7.02 (s, 1H), 6.90 (d, J = 8.7 Hz, 1H), 6.37 (s, 1H), 4.14 (s, 1H), 3.59 (s, 1H), 2.15–1.95 (m, 2H), 1.59 (m, 3H), 1.28 (m, 4H), 0.93–0.57 (m, 4H), 0.29 (m, 1H); HRMS(ESI) calcd for C_27_H_25_F_4_N_6_O_2_ [M+H]+: 541.1970, found 541.3818.

(S)-Cyclopropyl(3-((4-(6-hydroxy-2-(quinolin-2-yl)-1H-benzo[d]imidazol-1-yl)pyrimidin-2-yl)amino)piperidin-1-yl)methanone **(26f**, 83%); ^1^H NMR (400 MHz, MeOD) δ 8.45 (m, 2H), 8.35–8.15 (m, 1H), 7.97 (s, 1H), 7.79–7.57 (m, 3H), 7.22 (s, 1H), 6.97 (m, 1H), 6.77 (s, 1H), 4.07 (m, 2H), 3.25–3.08 (s, 1H), 2.69 (m, 1H), 2.08–1.96 (m, 1H), 1.87–1.49 (m, 4H), 1.29 (m, 3H), 0.89 (m, 3H), 0.65 (m, 1H); HRMS (ESI) calcd for C_29_H_28_N_7_O_2_ [M+H]+: 506.2299, found 506.4696.

(S)-(3-((4-(2-(Benzo[d][1,3]dioxol-5-yl)-6-hydroxy-1H-benzo[d]imidazol-1-yl)pyrimidin-2-yl)amino)piperidin-1-yl)(cyclopropyl)methanone **(26g,** 72%); ^1^H NMR (400 MHz, DMSO) δ 9.34 (d, J = 46.0 Hz, 2H), 8.48–8.23 (m, 1H), 7.70 (d, J = 32.8 Hz, 1H), 7.48 (d, J = 8.6 Hz, 1H), 6.96 (s, 1H), 6.76 (d, J = 6.1 Hz, 2H), 4.19 (d, J = 11.6 Hz, 1H), 3.88 (d, J = 68.9 Hz, 1H), 2.87 (d, J = 96.9 Hz, 1H), 2.11–1.75 (m, 3H), 1.74–1.37 (m, 3H), 1.20 (d, J = 23.5 Hz, 2H), 0.88–0.63 (m, 3H), 0.52 (d, J = 37.8 Hz, 1H), 0.14 (d, J = 90.2 Hz, 1H); HRMS (ESI) calcd for C_27_H_27_N_6_O_4_ [M+H]+: 506.2299, found 506.4381.

#### 3.4.4. Syntheses of cyclopropyl(3-((4-(5,6-dihydroxy-2-aryl-1H-benzo[d]imidazol-1-yl)pyrimidin-2-yl)amino)piperidin-1-yl)methanone (35)

Compound **34a** (0.053 mmol) was dissolved in methylene chloride (0.53 mL), 1 M boron tribromide (25µL) was added at −78 °C, and the reaction was stirred for 1 h and then at room temperature for 2 h. The mixture was quenched with methanol (0.2 mL) at 0 °C and stirred for an additional hour at room temperature. The mixture was diluted with methylene chloride (5 mL) and washed 3 times with saturated sodium bicarbonate solution (3 mL), 2 times with 5 mL of water, and 2 times with 5 mL of saturated sodium chloride solution. The organic phase was dried over sodium sulfate and concentrated in vacuo to obtain a white solid product. The crude product was purified by flash column chromatography on silica gel using a mobile phase of CH_2_Cl_2_: MeOH (40:1) to give product **35a** (as a yellow solid, 62%); ^1^H NMR (400 MHz, DMSO) δ 8.24 (s, 1H), 8.12 (s, 1H), 7.93 (m, 3H), 7.61–7.52 (m, 3H), 7.49 (s, 1H), 7.09 (s, 1H), 6.26 (d, J = 4.7 Hz, 1H), 3.83 (s, 1H), 3.41 (m, 2H), 3.17 (m, 2H), 2.94 (m, 2H), 1.99 (s, 1H), 1.43 (m, 4H), 0.89–0.66 (m, 4H), 0.57 (m, 1H); HRMS (ESI) calcd for C_30_H_29_N_6_O_3_ [M+H]+: 521.2296, found 521.0140.

(S)-Cyclopropyl(3-((4-(2-(2,3-dihydrobenzo[b][1,4]dioxin-6-yl)-5,6-dihydroxy-1H-benzo[d]imidazol-1-yl)pyrimidin-2-yl)amino)piperidin-1-yl)methanone **(35b,** as a yellow solid, 42%); ^1^H NMR (400 MHz, MeOD) δ 8.30 (s, 1H), 7.51 (s, 1H), 7.12 (d, J = 5.9 Hz, 1H), 7.02 (s, 1H), 6.95 (d, J = 2.9 Hz, 1H), 6.93–6.85 (m, 1H), 6.20 (d, J = 5.0 Hz, 1H), 4.69 (s, 1H), 4.29 (m, 4H), 3.93 (s, 1H), 3.57 (s, 1H), 3.22 (m, 1H), 2.87 (m, 1H), 1.96 (m, 3H), 1.61 (m, 3H), 1.31 (m, 1H), 1.06–0.62 (m, 4H), 0.34 (m, 1H); HRMS (ESI) calcd for C_28_H_28_N_6_O_5_ [M+H]+: 529.2194, found 529.3455.

(S)-(3-((4-(2-(Benzofuran-5-yl)-5,6-dihydroxy-1H-benzo[d]imidazol-1-yl)pyrimidin-2-yl)amino)piperidin-1-yl)(cyclopropyl)methanone **(35c,** as a white solid, 42%); ^1^H NMR (400 MHz, MeOD) δ 8.36–8.07 (m, 1H), 7.84 (d, J = 1.9 Hz, 1H), 7.80 (s, 1H), 7.56 (m, 2H), 7.41 (d, J = 7.7 Hz, 1H), 7.13 (d, J = 7.1 Hz, 1H), 6.90 (d, J = 4.7 Hz, 1H), 6.11 (m, 1H), 4.22 (s, 1H), 3.96 (s, 1H), 3.23 (s, 1H), 2.92 (m, 1H), 2.06–1.72 (m, 3H), 1.59 (m, 3H), 1.15 (m, 2H), 0.86 (m, 3H), 0.67–0.20 (m, 2H); ^13^C NMR (101 MHz, MeOD) δ 174.78 (s), 163.68 (s), 160.94 (s), 156.91 (s), 147.95 (s), 145.89 (d, J = 5.1 Hz), 145.25 (d, J = 6.1 Hz), 141.98 (s), 136.76 (s), 129.30 (s), 126.72 (d, J = 13.7 Hz), 123.61 (s), 112.51 (d, J = 6.1 Hz), 107.93 (s), 104.64 (s), 99.28 (s), 46.97 (s), 31.51 (s), 31.01 (s), 11.92 (d, J = 7.1 Hz), 8.10 (d, J = 17.1 Hz), 7.71 (s); HRMS (ESI) calcd for C_28_H_27_N_6_O_4_ [M+H]^+^: 511.2088, found 511.2906.

(S)-Cyclopropyl(3-((4-(2-(3,4-dichlorophenyl)-5,6-dihydroxy-1H-benzo[d]imidazol-1-yl) pyrimidin-2-yl)amino)piperidin-1-yl)methanone **(35d,** as a yellow solid, 58%); ^1^H NMR (400 MHz, DMSO) δ 9.16 (s, 1H), 8.44 (d, J = 44.8 Hz, 1H), 7.74 (s, 2H), 7.30 (d, J = 39.1 Hz, 1H), 7.08 (s, 1H), 6.57 (m, 1H), 4.26 (s, 1H), 3.91 (s, 1H), 2.99 (s, 1H), 2.84–2.53 (m, 1H), 2.08–1.86 (m, 1H), 1.66 (m, 2H), 1.51 (m, 2H), 1.23 (m, 3H), 0.88–0.65 (m, 3H), 0.57 (m, 1H), 0.25 (m, 1H); HRMS (ESI) calcd for C_26_H_25_Cl_2_N_6_O_3_ [M+H]+: 539.1360, found 541.2558.

(S)-Cyclopropyl(3-((4-(2-(4-fluoro-3-(trifluoromethyl)phenyl)-5,6-dihydroxy-1H-benzo[d] imidazol-1-yl)pyrimidin-2-yl)amino)piperidin-1-yl)methanone (**35e,** as a yellow solid, 60%); ^1^H NMR (400 MHz, MeOD) δ 8.36 (d, J = 68.0 Hz, 1H), 7.88 (d, J = 4.4 Hz, 1H), 7.75 (s, 1H), 7.48–7.37 (m, 1H), 7.13 (d, J = 6.5 Hz, 1H), 6.88 (d, J = 147.6 Hz, 1H), 6.34 (s, 1H), 4.60 (s, 1H), 4.31–3.82 (s, 2H), 3.13 (m, 1H), 2.76 (m, 1H), 2.06–1.70 (m, 3H), 1.60 (m, 2H), 1.29 (m, 2H), 1.01–0.54 (m, 4H), 0.32 (m, 1H); ^13^C NMR (101 MHz, MeOD) δ 174.76 (s), 163.74 (s), 162.69 (s), 161.68 (s), 158.97 (s), 146.61 (d, J = 10.4 Hz), 145.64 (d, J = 3.4 Hz), 136.83 (s), 136.39 (s), 129.04 (d, J = 14.7 Hz), 118.65 (s), 118.44 (s), 106.24 (s), 104.82 (s), 103.08 (s), 98.83 (s), 50.93 (s), 46.96 (s), 43.93 (s), 31.42 (s), 31.03 (s), 11.85 (s), 7.97 (d, J = 2.6 Hz); HRMS (ESI) calcd for C_27_H_25_F_4_N_6_O_3_ [M+H]^+^: 557.1919, found 557.2937.

## 4. Conclusions

In conclusion, we have successfully synthesized 1-(2-aminopyrimidin-4-yl)-2-aryl-1H-benzo[d]imidazole-5, 6-diol derivatives that were designed as potent JNK3-isoform selective inhibitors from previous lead. Thirty-seven compounds were synthesized and measured for their enzyme activity against JNK3. Particularly, compounds **26a**, **26d**, **35a**–**35e** showed competitive activities against JNK3 with IC_50_ values of about 20 nM. The most active series, **35a**–**35e**, especially showed good isoform selectivity. We believe that this novel scaffold, 5, 6-dihydroxy 3-alkyl-2-aryl-1-pyrimidinyl-1H-benzo[d]imidazole will be highly useful in the development of JNK3 selective inhibitors, as therapeutic agents for neurodegenerative diseases. Additionally, compound **35b,** (S)-cyclopropyl(3-((4-(2-(2, 3-dihydrobenzo[b][1,4]dioxin-6-yl)-5, 6-dihydroxy-1H-benzo[d]imidazol-1-yl)pyrimidin-2-yl)amino)piperidin-1-yl)methanone displayed the most potent inhibitory activity against JNK3, with an IC_50_ of 9.7 nM and an excellent selectivity profile, especially compared with the activity toward similar protein kinases such as GSK3β, Erk, JNK1, and JNK2. Additionally, **35b** showed 95.7% neuroprotection in Aβ-induced primary rat cortical neuronal death, showing a strong potential as a therapeutic in neurodegenerative diseases.

## Figures and Tables

**Table 1:** JNK3 inhibitory activity (IC_50_) of compounds **13a**–**13s**, **26a**–**26s** [18].

* 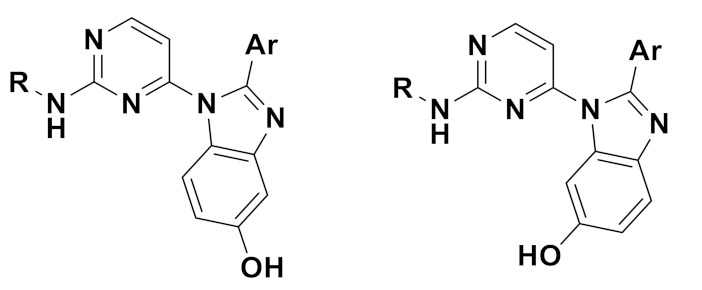 *							
9, 10, 13				22, 23, 26			
No	Ar	R	IC_50_, (μM)	No	Ar	R	IC_50_ (μM)
**9a**	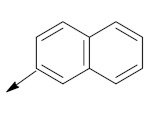	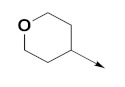	1.477	**22a**	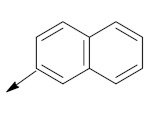	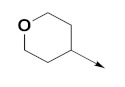	1.434
**10a**		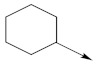	0.420	**23a**		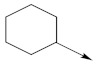	0.045
**13a**		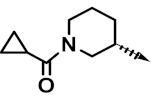	0.391	**26a**		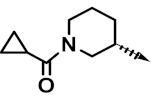	0.029
**9b**	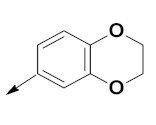	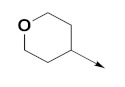	>10	**22b**	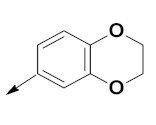	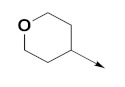	1.546
**10b**		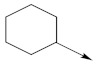	1.212	**23b**		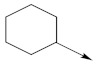	0.281
**13b**		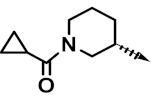	0.679	**26b**		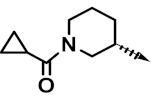	0.114
**9c**	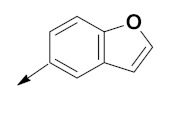	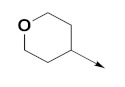	4.310	**22c**	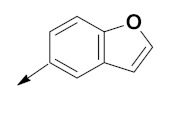	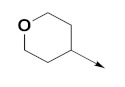	2.194
**10c**		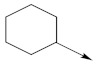	0.299	**23c**		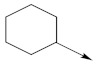	0.071
**13c**		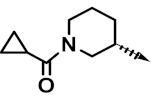	0.287	**26c**		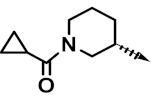	0.042
**10d**	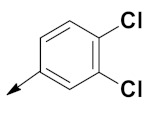	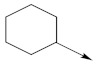	0.139	**23d**	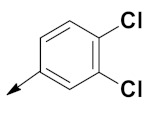	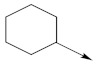	0.110
**13d**		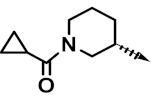	0.128	**26d**		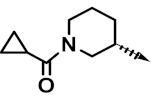	0.022
**10e**	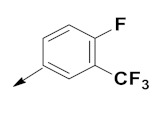	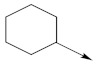	0.799	**23e**	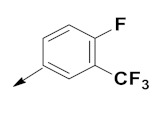	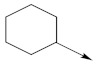	0.305
**13e**		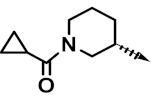	0.679	**26e**		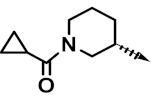	0.100
**10f**	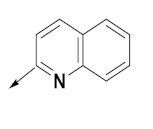	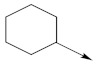	0.424	**23f**	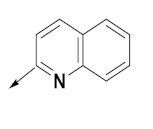	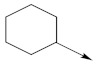	0.424
**13f**		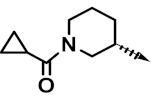	0.161	**26f**		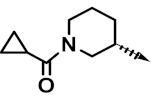	0.189
**13g**	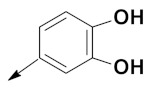	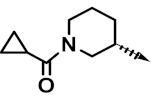	0.885	**26g**	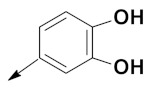	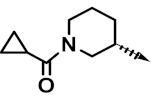	1.130
SP600125							0.0848

**Table 2:** JNK3 inhibitory activity (IC_50_)[18] of compounds **35a–35e**.

* 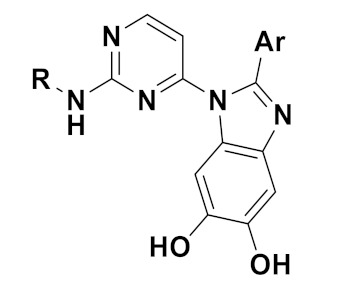 *					
35					
No	Ar	R	IC_50_ (μM)		
			JNK3	JNK2	JNK1
**35a**	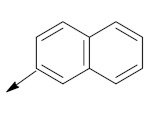	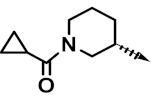	0.0199	0.266	2.434
**35b**	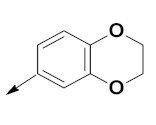		0.0097	0.898	>10
**35c**	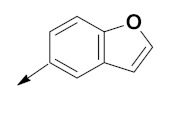		0.0209	0.248	2.596
**35d**	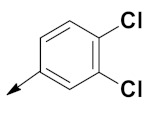		0.0287	0.293	1.228
**35e**	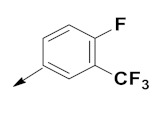		0.0421	0.508	>10
SP600125			0.0848	0.040	0.040

**Table 3:** Enzymatic activities of **35b** on selected protein kinases.

Kinase	IC_50_ (μM)		Selectivity/JNK3
	35b	Control	
GSK3β	5.85	2.30 (Staurosporine)	>600 fold
JNK3	0.0097	0.0848 (SP600125)	

**Table 4:** Cell viability (%) from Aβ-induced neurotoxicity in primary rat cortical neuron.

Cell Viability	Mean	Δ
Vehicle	60.8 ± 1.3	0
**35a**	88.9 ± 3.8	28.1
**35b**	95.7 ± 2.9	34.9
**35c**	93.4 ± 2.5	32.6
**35d**	82.2 ± 3.8	21.4
**35e**	81.0 ± 3.2	20.2
Resveratrol	101.8 ± 1.2	40.9

## Supplementary Materials

The following are available online at https://www.mdpi.com/article/10.3390/ijms222011084/s1. Figure S1: Effect of selective JNK3 inhibitors on amyloid-β-induced apoptosis in primary rat neuron, Figure S2: Cytotoxic effect of JNK3 inhibitors in neurons.
