# Supplementary material for: Discovery of a Potent and Selective JNK3 Inhibitor with Neuroprotective Effect Against Amyloid β-Induced Neurotoxicity in Primary Rat Neurons
Source: Int J Mol Sci. 2021 Oct 14;22(20):11084. doi: 10.3390/ijms222011084 (PMC8539420; doi:10.3390/ijms222011084)

Supporting Information

Discovery of a potent and selective JNK3 inhibitor with neuroprotective effect against amyloid β-induced neurotoxicity in primary rat neurons

Joonhong Jun[1,2 †], Jihyun Baek[1,2 †],Songyi Yang[1, 2], Hyungwoo Moon[1,2], Hyejin Kim[1,2], Hyunwook Cho[1,2], and Jung-Mi Hah[1,2*]

[1] Department of Pharmacy, College of Pharmacy, Hanyang University, Ansan, Korea
[2] Institute of Pharmaceutical Science and Technology, Center for Proteinopathy, Hanyang University, Ansan, Korea
* Correspondence: jhah@hanyang.ac.kr; Tel.: +82-31-400-5803
† These authors contributed equally to this work.

1. Molecular Modelling

Compounds were docked into the JNK3 structure (PDB: 4KKH). Protein and ligand preparations were performed with Schrödinger's tools with standard settings and Glide was used for docking and scoring. The 3D X-ray protein structures of JNK3 as a complex with a ligand were obtained from the PDB (code: 4KKH) and prepared using the Protein Preparation Wizard of the Schrödinger Maestro program. All water molecules were removed from the structure and it was selected as a template. The structures of inhibitors were drawn using Chemdraw, and their 3D conformation was generated using the Schrödinger LigPrep program with the OPLS 2005 force field. Molecular docking of compound into the structure of JNK3 (PDB code: 4KKH) were carried out using Schrodinger Glide (Version 12.7).

2. Chemistry

All chemicals were of reagent grade and were purchased from Aldrich (USA), TCI (Japan), Alfa Aesar (USA). Purification of the compounds by column chromatography was carried out with silica gel 60 (200–300 mesh ASTM, E. Merck, Germany). The quantity of silica gel used was 50–100 times the weight charged on the column. Thin layer chromatography (TLC) was run on the silica gel-coated aluminum sheets (silica gel 60 GF254, E. Merck, Germany) and visualized under ultraviolet (UV) light (254 nm). ^{1}H NMR and ^{13}C NMR spectra were recorded on a Brucker model digital AVANCE III 400 MHz spectrometer at 25 °C using tetramethylsilane (TMS) as an internal standard. High-resolution MS (HR/MS) experiments were conducted with Q-TOF/Mass spectrometer 6530 (Agilent Technologies, Santa Clara, CA, USA) operated in positive-ion electrospray mode.

2.1. Syntheses of 1-(2-(alkylamino)pyrimidin-4-yl)-2-aryl-1H-benzo[d]imidazol-5 or 6-ol derivatives (Scheme 1)

2.1.1. N-(4-methoxy-2-nitrophenyl)-2-(methylthio)pyrimidin-4-amine (*2*)

To the solution of compound **1** (3 g, 17.85 mmol) in DMF (89 ml), 60% NaH (893 mg) was slowly added at 0 °C, stirred for 1 h, and then 4-chloro-2-(methylthio)pyrimidine (5.73 g, 17.85 mmol) was added and stirred for about 2 h. Then, the solvent was poured into ice water to precipitate. The precipitated product was filtered to obtain the target compound **2** (4 g, 78%) as a residue. ^{1}H NMR (400 MHz, $CDCl_3$) δ 9.78 (s, 1H), 8.58 (d, *J* = 9.3 Hz, 1H), 8.19 (d, *J* = 5.8 Hz, 1H), 7.65 (d, *J* = 3.0 Hz, 1H), 7.24 (dd, *J* = 9.3, 3.0 Hz, 1H), 6.47 (d, *J* = 5.8 Hz, 1H), 3.86 (s, 3H), 2.52 (s, 3H); ^{13}C NMR (101 MHz, DMSO-d_6)

δ 161.6, 159.1, 149.2, 141.1, 145.5, 137.3, 136.7, 133.5, 127.3, 124.4, 121.3, 99.3, 88.9, 70.5, 36.4, 33.6; mp: 58.5–60.5 °C; HRMS (ES+) *m/z* calcd for $C_{12}H_{13}N_4O_3S$ 293.0703, Found 293.3762.
Compound **15** was obtained as a white solid in **15** (6 g, 77 %) ; ^{1}H NMR (400 MHz, $CDCl_3$)δ10.65(s,1H),8.63(d,*J* = 2.7 Hz, 1H), 8.26 (d, *J* = 5.7 Hz, 1H), 8.22 (d, *J* = 9.5 Hz, 1H), 6.59 (dd, *J* = 9.5, 2.7 Hz, 1H), 6.50 (d, *J* = 5.7 Hz, 1H), 3.93 (s, 3H), 2.57 (s, 3H). ; HRMS (ES+) *m/z* calcd for $C_{12}H_{13}N_4O_3S$ 293.0703, Found 293.3762.

2.1.2. 4-Methoxy-N¹-(2-(methylthio)pyrimidin-4-yl)benzene-1,2-diamine (3)

Compound **2** (1433 mg, 4.9 mmol) was dissolved in MeOH (33 ml), then 10% Pd/C (143 mg) was added and stirred at room temperature for 5 h under hydrogen gas. After the reaction was completed, it was filtered with celite and the filtrate was distilled under reduced pressure. The residue was obtained without further purification of the target compound 3 (1280 mg, 99%). ^{1}H NMR (400 MHz, DMSO) δ 8.54 (s, 1H), 7.95 (d, *J* = 5.9 Hz, 1H), 6.93 (d, *J* = 5.9 Hz, 1H), 6.33 (d, *J* = 2.8 Hz, 1H), 6.15 (dd, *J* = 8.6, 2.8 Hz, 1H), 5.98 (s, 1H), 4.94 (s, 2H), 3.67 (s, 3H), 2.39 (s, 3H). HRMS (ES+) *m/z* calcd for $C_{12}H_{15}N_4OS$ 263.4336
Compound **16** was obtained as a white solid (2.9 g, 99 %); ^{1}H NMR (400 MHz, $CDCl_3$)δ7.99(d,*J* = 5.9 Hz, 1H), 7.02 (s, 1H), 6.78 (d, *J* = 2.8Hz, 1H), 6.74 (d, *J* = 8.7 Hz, 1H), 6.68 (dd, *J* = 8.7, 2.8 Hz, 1H), 6.07 (d, *J* = 5.9 Hz, 1H), 3.78 (s, 2H), 3.69 (s, 3H), 2.46 (d, *J* = 5.1 Hz, 3H); HRMS (ES+) *m/z* calcd for $C_{12}H_{15}N_4OS$ 263.0961, Found 263.4336.

2.1.3. General synthesis of 5-methoxy-1-(2-(methylthio)pyrimidin-4-yl)-2-(naphthalen-2-yl)-1H-benzo[d]imidazole (4a-4g)

Compound **3** (400 mg, 1.52 mmol), 2-naphthaldehyde (260 mg, 1.67 mmol), $Na_2S_2O_5$ (1.45g), DMF (3ml) was added and stirred in a microwave at 120 °C, 150 W, 1 h 30 min. After the completion of the reaction, the solvent was poured into ice water to precipitate. After filtering the precipitated reaction product, the filtrate was distilled under reduced pressure, and the residue was purified by column chromatography (silica gel, n-hexane: ethyl acetate = 3: 1) to obtain the target compound **4a** (370 mg, 50%). ^{1}H NMR (400 MHz, $CDCl_3$) δ 8.35 (d,*J* = 5.4 Hz, 1H), 8.21 (d, *J* = 1.3 Hz, 1H), 7.88 (d, *J* = 1.8 Hz, 1H), 7.86 – 7.84 (m, 2H), 7.83 (s, 1H), 7.59 – 7.49 (m, 3H), 7.37 (d, *J* = 2.5 Hz, 1H), 7.02 (dd, *J* = 9.0, 2.5 Hz, 1H), 6.53 (d, *J* = 5.4 Hz, 1H), 3.90 (s, 3H), 2.48 (s, 3H).; HRMS (ES+) *m/z* calcd for $C_{23}H_{19}N_4OS$ 399.4915, Found 399.5515.
Compound **4b** was obtained as a white solid (403 mg, 59%) by the same procedure as above. ^{1}H NMR (400 MHz, $CDCl_3$) δ 8.42 (d,*J* = 5.4 Hz, 1H), 7.76 (d, *J* = 8.9 Hz, 1H), 7.30 (d, *J* = 2.3 Hz, 1H), 7.13 (d, *J* = 2.1 Hz, 1H), 7.00 – 6.93 (m, 2H), 6.86 (d, *J* = 8.4 Hz, 1H), 6.56 (d, *J* = 5.4 Hz, 1H), 4.28 (m, 2H), 4.27 – 4.23 (m, 2H), 3.87 (s, 3H), 2.54 (s, 3H). ; HRMS (ES+) *m/z* calcd for $C_{21}H_{19}N_4O_3S$ 407.1172, Found 407.5874.
4c (325 mg, 64 %); ^{1}H NMR (400 MHz, $CDCl_3$) δ 8.24(d,*J* = 5.4 Hz, 1H), 7.77 (d, *J* = 1.4 Hz, 1H), 7.70 (d, *J* = 8.9 Hz, 1H), 7.59 (d, *J* = 2.2 Hz, 1H), 7.43 (d, *J* = 8.6 Hz, 1H), 7.35 (dd, *J* = 8.6, 1.7 Hz, 1H), 7.25 (d, *J* = 2.4 Hz, 1H), 6.88 (dd, *J* = 8.9, 2.5 Hz, 1H), 6.70 (dd, *J* = 2.2, 0.8 Hz, 1H), 6.37 (d, *J* = 5.4 Hz, 1H), 3.79 (s, 3H), 2.39 (s, 3H).; HRMS (ES+) *m/z* calcd for $C_{21}H_{16}N_4O_2S$ 389.4525, Found 389.5610.
4d (YSI0507801) (588 mg, 86 %); ^{1}H NMR (400 MHz, $CDCl_3$) δ 8.45 (d, *J* = 5.3 Hz, 1H), 7.74 (d, *J* = 2.3 Hz, 1H), 7.66 (d, *J* = 9.0 Hz, 1H), 7.40 (d, *J* = 8.3 Hz, 1H), 7.27 – 7.20 (m, 2H), 6.95 (dd, *J* = 9.0, 2.3 Hz, 1H), 6.59 (d, *J* = 5.3 Hz, 1H), 3.83 (s, 3H), 2.41 (s, 3H). HRMS (ES+) *m/z* calcd for $C_{19}H_{14}Cl_2N_4OS$ 417.0338, Found 417.1042.
393.0598
4e (370 mg, 56 %); ^{1}H NMR (400 MHz, $CDCl_3$)δ8.48(d,*J* = 5.3 Hz, 1H), 7.94 (dd, *J* = 6.5, 1.5 Hz, 1H), 7.60 (d, *J* = 9.0 Hz, 1H), 7.58 – 7.54 (m, 1H), 7.24 (d, *J* = 2.2 Hz, 1H), 7.17 (m, 1H), 6.93 (dd, *J* = 9.0, 2.2 Hz, 1H), 6.67 (d, *J* = 5.3 Hz, 1H), 3.81 (s, 3H), 2.34 (s, 3H). HRMS (ES+) *m/z* calcd for $C_{20}H_{15}F_4N_4OS$ 435.0897,

Found 435.1922

4f (360mg, 59%), ^{1}H NMR (400 MHz, $CDCl_3$) δ 8.50 (d,*J* = 5.3 Hz, 1H), 8.32 (d, *J* = 8.5 Hz, 1H), 8.23 (d, *J* = 8.5 Hz, 1H), 7.78 (d, *J* = 8.1 Hz, 1H), 7.60 (dd, *J* = 4.6, 2.0 Hz, 2H), 7.55 (d, *J* = 9.0 Hz, 1H), 7.50 (m, 1H), 7.35 (d, *J* = 2.4 Hz, 1H), 7.00 (dd, *J* = 9.0, 2.4 Hz, 1H), 6.83 (d, *J* = 5.3 Hz, 1H), 3.86 (s, 3H), 2.36 (s, 3H).**;** H RMS (ES$^+$) *m/z* calcd for $C_{22}H_{18}N_5OS$ 400.1227, Found 400.5284.

4g (210 mg, 34 %); ^{1}H NMR (400 MHz, $CDCl_3$) δ 8.43(d,*J* = 5.4 Hz, 1H), 7.78 (d, *J* = 8.9 Hz, 1H), 7.30 (d, *J* = 2.4 Hz, 1H), 7.04 (d, *J* = 1.4 Hz, 1H), 7.02 (dd, *J* = 8.0, 1.7 Hz, 1H), 6.97 (dd, *J* = 9.0, 2.5 Hz, 1H), 6.82 (d, *J* = 8.0 Hz, 1H), 6.56 (d, *J* = 5.4 Hz, 1H), 6.02 (s, 2H), 3.88 (s, 3H), 2.55 (s, 3H). HRMS (ES$^+$) *m/z* calcd f or $C_{20}H_{17}N_4O_3S$ 393.1016, Found 393.0598.

17a (80 mg, 33 %); ^{1}H NMR (400 MHz, CDCl3) d 8.68 (d, *J* = 5.6 Hz, 1H), 8.65 (d, *J* = 5.6 Hz, 1H), 8.22 (d, *J* = 1.2 Hz, 1H), 8.19 (d, *J* = 1.2 Hz, 1H), 7.95–8.00 (m, 6H), 7.80 (d, *J* = 8.8 Hz, 1H), 7.75 (d, *J* = 8.8 Hz, 1H), 7.54–7.64 (m, 6H), 7.41 (d, *J* = 2.4 Hz,1H), 7.39 (d, J = 2.4 Hz, 1H), 7.11 (d, J = 5.6 Hz, 1H), 7.03–7.06 (m, 3H), 3.86 (s, 3H), 3.84 (s, 3H), 2.28 (s, 3H), 2.27 (s, 3H).; HRMS (ES$^+$) *m/z* calcd for $C_{23}H_{19}N_4OS$ 399.4915, Found 399.5515.

17b (423 mg, 63 %); ^{1}H NMR (400 MHz, $CDCl_3$) δ 8.42 (d,*J* = 5.4 Hz, 1H), 7.68 (d, *J* = 8.8 Hz, 1H), 7.42 (d, *J* = 2.4 Hz, 1H), 7.10 (d, *J* = 2.1 Hz, 1H), 6.96 (m, 2H), 6.85 (d, *J* = 8.4 Hz, 1H), 6.54 (d, *J* = 5.4 Hz, 1H), 4.28 (dd, *J* = 3.6, 1.7 Hz, 2H), 4.25 (dd, *J* = 3.6, 1.7 Hz, 2H), 3.84 (s, 3H), 2.56 (s, 3H).; HRMS (ES$^+$) *m/z* calcd for $C_{21}H_{19}N_4O_3S$ 407.1172, Found 407.5874

17c (262 mg, 45 %); ^{1}H NMR (400 MHz, $CDCl_3$)δ8.32(d,*J* = 5.4 Hz, 1H), 7.81 (d, *J* = 1.8 Hz, 1H), 7.70 (d, *J* = 8.8 Hz, 1H), 7.64 (d, *J* = 2.2 Hz, 1H), 7.48 (d, *J* = 8.6 Hz, 1H), 7.43 (d, *J* = 2.5 Hz, 1H), 7.39 (dd, *J* = 8.6, 1.8 Hz, 1H), 6.97 (dd, *J* = 8.8, 2.5 Hz, 1H), 6.75 (dd, *J* = 2.2, 0.9 Hz, 1H), 6.44 (d, *J* = 5.4 Hz, 1H), 3.83 (s, 3H), 2.49 (s, 3H); HRMS (ES$^+$) *m/z* calcd for $C_{21}H_{16}N_4O_2S$ 389.4525, Found 389.5618

17d (562 mg, 67 %); ^{1}H NMR (400 MHz, $CDCl_3$) δ8.50 (d, *J* = 5.3 Hz, 1H), 7.77 (d, *J* = 2.0 Hz, 1H), 7.70 (d, *J* = 8.8 Hz, 1H), 7.42 (d, *J* = 8.3 Hz, 1H), 7.33 (d, *J* = 2.4 Hz, 1H), 7.23 (dd, *J* = 8.3, 2.0 Hz, 1H), 6.99 (dd, *J* = 8.8, 2.4 Hz, 1H), 6.62 (d, *J* = 5.3 Hz, 1H), 3.84 (s, 3H), 2.49 (s, 3H).; HRMS (ES$^+$) *m/z* calcd for $C_{19}H_{14}Cl_2N_4OS$ 417.0338, Found 418.7429.

17e (370 mg, 56 %); ^{1}H NMR (400 MHz, $CDCl_3$)δ8.43(d,*J* = 5.3 Hz, 1H), 7.86 (dd, *J* = 6.6, 1.9 Hz, 1H), 7.60 (d, *J* = 8.8 Hz, 1H), 7.47 (m, 1H), 7.19 (d, *J* = 2.4 Hz, 1H), 7.10 (m, 1H), 6.90 (dd, *J* = 8.8, 2.4 Hz, 1H), 6.59 (d, *J* = 5.3 Hz, 1H), 3.75 (s, 3H), 2.33 (s, 3H).; HRMS (ES$^+$) *m/z* calcd for $C_{20}H_{15}F_4N_4OS$ 435.0897, Found 435.4758.

17f (300 mg, 57 %); ^{1}H NMR (400 MHz, DMSO) δ 9.13 (d, *J* = 5.5 Hz, 1H), 7.95 (d, *J* = 2.0 Hz, 1H), 7.76 – 7.69 (m, 2H), 7.64 (t, *J* = 4.4 Hz, 2H), 7.51 – 7.47 (m, 1H), 7.06 (dd, *J* = 8.8, 2.4 Hz, 1H), 3.83 (d, *J* = 3.2 Hz, 3H), 3.37 (s, 3H).; HRMS (ES$^+$) *m/z* calcd for $C_{22}H_{18}N_5OS$ 400.1227, Found 400.5600.

17g (414 mg, 64 %); ^{1}H NMR (400 MHz, $CDCl_3$)δ8.45(d,*J* = 5.4 Hz, 1H), 7.70 (d, *J* = 8.8 Hz, 1H), 7.43 (d, *J* = 2.4 Hz, 1H), 7.03 (d, *J* = 1.5 Hz, 1H), 7.00 (m, 2H), 6.82 (d, *J* = 8.0 Hz, 1H), 6.56 (d, *J* = 5.4 Hz, 1H), 6.03 (s, 2H), 3.86 (s, 3H), 2.58 (s, 3H).; HRMS (ES$^+$) *m/z* calcd for $C_{20}H_{17}N_4O_3S$ 393.1016, Found 393.1859.

2.1.4. 5-methoxy-1-(2-(methylsulfonyl)pyrimidin-4-yl)-2-(naphthalen-2-yl)-1H-benzo[d]imidazole (5a)

Compound **4a** (500 mg, 1.25 mg) and Potassium peroxomonosulfate (3.8 g) were added to MeOH: H_2O=1:1 (7ml) and stirred at room temperature for 1 hour. After confirming the completion of the reaction, MeOH was distilled under reduced pressure. Water to the distilled mixture. After addition, it was diluted and stirred until the product was separated into a solid. The solid product was filtered and washed with water. The crude product was then crystallized to obtain the title compound **5a** (530 mg, 98%). ; ^{1}H NMR (400 MHz, DMSO) δ 9.00 (d, *J* = 5.6 Hz, 1H), 8.30 (s, 1H), 8.02 (d, *J* = 8.9 Hz, 4H), 7.68 – 7.58 (m, 3H), 7.44 (d, *J* = 5.6 Hz, 1H), 7.41 (t, *J* = 3.5 Hz, 1H), 7.10 (dd, *J* = 9.1, 2.4 Hz, 1H), 3.87 (s, 3H), 3.31 (s, 3H) ; HRMS *m/z* calcd for $C_{23}H_{19}N_4O_3S$ 431.1172, Found 431.8835.

5b (315 mg, 90 %); ^{1}H NMR (400 MHz, DMSO) δ 9.12 (d, *J* = 5.5 Hz, 1H), 7.93 (d, *J* = 9.0 Hz, 1H), 7.51 (d, *J* = 5.5 Hz, 1H), 7.37 (d, *J* = 2.4 Hz, 1H), 7.19 (d, *J* = 2.0 Hz, 1H), 7.10 (dd, *J* = 9.0, 2.4 Hz, 1H), 7.02 (dd, *J* =

8.4, 2.0 Hz, 1H), 6.97 (d, J = 8.4 Hz, 1H), 4.33 (d, J = 4.7 Hz, 2H), 4.30 (d, J = 4.7 Hz, 2H), 3.86 (s, 3H), 3.41 (s, 3H).**;** HRMS *m*/*z* calcd for $C_{21}H_{19}N_4O_5S$ 438.1071, Found 438.9739.
5c (356 mg, 89 %) ^{1}H NMR (400 MHz, DMSO) δ 9.07 (d, J = 5.5 Hz, 1H), 8.15 (d, J = 2.4 Hz, 1H), 8.03 (d, J = 1.7 Hz, 1H), 8.01 (d, J = 9.1 Hz, 1H), 7.76 (d, J = 8.6 Hz, 1H), 7.56 (dd, J = 8.6, 1.7 Hz, 1H), 7.43 (m, 2H), 7.16 (dd, J = 9.1, 2.4 Hz, 1H), 7.09 (d, J = 1.5 Hz, 1H), 3.88 (s, 3H), 3.36 (s, 3H). HRMS *m*/*z* calcd for $C_{21}H_{17}N_4O_4S$ 421.0965, Found 421.2323.
5d (220 mg, 95 %); ^{1}H NMR (400 MHz, DMSO) δ 9.10 (d, J = 5.5 Hz, 1H), 7.97 – 7.94 (m, 2H), 7.71 (d, J = 8.4 Hz, 1H), 7.60 (d, J = 5.5 Hz, 1H), 7.52 (dd, J = 8.4, 1.9 Hz, 1H), 7.38 (d, J = 2.3 Hz, 1H), 7.09 (dd, J = 9.0, 2.3 Hz, 1H), 3.85 (s, 3H), 3.35 (s, 3H). HRMS *m*/*z* calcd for $C_{19}H_{15}Cl_2N_4O_3S$ 449.0236, Found 449.0573
5e (348 mg, 94 %); ^{1}H NMR (400 MHz, DMSO) δ 9.12 (d, J = 5.5 Hz, 1H), 8.10 (d, J = 5.9 Hz, 1H), 7.96 – 7.88 (m, 2H), 7.66 (d, J = 5.5 Hz, 1H), 7.61 (d, J = 9.7 Hz, 1H), 7.39 (d, J = 2.1 Hz, 1H), 7.08 (dd, J = 9.0, 2.1 Hz, 1H), 3.85 (s, 3H), 3.33 (s, 3H). HRMS (ES^+) *m*/*z* calcd for $C_{20}H_{15}F_4N_4O_3S$ 467.0796, Found 467.4914.
5f (305 mg, 99 %) ; ^{1}H NMR (400 MHz, DMSO) δ 9.17 (s, 1H), 8.59 (d, J = 7.7 Hz, 1H), 8.39 (d, J = 7.5 Hz, 1H), 8.04 (d, J = 6.9 Hz, 1H), 7.94 (s, 1H), 7.71 (m, 2H), 7.64 (s, 1H), 7.45 (s, 1H), 7.38 (d, J = 7.2 Hz, 1H), 7.10 (d, J = 7.7 Hz, 1H), 3.86 (s, 3H), 3.35 (s, 3H).; HRMS (ES^+) *m*/*z* calcd for $C_{22}H_{18}N_5O_3S$ 432.1125, Found 432.5137.
5g (200 mg, 87 %); ^{1}H NMR (400 MHz, DMSO) δ 9.11 (d, J = 5.5 Hz, 1H), 7.96 (d, J = 8.1 Hz, 1H), 7.49 (d, J = 5.5 Hz, 1H), 7.37 (d, J = 2.3 Hz, 1H), 7.21 (d, J = 1.5 Hz, 1H), 7.13 – 7.08 (m, 2H), 7.05 (d, J = 8.1 Hz, 1H), 6.15 (s, 2H), 3.86 (s, 3H), 3.42 (s, 3H). HRMS (ES^+) *m*/*z* calcd for $C_{20}H_{17}N_4O_5S$ 425.0914, Found 425.1399.
18a (250 mg, 98 %); ^{1}H NMR (400 MHz, CDCl3) δ9.01 (d, J = 5.6 Hz, 1H), 8.26 (d, J = 1.6 Hz, 1H), 8.01 (d, J = 8.4 Hz,1H),7.77 (d, J = 8.4 Hz, 1H), 7.73 (d, J = 2.4 Hz, 1H), 7.57–7.65 (m, 3H),7.47 (d, J = 5.6 Hz, 1H), 7.08 (dd, J = 8.4 Hz, J = 2.4 Hz, 1H), 3.85 (s, 3H), 3.30 (s, 3H). HRMS *m*/*z* calcd for $C_{23}H_{19}N_4O_3S$ 431.1172, Found 431.2217.
18b (432 mg, 98 %); ^{1}H NMR (400 MHz, DMSO) δ 9.17 (d, J = 5.3 Hz, 1H), 7.82 (d, J = 9.0 Hz, 1H), 7.60 (d, J = 2.0 Hz, 1H), 7.57 (d, J = 5.3 Hz, 1H), 7.20 (s, 1H), 7.17 (dd, J = 9.0, 2.0 Hz, 1H), 7.02 (d, J = 1.6 Hz, 1H), 6.99 (s, 1H), 4.33 (s, 2H), 4.30 (s, 2H), 3.81 (s, 3H), 2.90 (s, 3H). HRMS *m*/*z* calcd for $C_{21}H_{19}N_4O_5S$ 438.1071, Found 439.1627.
18c (153 mg, 95 %); ^{1}H NMR (400 MHz, DMSO) δ 9.08 (d, J = 5.5 Hz, 1H), 8.14 (d, J = 2.2 Hz, 1H), 7.99 (d, J = 1.4 Hz, 1H), 7.78 (d, J = 8.8 Hz, 1H), 7.76 – 7.73 (m, 1H), 7.70 (d, J = 2.2 Hz, 1H), 7.53 (dd, J = 8.8, 1.4 Hz, 1H), 7.45 (d, J = 5.5 Hz, 1H), 7.13 (dd, J = 8.9, 2.3 Hz, 1H), 7.07 (d, J = 1.4 Hz, 1H), 3.84 (s, 3H), 3.38 (s, 3H). HRMS *m*/*z* calcd for $C_{21}H_{17}N_4O_4S$ 421.0965, Found 421.2639.
18d (526 mg, 98 %); ^{1}H NMR (400 MHz, DMSO) δ 9.13 (d, J = 5.5 Hz, 1H), 7.95 (d, J = 2.0 Hz, 1H), 7.74 (d, J = 8.8 Hz, 1H), 7.70 (d, J = 8.4 Hz, 1H), 7.64 (m, 2H), 7.49 (dd, J = 8.4, 2.0 Hz, 1H), 7.06 (dd, J = 8.8, 2.4 Hz, 1H), 3.83 (s, 3H), 3.37 (s, 3H).; HRMS *m*/*z* calcd for $C_{19}H_{15}Cl_2N_4O_3S$ 449.0236, Found 449.1204.
18e (343 mg, 96 %);^{1}H NMR (400 MHz, DMSO) δ 9.15 (d, J = 5.5 Hz, 1H), 8.09 – 8.06 (m, 1H), 7.89 – 7.84 (m, 1H), 7.75 (d, J = 8.8 Hz, 1H), 7.69 (d, J = 5.5 Hz, 1H), 7.63 (d, J = 2.4 Hz, 1H), 7.58 (d, J = 10.2 Hz, 1H), 7.07 (dd, J = 8.8, 2.4 Hz, 1H), 3.83 (s, 3H), 3.35 (s, 3H).; HRMS (ES^+) *m*/*z* calcd for $C_{20}H_{15}F_4N_4O_3S$ 467.0796, Found 467.5229.
18f (283 mg, 98 %); ^{1}H NMR (400 MHz, DMSO) δ 9.17 (d, J = 5.3 Hz, 2H), 8.57 – 8.53 (m, 2H), 8.37 (t, J = 7.6 Hz, 2H), 8.02 (d, J = 8.0 Hz, 2H), 7.87 (d, J = 5.3 Hz, 1H), 7.81 (d, J = 8.9 Hz, 2H), 7.71 (t, J = 7.6 Hz, 2H), 7.62 (t, J = 7.4 Hz, 2H), 7.37 – 7.26 (m, 4H), 7.09 – 7.04 (m, 2H), 3.80 (s, 6H), 2.73 (s, 2H). HRMS (E S^+) *m*/*z* calcd for $C_{22}H_{18}N_5O_3S$ 432.1125, Found 432.1356.
18g (404 mg, 95 %); ^{1}H NMR (400 MHz, DMSO) δ 9.16 (s, 1H), 8.15 – 8.13 (m, 2H), 7.76 (d, J = 7.5 Hz, 1H), 7.69 – 7.50 (m, 2H), 7.21 (s, 1H), 7.17 – 7.02 (m, 1H), 6.15 (s, 2H), 3.83 (s, 3H), 3.44 (s, 3H). HRMS (ES^+) *m*/*z* calcd for $C_{20}H_{17}N_4O_5S$ 425.0914, Found 425.1892

2.1.5. 4-(5-methoxy-2-(naphthalen-2-yl)-1H-benzo[d]imidazol-1-yl)-N-(tetrahydro-2H-pyran-4-yl)pyrimidin -2-amine (6a)

Compound **5a** (43 mg, 0.1 mmol) and tetrahydro-2*H*-pyran-4-amine (21 ul) were stirred in THF (1ml) at 60°C for 5 hours. After confirming the completion of the reaction, the reaction mixture was cooled to ambient temperature, the filtrate was distilled under reduced pressure, and the residue was purified by column chromatography (silica gel, n-hexane: ethyl acetate = 2: 1) to obtain the target compound **6a** (31 mg, 68%).; ^{1}H NMR (400 MHz, $CDCl_3$)δ8.27(s,1H),8.23(s,1H),7.92–7.76(m,3H),7.67(s,1H),7.56-7.45(m,3H),7.38(d,*J* = 1.9 Hz, 1H), 6.99 (dd, *J* = 8.9, 2.4 Hz, 1H), 6.38 (d, *J* = 160.1 Hz, 1H), 5.47 (s, 1H), 3.90 (s, 3H), 3.50 (m, 2H), 3.39 – 3.21 (m, 1H), 2.93 (m, 1H), 1.58 – 1.30 (m, 2H), 1.24 (m, 2H), 1.15 – 0.81 (m, 1H). ; HRMS *m/z* calcd for $C_{27}H_{26}N_5O_2$ 452.2081, Found 452.5236.
6b (44 mg, 83 %);^{1}H NMR (400 MHz, $CDCl_3$) δ 8.26(d,*J* = 4.5 Hz, 1H), 7.59 (s, 1H), 7.31 (d, *J* = 2.0 Hz, 1H), 7.13 (s, 1H), 7.04 (dd, *J* = 8.4, 2.0 Hz, 1H), 6.92 (dd, *J* = 8.9, 2.3 Hz, 1H), 6.85 (d, *J* = 8.4 Hz, 1H), 6.40 (s, 1H), 4.25 (dd, *J* = 11.3, 4.9 Hz, 4H), 3.95 (s, 1H), 3.86 (s, 3H), 3.49 – 3.31 (m, 2H), 1.76 (m, 2H), 1.50 (m, 3H), 1.30 – 1.18 (m, 1H), 1.01 – 0.78 (m, 1H). HRMS *m/z* calcd for $C_{25}H_{26}N_5O_4$ 460.1979, Found 460.6220.
6c (36 mg, 66 %); ^{1}H NMR (400 MHz, $CDCl_3$)δ8.24(s,1H),7.91(d,*J* = 1.0 Hz, 1H), 7.67 (d, *J* = 2.2 Hz, 1H), 7.66 – 7.55 (s, 1H), 7.50 (d, *J* = 8.6 Hz, 1H), 7.44 (d, *J* = 8.4 Hz, 1H), 7.36 (d, *J* = 2.2 Hz, 1H), 6.97 (dd, *J* = 8.9, 2.4 Hz, 1H), 6.80 (dd, *J* = 2.1, 1.0 Hz, 1H), 6.45 (s, 1H), 3.88 (s, 3H), 3.76 (m, 1H), 3.52 (s, 1H), 3.17 (m, 1H), 2.13 – 1.84 (m, 1H), 1.56 (m, 2H), 1.48 – 1.28 (m, 2H), 1.27 – 1.20 (m, 1H), 1.03 – 0.74 (m, 1H). HRMS *m/z* calcd for $C_{25}H_{24}N_5O_3$ 442.1874, Found 442.6605.
19a (34 mg, 79 %); ^{1}H NMR (400 MHz, $CDCl_3$)δ8.28(s,1H),8.22(s,1H),7.87–7.83(m,1H),7.83–7.76(m,3H),7.54–7.49(m,2H),7.47(dd,*J* = 8.7, 4.9 Hz, 1H), 7.25 (d, *J* = 7.3 Hz, 1H), 7.02 (dd, *J* = 8.8, 2.4 Hz, 1H), 6.56 (s, 1H), 3.86 (s, 3H), 3.57 (s, 2H), 3.40 (s, 1H), 2.95 (s, 2H), 1.50 – 1.33 (m, 2H), 1.33 – 1.07 (m, 3H), 1.03 – 0.78 (m, 1H). HRMS *m/z* calcd for $C_{27}H_{26}N_5O_2$ 452.2081, Found 452.2715.
19b (27 mg, 59 %); ^{1}H NMR (400 MHz, $CDCl_3$) δ 8.28 (d,*J* = 4.6 Hz, 1H), 7.71 (d, *J* = 8.8 Hz, 1H), 7.24 (s, 1H), 7.10 (s, 1H), 7.01 (dd, *J* = 8.4, 2.0 Hz, 1H), 6.97 (dd, *J* = 8.8, 2.4 Hz, 1H), 6.84 (d, *J* = 8.4 Hz, 1H), 6.37 (s, 1H), 5.69 (s, 1H), 4.25 (d, *J* = 4.7 Hz, 2H), 4.24 (d, *J* = 4.7 Hz, 2H), 3.94 (m, 2H), 3.84 (s, 3H), 3.42 (m, 2H), 1.83 (m, 1H), 1.52 (m, 2H), 1.26 (m, 2H). HRMS *m/z* calcd for $C_{25}H_{26}N_5O_4$ 460.1979, Found 460.6220.
19c (36 mg, 70 %); ^{1}H NMR (400 MHz, $CDCl_3$) δ 8.24(d,*J* = 4.2 Hz, 1H), 7.89 (d, *J* = 1.3 Hz, 1H), 7.76 (d, *J* = 8.8 Hz, 1H), 7.66 (d, *J* = 2.2 Hz, 1H), 7.48 (d, *J* = 8.6 Hz, 1H), 7.41 (dd, *J* = 8.6, 1.3 Hz, 1H), 7.26 (s, 1H), 7.00 (dd, *J* = 8.8, 2.4 Hz, 1H), 6.79 (dd, *J* = 2.2, 0.8 Hz, 1H), 6.42 (s, 1H), 4.04 – 3.90 (s, 1H), 3.84 (s, 3H), 3.83 – 3.73 (m, 1H), 3.59 (m, 1H), 3.50 – 3.32 (m, 1H), 3.20 (m, 1H), 1.59 (m, 2H), 1.50 – 1.32 (m, 2H), 1.00 – 0.80 (m, 1H). ^{13}C NMR (101 MHz, MeOD) δ 162.59 (s), 147.65 (s), 146.26 (s), 145.04 (s), 143.51 (s), 139.79 (s), 138.39 (s), 120.07 (s), 117.28 (s), 116.86 (s), 114.65 (s), 111.13 (s), 107.32 (s), 105.74 (s), 104.19 (s), 103.30 (s), 98.43 (s), 91.83 (s), 90.58 (s), 50.00 (s), 40.37 (s), 40.03 (s), 28.57 (s), 15.77 (s).
HRMS *m/z* calcd for $C_{25}H_{24}N_5O_3$ 442.1874, Found 442.2824.

2.2. General syntheses of 2-Aryl-1-(2-((tetrahydro-2H-pyran-4-yl)amino)pyrimidin-4-yl)-1H-benzo[d]imidazol-5-ol (9a-9g)

2.2.1 2-(naphthalen-2-yl)-1-(2-((tetrahydro-2H-pyran-4-yl)amino)pyrimidin-4-yl)-1H-benzo[d]imidazol-5-ol (9a)

After dissolving compound 6a (24 mg, 0.053 mmol) in methylene chloride (0.5 ml), BBr3 (25 μl) was added slowly at -78 °C, and the reaction was stirred for 1 hour and then at room temperature for 2 hours. After confirming completion of the reaction, MeOH was added to quench the reaction, the organic solvent was removed *in vacuo*, and the residue was extracted with methylene chloride and washed with saturated $NaHCO_3$. The extracted organic layer was dried over anhydrous magnesium sulfate, filtered, concentrated, and purified by preparative chromatography (silica gel, methylene: MeOH = 20: 1) to obtain the title compound 9a, 20 mg, yield 86%. ^{1}H NMR (400 MHz, DMSO-d_6) δ 9.34 (1H, s), 8.45 (1H, s), 8.21 (1H, s), 7.92–8.00 (3H, m), 7.37–7.61 (5H, m), 7.13 (1H, d, *J* = 2.0 Hz), 6.86 (1H, dd, *J* = 8.8 Hz, *J* = 2.0 Hz), 4.78 (1H, brs), 3.00–3.03 (1H, m), 2.60–2.66 (1H, m), 1.96 (1H, d, *J* = 13.6 Hz), 1.75–1.78 (1H, m), 1.45 (1H, s), 1.23–1.34 (2H, m), 1.14 (2H, m); HRMS (ESI$^+$) calcd for $C_{26}H_{24}N_5O_2$ [M+H]+: 438.1925, found 438.4379.

2-(2,3-Dihydrobenzo[b][1,4]dioxin-6-yl)-1-(2-((tetrahydro-2H-pyran-4-yl)amino) pyrimidin-4-yl)-1H-benzo[d]imidazol-5-ol ***(9b)*** as a white solid, yield 36%; ^{1}H NMR (400 MHz, MeOD) δ 8.17 (d, *J* = 8.9 Hz, 1H), 8.07 (d, *J* = 7.2 Hz, 1H), 7.21 (d, *J* = 2.1 Hz, 1H), 7.15~7.08 (m, 2H), 6.99 (d, *J* = 8.4 Hz, 1H), 6.93 (dd, *J* = 8.9, 2.4 Hz, 1H), 6.33 (d, *J* = 7.2 Hz, 1H), 4.35 – 4.30 (m, 4H), 4.09-4.01 (s, 1H), 3.68 (m, 1H), 2.47 ~ 2.34 (m, 2H), 2.28 – 2.20 (m, 1H), 2.13 – 1.98 (m, 3H), 1.55 (m, 1H), 1.29 (m, 2H). HRMS (ES$^+$) calcd for $C_{26}H_{24}N_5O_4$ [M+H]+: 446.1823, found 446.3474.

2-(Benzofuran-5-yl)-1-(2-((tetrahydro-2H-pyran-4-yl)amino)pyrimidin-4-yl)-1H-benzo[d] imidazol-5-ol ***(9c)*** as a white solid, m.p. yield 93% ; ^{1}H NMR (400 MHz, CD_3OD) δ 8.27 (d, *J* = 1.6 Hz, 1H), 7.99 (dd, *J* = 8.7, 1.6 Hz, 1H), 77.84 (d, *J* = 4.5 Hz, 1H), 7.83 (d, *J* = 2.3 Hz, 1H), 7.61 (d, *J* = 8.7 Hz, 1H), 7.40 (d, *J* = 8.6 Hz, 1H), 6.98 – 6.93 (m, 2H), 6.78 (dd, *J* = 8.6, 2.3 Hz, 1H), 6.29 (d, *J* = 7.2 Hz, 1H), 4.17-4.04 (m, 2H), 3.96 (s, 2H), 3.84 – 3.77 (m, 1H), 3.72 (m, 2H), 2.24 (m, 1H), 1.98 –1.84 (m, 2H), 1.80 –1.70 (m, 1H). ; ^{13}C NMR (101 MHz, MeOD) δ 172.07 (s), 157.13 (s), 155.74 (s), 154.52 (s), 152.99 (s), 149.27 (s), 147.87 (s), 129.55 (s), 126.76 (s), 126.34 (s), 124.13 (s), 120.61 (s), 116.80 (s), 115.22 (s), 113.67 (s), 112.78 (s), 107.91 (s), 101.31 (s), 100.06 (s), 59.48 (s), 49.85 (s), 49.51 (s), 38.05 (s), 25.25 (s).;m.p. 93~95 °C; HRMS (ES$^+$) calcd for $C_{24}H_{22}N_5O_3$ [M+H]+: 428.1717, found 428.3226

2-(Naphthalen-2-yl)-1-(2-((tetrahydro-2H-pyran-4-yl)amino)pyrimidin-4-yl)-1H-benzo[d]imidazol-6-ol ***(22a)*** %); ^{1}H NMR (400 MHz, DMSO- d_6) δ 9.54 (s, 1H), 8.47 (s, 1H), 8.16 (s, 1H), 7.91–7.97 (m, 3H), 7.39–7.62 (m, 5H), 7.06 (s, 1H), 6.84–6.87 (m, 1H), 3.03–3.07 (m,1H), 2.63–2.67 (m,1H), 1.98 (d, *J* = 14.0 Hz, 1H), 1.72–1.75 (m, 1H), 1.46 (s,1H), 1.14–1.24 (m, 2H), 1.02–1.10 (m, 2H); ^{13}C NMR (101 MHz, DMSO) δ 161.75 (d, *J* = 3.4 Hz), 161.12 (s), 155.00 (s), 136.33 (s), 132.82 (s), 132.58 (s), 128.72 (s), 128.33 (s), 128.13 (s), 127.55 (s), 127.05 (d, *J* = 3.0 Hz), 126.68 (s), 125.92 (s), 125.13 (s), 120.16 (d, *J* = 5.5 Hz), 113.00 (s), 103.79 (s), 96.59 (s), 65.63 (d, *J* = 2.1 Hz), 31.89 (s), 29.00 (d, *J* = 1.2 Hz).; m.p. 247~249 °C; HRMS (ESI) calcd for $C_{26}H_{24}N_5O_2$ [M+H]+: 438.1925, found 438.3749.

2-(2,3-Dihydrobenzo[b][1,4]dioxin-6-yl)-1-(2-((tetrahydro-2H-pyran-4-yl)amino) pyrimidin-4-yl)-1H-benzo [d] imidazol-6-ol ***(22b)*** 81 %; ^{1}H NMR (400 MHz, CD_3OD) δ 8.37 (s, 1H), 7.53 (d, *J* = 8.4 Hz, 1H), 7.07 (s, 1H), 7.01 (s, 1H), 6.97 (dd, *J* = 8.4, 2.1 Hz, 1H), 6.90–6.85 (m, 2H), 6.64 (s, 1H), 4.28 (d, *J* = 5.0 Hz, 2H), 4.26 (d, *J* = 5.0 Hz, 2H), 3.90 (m, 2H), 3.62 (s, 1H), 3.39 (s, 1H), 1.29 (m, 4H), 0.91 (m, 3H).; ^{13}C NMR (101 MHz, DMSO) δ 161.80 (s), 161.23 (s), 156.33 (s), 155.33 (s), 154.34 (s), 148.13 (s), 136.03 (s), 135.32 (d, *J* = 9.0 Hz), 128.40 (s), 127.17 (s), 120.45 (s), 117.52 (d, *J* = 23.0 Hz), 113.35 (s), 103.47 (s), 96.40 (d, J = 23.6 Hz), 49.27 (s), 32.05 (d, J = 11.2 Hz), 25.17 (d, J = 6.1 Hz), 24.70 (s).; HRMS (ES$^+$) calcd for $C_{26}H_{24}N_5O_4$ [M+H]+: 446.1823, found 446.2844.

2-(Benzofuran-5-yl)-1-(2-((tetrahydro-2H-pyran-4-yl)amino)pyrimidin-4-yl)-1H-benzo[d]imidazol-6-ol ***(22c)*** 30 %; ^{1}H NMR (400 MHz, CD_3OD) δ 8.00 (d, *J* = 7.1 Hz, 1H), 7.97 (d, *J* = 1.5 Hz, 1H), 7.91 (d, *J* = 1.5Hz,1H), 7.69 (m,2H), 7.61-7.56 (m, 2H), 6.99-6.95 (m, 2H), 6.20 (d, *J* = 7.1 Hz, 1H), 4.28 (m, 2H), 3.99 (s, 1H), 3.86–3.74 (m, 2H), 2.43-2.34 (m, 1H), 2.14–2.02 (m, 2H), 1.92-1.72 (m, 2H), 1.28 (m, 1H). ^{13}C NMR (101 MHz, DMSO) δ 160.09 (s), 155.60 (s), 155.03 (s), 152.73 (s), 150.82 (s), 150.13 (s), 147.44 (s), 136.22 (s), 135.02 (s), 127.68 (s), 125.85 (s), 124.94 (s), 122.82 (s), 120.05 (s), 113.80 (s), 111.79 (s), 107.23 (s), 105.11 (s), 100.33 (s), 57.09 (s), 47.58 (s), 37.03 (d, *J* = 12.8 Hz), 22.81 (s).; m.p. 201~203 °C; HRMS (ES+) calcd for $C_{24}H_{22}N_5O_3$ [M+H]+: 428.1717, found 428.3226.

2.2.2. *N-cyclohexyl-4-(5-methoxy-2-(naphthalen-2-yl)-1H-benzo[d]imidazol-1-yl)pyrimidin-2-amine (7a)*

Compound **5a** (46 mg, 0.11 mmol) and cyclohexanamine (25 μl) were stirred in THF (1.1 ml) at 60° C. for 5 hours. After confirming the completion of the reaction, the reaction mixture was cooled to ambient temperature, the filtrate was distilled under reduced pressure, and the residue was purified by column chromatography (silica gel, n-hexane: ethyl acetate = 1: 1) to obtain the target compound **7a** (37 mg, 75%).; ^{1}H NMR (400 MHz, $CDCl_3$) δ8.22 (s,1H), 8.19 (d, *J* = 5.1 Hz, 1H), 7.86 – 7.77 (m, 3H), 7.77 – 7.68 (m, 1H), 7.58 – 7.47 (m, 3H), 7.36 (d, *J* = 2.5 Hz, 1H), 6.99 (dd, *J* = 8.9, 2.5 Hz, 1H), 6.47 – 5.97 (m, 1H), 5.43 (s, 1H), 3.89 (s, 3H), 3.32 (m, 1H), 1.66 (m, 2H), 1.43 (m, 3H), 1.30 – 1.15 (m, 2H), 1.09 – 0.90 (m, 3H). ; HRMS *m/z* calcd for $C_{28}H_{28}N_5O$ 450.2288, Found 450.3178.

7b (28 mg, 53 %);^{1}H NMR (400 MHz, $CDCl_3$)δ8.21(d,*J* = 5.3 Hz, 1H), 7.67 (d, *J* = 9.0 Hz, 1H), 7.33 (d, *J* = 1.8 Hz, 1H), 7.14 (s, 1H), 7.08 – 7.04 (m, 1H), 6.94 (dd, *J* = 9.0, 2.4 Hz, 1H), 6.87 (d, *J* = 8.4 Hz, 1H), 6.30 (s, 1H), 4.28 (d, *J* = 5.1 Hz, 2H), 4.25 (d, *J* = 5.1 Hz, 2H), 3.87 (s, 3H), 3.74 – 3.57 (m, 1H), 1.87 (m, 1H), 1.71 (m, 2H), 1.61 (m, 1H), 1.35 (m, 3H), 1.19 (m, 4H). HRMS *m*/*z* calcd for $C_{26}H_{28}N_5O_3$ 458.2187, Found 458.0696.

7c (35 mg, 53 %); ^{1}H NMR (400 MHz, CD_3OD) δ 8.27 (d, *J* = 1.6 Hz, 1H), 7.99 (dd, *J* = 8.7, 1.6 Hz, 1H), 77.84 (d, *J* = 4.5 Hz, 1H), 7.83 (d, *J* = 2.3 Hz, 1H), 7.61 (d, *J* = 8.7 Hz, 1H), 7.40 (d, *J* = 8.6 Hz, 1H), 6.98 – 6.93 (m, 2H), 6.78 (dd, *J* = 8.6, 2.3 Hz, 1H), 6.29 (d, *J* = 7.2 Hz, 1H), 4.17 – 4.04 (m, 2H), 3.96 (s, 2H), 3.84 – 3.77 (m, 1H), 3.72 (m, 2H), 2.24 (m, 1H), 1.98 – 1.84 (m, 2H), 1.80 – 1.70 (m, 1H). HRMS *m*/*z* calcd for $C_{26}H_{26}N_5O_2$ 440.2081, Found 440.9904.

7d (37 mg, 54 %); ^{1}H NMR (400 MHz, CDCl3) δ 8.30 (d, J = 5.3 Hz, 1H), 7.79 (s, 1H), 7.60 (d, J = 9.0 Hz, 1H), 7.44 (d, J = 8.3 Hz, 1H), 7.35 – 7.29 (m, 2H), 6.98 (dd, J = 9.0, 2.4 Hz, 1H), 6.56 – 6.19 (s, br, 1H), 6.17 – 5.61 (s, br, 1H), 3.88 (s, 3H), 1.77 – 1.56 (m, 5H), 1.26 – 1.01 (m, 6H). HRMS *m*/*z* calcd for $C_{24}H_{24}Cl_2N_5O$ 468.1352, Found 468.5313.

7e (34 mg, 65 %);^{1}H NMR (400 MHz, $CDCl_3$) δ 8.33 (d, *J* = 4.8 Hz, 1H), 7.97 (d, *J* = 5.3 Hz, 1H), 7.69 (s, 1H), 7.60 (d, *J* = 9.0 Hz, 1H), 7.33 (d, *J* = 2.0 Hz, 1H), 7.22 (m, 1H), 6.99 (dd, *J* = 7.0, 2.0 Hz, 1H), 6.49 (s, 1H), 3.89 (s, 3H), 3.36 (s, 1H), 2.03 (m, 1H), 1.67 (m, 4H), 1.58 (m, 1H), 1.31 (m, 1H), 1.15 (m, 4H). HRM S *m*/*z* calcd for $C_{25}H_{24}F_4N_5O$ 486.1911, Found 486.5868.

7f (35 mg, 67 %); ^{1}H NMR (400 MHz, $CDCl_3$)δ8.25(m,3H),7.82(d,*J* = 8.1 Hz, 1H), 7.74 (d, *J* = 8.1 Hz, 1H), 7.68 – 7.62 (m, 1H), 7.60 – 7.51 (m, 2H), 7.38 (d, *J* = 2.4 Hz, 1H), 7.02 (dd, *J* = 8.9, 2.4 Hz, 1H), 6.52 (s, 1H), 3.89 (s, 3H), 3.68 – 3.17 (s, 1H), 1.45 (m, 5H), 1.26 (m, 2H), 1.18 – 0.90 (m, 4H). HRMS *m*/*z* calcd for $C_{27}H_{27}N_6O$ $[M+H]^+$: 451.2241, Found 451.6728.

20a (29 mg, 69 %); ^{1}H NMR (400 MHz, $CDCl_3$) δ 8.21(s,1H), 8.16 (d, *J* = 5.3 Hz, 1H), 7.87 – 7.76 (m, 4H), 7.55 – 7.47 (m, 3H), 7.35 (d, *J* = 2.4 Hz, 1H), 7.02 (dd, *J* = 8.8, 2.4 Hz, 1H), 6.29 (s, 1H), 3.87 (s, 3H), 3.51 (s, 1H), 1.72 (m, 2H), 1.53 (m, 3H), 1.24 (m, 1H), 1.09 (m, 4H), 0.92 (m, 1H). HRMS *m*/*z* calcd for $C_{28}H_{28}N_5O$ 450.2288, Found 450.4124.

20b (40 mg, 71 %); ^{1}H NMR (400 MHz, $CDCl_3$) δ 8.22(d,*J* = 5.2 Hz, 1H), 7.69 (d, *J* = 8.8 Hz, 1H), 7.30 (d, *J* = 2.4 Hz, 1H), 7.12 (s, 1H), 7.02 (dd, *J* = 8.4, 2.1 Hz, 1H), 6.96 (dd, *J* = 8.8, 2.4 Hz, 1H), 6.84 (d, *J* = 8.4 Hz, 1H), 6.22 (s, 1H), 5.58 (s, 1H), 4.27 (dd, *J* = 3.6, 1.7 Hz, 2H), 4.24 (dd, *J* = 3.6, 1.7 Hz, 2H), 3.84 (s, 3H), 1.94 (m, 1H), 1.70 (m, 2H), 1.62 (m, 1H), 1.24 (m, 6H), 0.87 (m, 2H). HRMS *m*/*z* calcd for $C_{26}H_{28}N_5O_3$ 458.2187, Found 458.5738.

20c (34 mg, 57 %); ^{1}H NMR (400 MHz, $CDCl_3$)δ8.16(d,*J* = 5.1 Hz, 1H), 7.87 (s, 1H), 7.74 (d, *J* = 8.8 Hz, 1H), 7.66 (d, *J* = 2.2 Hz, 1H), 7.49 (d, *J* = 8.6 Hz, 1H), 7.44 (dd, *J* = 8.6, 1.6 Hz, 1Hs), 7.34 (d, *J* = 2.4 Hz, 1H), 7.00 (dd, *J* = 8.8, 2.4 Hz, 1H), 6.78 (dd, *J* = 2.2, 0.6 Hz, 1H), 6.21 (s, 1H), 3.86 (s, 3H), 3.71 – 3.52 (s, 1H), 1.84 (m, 2H), 1.67 (m, 2H), 1.59 (m, 1H), 1.48 – 1.28 (m, 1H), 1.17 (m, 4H), 1.02 – 0.77 (m, 1H). HRMS *m*/*z* calcd for $C_{26}H_{26}N_5O_2$ 440.2081, Found 440.6438.

20d (20 mg, 50 %); ^{1}H NMR (400 MHz, $CDCl_3$)δ8.33(d,*J* = 5.1 Hz, 1H), 7.79 (s, 1H), 7.73 (d, *J* = 8.8 Hz, 1H), 7.43 (d, *J* = 8.3 Hz, 1H), 7.29 (dd, *J* = 8.3, 2.0 Hz, 1H), 7.21 (d, *J* = 2.4 Hz, 1H), 7.00 (dd, *J* = 8.8, 2.4 Hz, 1H), 6.41 (s, 1H), 5.54 (s, 1H), 3.86 (s, 3H), 1.69 (m, 4H), 1.62 (m, 1H), 1.25 (m, 2H), 1.18 (s, 4H). HRMS *m*/*z* calcd for $C_{24}H_{24}Cl_2N_5O$ 468.1352, Found 468.6498.

20e (27 mg, 46 %); ^{1}H NMR (400 MHz, $CDCl_3$)δ8.32(s,1H),7.96(d,*J* = 5.1 Hz, 1H), 7.75 (s, 1H), 7.67 (s, 1H), 7.22 (m, 2H), 7.02 (dd, *J* = 8.8, 2.2 Hz, 1H), 6.43 (s, 1H), 3.86 (s, 3H), 3.39 (s, 1H), 2.03 (m, 1H), 1.69 (m, 3H), 1.59 (m, 1H), 1.22 (m, 6H). HRMS *m*/*z* calcd for $C_{25}H_{24}F_4N_5O$ 486.1911 Found 486.6498.

20f (31 mg, 98 %); ^{1}H NMR (400 MHz, $CDCl_3$)δ8.26(d,*J* = 4.0 Hz, 1H), 8.17 (s, 2H), 7.73 (dd, *J* = 8.3, 3.7 Hz, 2H), 7.64 (d, *J* = 7.2 Hz, 1H), 7.59 – 7.52 (m, 1H), 7.49 – 7.42 (m, 1H), 7.06 (s, 1H), 6.94 (dd, *J* = 8.8, 2.4 Hz, 1H), 6.44 (d, *J* = 4.0 Hz, 1H), 5.28 (s, 1H), 3.79 (s, 3H), 1.56 – 1.35 (m, 4H), 1.20 (m, 2H), 1.07 – 0.86 (m, 4H), 0.80 (m, 1H).). HRMS *m*/*z* calcd for $C_{27}H_{27}N_6O$ $[M+H]^+$: 451.2241, Found 451.2316.

2.2.3. 1-(2-(cyclohexylamino)pyrimidin-4-yl)-2-(naphthalen-2-yl)-1H-benzo[d]imidazol-5-ol (10a)

1-(2-(cyclohexylamino)pyrimidin-4-yl)-2-(naphthalen-2-yl)-1H-benzo[d]imidazol-5-ol 10a Compound 7a (37 mg, 0.082 mmol) was dissolved in methylene chloride (0.8 ml), BBr3 (39 μl) was added at -78 ^{O}C, and the reaction was stirred for 1 h and then at room temperature for 2 h. After the reaction was complete, MeOH was added to quench the reaction, the organic solvent was removed under reduced pressure, and the residue was extracted with methylene chloride and washed with saturated $NaHCO_3$ aqueous solution. The extracted organic layer was dried with anhydrous magnesium sulfate and filtered, the filtrate was concentrated under reduced pressure, and the residue was purified by column chromatography (silica gel, methylene chloride: MeOH = 20: 1) to give target compound **10a** (21 mg, 58%) was obtained. ^{1}H NMR (400 MHz, DMSO d_6) δ 9.35 (s, 1H), 8.40 (s, 1H), 8.21 (s, 1H), 7.95–8.00 (m, 2H), 7.52–7.80 (m, 4H), 7.29 (s, 1H), 7.14 (s, 1H), 6.87 (dd, *J* = 8.8 Hz, *J* = 2.4 Hz, 1H), 6.71 (s, 1H), 5.22 (brs, 1H), 2.90 (brs, 1H), 1.15–1.25 (m, 6H), 0.67–0.91 (m, 4H).; m.p. 239~241 °C; HRMS (ESI) calcd for $C_{27}H_{26}N_5O$ [M+H]+: 436.2132, found 436.1376.

1-(2-(Cyclohexylamino)pyrimidin-4-yl)-2-(2,3-dihydrobenzo[b][1,4]dioxin-6-yl)-1H-benzo [d]imidazol-5-ol ***(10b)*** 53 %; ^{1}H NMR (400 MHz, DMSO) δ 9.30 (s, 1H), 8.39 (m, 1H), 7.44 (s, 1H), 7.05 (s, 1H), 6.96 (s, 1H), 6.90 (d, *J* = 8.5 Hz, 1H), 6.78 (dd, *J* = 8.8, 2.3 Hz, 1H), 6.60 (s, 1H), 4.25 (s, 4H), 3.22 (s, 1H), 1.92 (m, 1H), 1.57 (m, 3H), 1.25 (m, 3H), 1.07 (m, 4H), 0.86 (m, 1H). ; ^{13}C NMR (101 MHz, DMSO) δ 161.96 (s), 157.02 (s), 154.11 (s), 144.59 (s), 143.76 (s), 143.11 (s), 128.52 (s), 126.56 (s), 123.93 (s), 122.17 (s), 117.49 (d, *J* = 5.2 Hz), 117.12 (d, *J* = 4.7 Hz), 112.99 (s), 103.99 (d, *J* = 19.2 Hz), 64.19 (d, *J* = 23.7 Hz), 49.43 (s), 32.30 (d, *J* = 11.9 Hz), 25.30 (d, *J* = 2.7 Hz), 24.89 (s).; m.p. 140~142 °C; HRMS (ESI) calcd for $C_{25}H_{26}N_5O_3$ [M+H]$^{+}$: 444.2030, found 444.3306.

2-(Benzofuran-5-yl)-1-(2-(cyclohexylamino)pyrimidin-4-yl)-1H-benzo[d]imidazol-5-ol ***(10c)*** 60 %; ^{1}H NMR (400 MHz, CD_3OD) δ 8.25 (s, 1H), 7.83 (s, 2H), 7.55 (d, *J* = 8.5 Hz, 2H), 7.42 (d, *J* = 8.3 Hz, 1H), 7.14 (d, *J* = 2.1 Hz, 1H), 6.96 – 6.85 (m, 2H), 6.53 (s, 1H), 4.16 – 3.53 (m, 1H), 3.15 (s, 1H), 1.51 (s, 5H), 1.26 (s, 1H), 1.18 – 0.82 (m, 5H).; ^{13}C NMR (101 MHz, MeOD) δ 163.38 (s), 161.36 (s), 159.05 (d, *J* = 9.8 Hz), 157.02 (s), 155.93 (s), 154.71 (s), 147.90 (s), 144.57 (s), 129.80 (s), 129.29 (s), 126.75 (s), 123.65 (s), 114.72 (s), 113.29 (s), 112.49 (s), 107.94 (s), 105.37 (s), 104.72 (s), 51.05 (s), 34.17 – 33.23 (m), 26.65 (s), 26.39 – 25.87 (m). HRMS (ESI) calcd for $C_{25}H_{24}N_5O_2$ [M+H]+: 426.1925, found 426.3058.

1-(2-(Cyclohexylamino)pyrimidin-4-yl)-2-(3,4-dichlorophenyl)-1H-benzo[d]imidazol-5-ol ***(10d)*** 54 %; H NMR (400 MHz, DMSO-d_6) d 9.34 (1H, s),8.10 (1H, d, *J* = 5.6 Hz), 7.82 (1H, d, *J* = 2.0 Hz), 7.70 (1H, d, *J* = 8.4 Hz), 7.44 (1H, dd, *J* = 8.4 Hz, *J* = 2.4 Hz), 7.19 (1H, d, *J* = 8.8 Hz), 7.09 (1H, d, *J* = 2.4 Hz), 6.83 (1H, dd, *J* = 8.8 Hz, *J* = 2.4 Hz), 6.71 (1H, d, *J* = 7.6 Hz), 6.45 (1H, dd, *J* = 5.6 Hz, *J* = 1.6 Hz), 6.38 (1H, d, *J* = 1.6 Hz), 3.61 (2H, s), 1.83–1.85 (2H, m), 1.66–1.70 (2H, m), 1.55–1.59 (1H, m),1.23–1.32 (3H, m), 1.10–1.19 (3H, m); HRMS(ESI) calcd for $C_{23}H_{22}Cl_2N_5O$ [M+H]+: 454.1196, found 454.3513.

1-(2-(Cyclohexylamino)pyrimidin-4-yl)-2-(4-fluoro-3-(trifluoromethyl)phenyl)-1H-benzo [d]imidazol-5-ol ***(10e)*** 74%;^{1}H NMR (400 MHz, CD_3OD) δ 8.40 (dd, *J* = 6.6, 2.0 Hz, 1H), 8.31 (m, 1H), 7.85 (m, 1H), 7.62 – 7.47 (m, 2H), 7.44 (d, *J* = 8.7 Hz, 1H), 6.97 (d, J = 2.0 Hz, 1H), 6.82 (dd, *J* = 8.7, 2.3 Hz, 1H), 3.72 (s, 1H), 1.98 – 1.93 (m, 1H), 1.76 (m, 2H), 1.70 – 1.49 (m, 4H), 1.18 – 1.08 (m, 2H), 0.91 – 0.82 (m, 3H). HRMS (ESI) calcd for $C_{24}H_4N_5O$ [M+H]+: 472.1755, found 472.3441.

1-(2-(Cyclohexylamino)pyrimidin-4-yl)-2-(quinolin-2-yl)-1H-benzo[d]imidazol-5-ol **(10f)** , 51 %; ^{1}H NMR (400 MHz, CD_3OD) δ 8.44 (t, *J* = 8.7 Hz, 1H), 8.37 (t, *J* = 5.5 Hz, 1H), 8.05 (s, 1H), 7.98 – 7.92 (m, 1H), 7.71 (dd, *J* = 9.9, 5.1 Hz, 2H), 7.68 – 7.54 (m, 2H), (d, *J* = 2.1 Hz, 1H), 7.00 – 6.93 (m, 1H), 6.74 (s, 1H), 3.35 (s, 1H), 2.85 (s, 1H), 1.57 – 1.34 (m, 3H), 1.32 – 1.09 (m, 3H), 1.07 – 0.72 (m, 5H).; ^{13}C NMR (101 MHz, DMSO) δ 161.93 (s), 155.70 (s), 154.35 (s), 146.52 (s), 143.64 (s), 136.79 (s), 136.09 (s), 130.09 (d, *J* = 6.1 Hz), 128.75 (d, *J* = 10.5 Hz), 127.92 (s), 127.33 (t, *J* = 9.9 Hz), 120.81 (s), 114.53 (s), 113.54 (s), 104.36 (s), 96.28 (s), 48.92 (s), 31.80 (d, *J* = 8.9 Hz), 25.12 (s), 24.61 (d, *J* = 4.3 Hz).; m.p. 157~159 °C ; HRMS (ESI) calcd for $C_{26}H_{25}N_6O$ [M+H]+: 437.2084, found 437.3665.

1-(2-(Cyclohexylamino)pyrimidin-4-yl)-2-(naphthalen-2-yl)-1H-benzo[d]imidazol-6-ol **(23a)** (10 mg, 38%); ^{1}H NMR (400 MHz, DMSO-d_6) δ 8.42 (s, 1H), 8.15 (s, 1H,), 7.93–7.95 (m, 4H), 7.28–7.62 (m, 5H), 7.07 (s, 1H), 6.85 (dd, *J* = 8.8 Hz, *J* = 2.4 Hz, 1H), 6.72 (s,1H), 2.89 (brs,1H), 1.23 (m, 6H), 0.66–0.85 (m,

4H) ;HRMS (ESI) calcd for $C_{27}H_{26}N_5O$ [M+H]+: 436.2132, found 436.3897.

1-(2-(Cyclohexylamino)pyrimidin-4-yl)-2-(2,3-dihydrobenzo[b][1,4]dioxin-6-yl)-1H-benzo [d]imidazol-6-ol **(23b)** 78 %); ^{1}H NMR (400 MHz, CD_3OD) δ 8.22 (s, 1H), 7.42 (d, *J* = 8.6 Hz, 1H), 6.98 (s, 1H), 6.91 (s, 1H), 6.86 (dd, *J* = 8.4, 2.1 Hz, 1H), 6.77 (dd, *J* = 8.6, 2.4 Hz, 2H), 6.46 (s, 1H), 4.17 (d, *J* = 5.1 Hz, 2H), 4.15 (d, *J* = 5.1 Hz, 2H), 3.34 (s, 1H), 2.20 – 1.78 (m, 1H), 1.61 (m, 3H), 1.53 (m, 1H), 1.18 (m, 2H), 1.14 – 0.74 (m, 4H). ; ^{13}C NMR (101 MHz, DMSO) δ 162.02 (s), 160.78 (s), 157.17 (s), 154.67 (s), 150.25 (s), 144.35 (s), 143.06 (s), 136.09 (s), 135.78 (s), 124.00 (s), 121.84 (s), 119.82 (s), 117.10 (d, J = 12.6 Hz), 112.70 (s), 104.03 (s), 96.61 (s), 64.14 (d, J = 20.8 Hz), 49.36 (s), 40.43 (s), 40.15 (s), 32.16 (s), 25.25 (s), 24.84 (s).; m.p. 176~178 °C; HRMS(ESI) calcd for $C_{25}H_{26}N_5O_3$ [M+H]+: 444.2030, found 444.0155.

2-(Benzofuran-5-yl)-1-(2-(cyclohexylamino)pyrimidin-4-yl)-1H-benzo[d]imidazol-6-ol **(23c,** 57 %); ^{1}H NMR (400 MHz, CD_3OD) δ 8.30 (d, *J* = 1.5 Hz, 1H), 7.85 (dd, *J* = 6.7, 2.2 Hz, 2H), 7.57 (d, *J* = 8.6 Hz, 2H), 7.43 (d, *J* = 8.6 Hz, 1H), 7.15 (s, 1H), 6.90 (dd, *J* = 10.3, 1.5 Hz, 2H), 6.64 (s, 1H), 3.95 – 3.81 (m, 1H), 3.56 (s, 1H), 2.00 (m, 2H), 1.57 (m, 5H), 0.96 – 0.81 (m, 4H). ; ^{13}C NMR (101 MHz, DMSO) δ 171.99 (s), 161.92 (s), 160.78 (s), 154.67 (d, *J* = 19.3 Hz), 146.98 (s), 136.23 (s), 135.69 (s), 127.37 (s), 126.16 (s), 125.28 (s), 121.93 (d, *J* = 16.7 Hz), 119.97 (s), 112.78 (s), 111.20 (s), 107.07 (s), 103.79 (s), 96.79 (s), 49.18 (s), 32.07 (d, *J* = 3.7 Hz), 25.15 (s), 24.63 (d, *J* = 2.1 Hz).; HRMS (ESI) calcd for $C_{25}H_{24}N_5O_2$ [M+H]+: 426.1925, found 426.3058.

1-(2-(Cyclohexylamino)pyrimidin-4-yl)-2-(3,4-dichlorophenyl)-1H-benzo[d]imidazol-6-ol **(23d,** 76 %); ^{1}H NMR (400 MHz, CD_3OD) δ 8.32 (s, 1H), 7.64 (s, 1H), 7.49 (dd, *J* = 8.5, 3.7 Hz, 2H), 7.28 (d, *J* = 8.5 Hz, 1H), 6.99 (s, 1H), 6.82 (dd, *J* = 8.7, 2.3 Hz, 1H), 6.68 (s, 1H), 3.13 – 2.89 (s, 1H), 1.67 – 1.39 (m, 6H), 1.04 (m, 5H), 0.79 (m, 1H). ; ^{13}C NMR (101 MHz, DMSO) δ 206.50 (s), 170.92 (s), 162.05 (s), 161.01 (s), 154.11 (s), 151.29 (s), 144.69 (s), 143.70 (s), 143.12 (s), 128.78 (s), 127.41 (s), 122.21 (s), 117.53 (s), 117.12 (s), 113.86 (s), 112.95 (s), 105.71 (s), 103.91 (s), 64.28 (s), 64.04 (s), 49.24 (s), 46.32 (s), 42.05 (s), 29.86 (s), 29.02 (s), 24.56 (s), 10.46 (s), 6.83 (s).; HRMS (ESI) calcd for $C_{23}H_{22}Cl_2N_5O$ $[M+H]^+$: 454.1196, found 454.4773.

1-(2-(Cyclohexylamino)pyrimidin-4-yl)-2-(4-fluoro-3-(trifluoromethyl)phenyl)-1H-benzo [d]imidazol-6-ol ***(23e,*** 78 %); ^{1}H NMR (400 MHz, DMSO) δ 9.64 (s, 1H), 8.53 – 8.45 (m, 1H), 7.83 (d, *J* = 5.5 Hz, 1H), 7.61 (dd, *J* = 13.7, 5.5 Hz, 2H), 7.48 (d, *J* = 7.9 Hz, 1H), 7.00 (s, 1H), 6.86 (dd, *J* = 9.0, 2.0 Hz, 1H), 2.93 (s, 1H), 1.79 (s, , 1H), 1.50 (m, 3H), 1.30 (m, 3H), 0.97 (m, 4H), 0.85 (m, 1H). ; ^{13}C NMR (101 MHz, DMSO) δ 161.53 (s), 160.00 (s), 159.39 (s), 155.75 (s), 150.89 (s), 146.57 (s), 137.23 (s), 136.88 (s), 135.92 (s), 130.13 (d, *J* = 1.6 Hz), 128.68 (s), 127.95 (s), 127.32 (d, *J* = 7.0 Hz), 125.93 (s), 123.86 (s), 120.76 (s), 113.64 (s), 96.34 (s), 48.61 (s), 31.81 (d, *J* = 7.6 Hz), 25.13 (s), 24.59 (d, *J* = 5.7 Hz)HRMS (ESI) calcd for $C_{24}H_{22}F_4N_5O$ $[M+H]^+$: 472.1755, found 472.3756.

1-(2-(Cyclohexylamino)pyrimidin-4-yl)-2-(quinolin-2-yl)-1H-benzo[d]imidazol-6-ol **(23f**, 78 %); ^{1}H NMR (400 MHz, DMSO) δ 8.52 (m, 2H), 8.18 (s, 1H), 8.03 (dd, *J* = 16.3, 7.8 Hz, 1H), 7.76 – 7.70 (m, 1H), 7.70 – 7.65 (m, 1H), 7.65 – 7.59 (m, 1H), 7.56 – 7.40 (m, 1H), 7.29 (s, 1H), 6.92 (dd, *J* = 25.0, 9.5 Hz, 2H), 2.87 (s, 1H), 1.99 – 1.82 (m, 1H), 1.70 (m, 1H), 1.26 (m, 4H), 1.07 (m, 2H), 0.84 (m, 4H). ;. ^{13}C NMR (101 MHz, DMSO) δ 161.53 (s), 160.00 (s), 159.39 (s), 155.75 (s), 150.89 (s), 146.57 (s), 137.23 (s), 136.88 (s), 135.92 (s), 130.13 (d, *J* = 1.6 Hz), 128.68 (s), 127.95 (s), 127.32 (d, *J* = 7.0 Hz), 125.93 (s), 123.86 (s), 120.76 (s), 113.64 (s), 96.34 (s), 48.61 (s), 31.81 (d, *J* = 7.6 Hz), 25.13 (s), 24.59 (d, *J* = 5.7 Hz).; m.p. 149~151 °C; HRMS (ESI) calcd fo r $C_{26}H_{25}N_6O$ $[M+H]^+$: 437.2084, found 437.2720.

2.2.4. (S)-tert-butyl 3-((4-(5-methoxy-2-(naphthalen-2-yl)-1H-benzo[d]imidazol-1-yl)pyrimidin-2-yl)amino)piperidine-1-carboxylate (8a)

Compound **5a** (218 mg, 0.506 mmol) and (*S*)-tert-butyl 3-aminopiperidine-1-carboxylate (199 µl) were stirred in THF (3.4 ml) at 60° C. for 12 hours. After confirming the completion of the reaction, the reaction mixture was cooled to ambient temperature, the filtrate was distilled under reduced pressure, and the residue was purified by column chromatography (silica gel, n-hexane: ethyl acetate = 1: 1) to obtain the target compound **8a** (138 mg, 50%); ^{1}H NMR (400 MHz, $CDCl_3$) δ 8.24(s,1H),8.18 (d,*J* = 5.0 Hz, 1H), 7.85 (dd, *J* = 18.9, 9.1 Hz, 3H), 7.78 (d, *J* = 7.4 Hz, 1H), 7.58 – 7.49 (m, 3H), 7.38 (d, *J* = 2.3 Hz, 1H), 7.01 (d, *J* = 8.6 Hz, 1H), 6.22 (d, *J* = 60.2 Hz, 1H), 3.90 (s, 3H), 3.75 – 3.50 (m, 2H), 3.39 (s, 1H), 3.31 – 3.13 (m, 2H), 2.16 – 1.89 (m, 1H), 1.43 (s, 9H), 1.32 – 1.22 (m, 3H), 0.97 – 0.79 (m, 1H). ; HRMS *m/z* calcd

for $C_{32}H_{35}N_6O_3$ [M+H]$^+$: 551.2765, Found 551.2755.

8b (142 mg, 74 %) ; ^{1}H NMR (400 MHz, $CDCl_3$)δ8.23(d, *J* = 5.0 Hz, 1H), 7.69 (d, *J* = 8.5 Hz, 1H), 7.31 (s, 1H), 7.14 (s, 1H), 7.04 (d, *J* = 7.9 Hz, 1H), 6.96 (d, *J* = 2.0 Hz, 1H), 6.85 (d, *J* = 8.4 Hz, 1H), 6.23 (s, 1H), 4.28 (d, *J* = 4.9 Hz, 2H), 4.24 (d, *J* = 4.9 Hz, 2H), 3.86 (s, 3H), 3.78 (s, 1H), 3.45 (m, 2H), 3.37 (m, 1H), 1.66 (m, 2H), 1.53 (m, 2H), 1.43 (s, 9H), 1.23 (m, 2H). HRMS *m/z* calcd for $C_{30}H_{35}N_6O_5$ [M+H]$^+$: 559.2663, Found 559.3417.

8c (mg, %); ^{1}H NMR (400 MHz, CDCl3) δ 8.12 (m, 2H), 7.71 (dd, J = 7.2, 3.4 Hz, 2H), 7.61 – 7.48 (m, 3H), 7.03 (m, 1H), 6.87 (d, J = 1.6 Hz, 1H), 6.29 (s, 1H), 3.91 (s, 3H), 3.85 – 3.42 (m, 3H), 3.27 (s, 2H), 1.73 (m, 3H), 1.44 (m, 9H), 1.12 – 0.78 (m, 1H). HRMS *m/z* calcd for $C_{30}H_{37}N_6O_5$ [M+H]$^+$: 561.2820, Found 561. 2701.

8d (121 mg, 73 %) ; ^{1}H NMR (400 MHz, $CDCl_3$)δ8.27(d,*J* = 5.2 Hz, 1H), 7.77 (d, *J* = 2.0 Hz, 1H), 7.60 (d, *J* = 8.8 Hz, 1H), 7.39 (d, *J* = 8.3 Hz, 1H), 7.27 – 7.24 (m, 2H), 6.94 (d, *J* = 2.5 Hz, 1H), 6.40 – 6.14 (m, 1H), 3.83 (s, 3H), 3.68 – 3.52 (s, 1H), 3.45 (m, 1H), 3.30 (s, 1H), 3.25 (m, 2H), 1.63 (m, 2H), 1.41 (s, 9H), 1.32 – 1.23 (m, 2H). HRMS *m/z* calcd for $C_{28}H_{31}N_6O_3$ [M+H]$^+$: 569.1829, Found 569.2983.

8e (143 mg, 73 %);^{1}H NMR (400 MHz, $CDCl_3$)δ8.31(s,1H),7.95(d,*J* = 9.0 Hz, 1H), 7.69 (s, 1H), 7.63 (s, 1H), 7.31 (d, *J* = 2.1 Hz, 1H), 7.22 (m, 1H), 7.06 – 6.95 (m, 1H), 6.32 (s, 1H), 3.87 (s, 3H), 3.63 (m, 1H), 3.45 (s, 1H), 3.26 (m, 2H), 2.01 – 1.81 (m, 1H), 1.68 (m, 2H), 1.52 – 1.34 (s, 9H), 1.25 (m, 2H), 0.85 (m, 1H). HRMS *m/z* calcd for $C_{29}H_{31}F_4N_6O_3$ [M+H]$^+$: 587.69.1829, Found 587.6990.

8f (100 mg, 50%); ^{1}H NMR (400 MHz, $CDCl_3$)δ8.28(m,*J* = 8.4 Hz, 3H), 7.82 (d, *J* = 8.0 Hz, 1H), 7.70 (s, 1H), 7.66 (d, *J* = 7.5 Hz, 1H), 7.64 – 7.52 (m, 2H), 7.37 (d, *J* = 2.3 Hz, 1H), 7.03 (d, *J* = 8.0 Hz, 1H), 6.49 (d, *J* = 5.4 Hz, 1H), 3.89 (s, 3H), 3.70 (s, 1H), 3.60 – 3.31 (m, 2H), 3.15 (m, 2H), 1.52 (m, 2H), 1.50 – 1.43 (m, 3H), 1.42 – 1.26 (s, 9H). HRMS *m/z* calcd for $C_{31}H_{34}N_7O_3$ [M+H]$^+$: 552.2718, Found 552.3784.

8g (69 mg, 50 %) ; ^{1}H NMR (400 MHz, CD_3OD) δ 8.40 (s, 1H), 7.61 (s, 1H), 7.30 (s, 1H), 7.00 (m, 3H), 6.87 (s, 1H), 6.45 (s, 1H), 6.02 (s, 2H), 4.08 (s, 1H), 3.85 (s, 3H), 3.62 (s, 1H), 3.35 (m, 1H), 2.99 (m, 2H), 2.00 (m, 2H), 1.71 (m, , 3H), 1.23 (m, 1H). HRMS *m/z* calcd for $C_{29}H_{33}N_6O_5$ [M+H]$^+$: 545.2507, Found 545.7300.

21a (180 mg, 57 %); ^{1}H NMR (400 MHz, $CDCl_3$) δ 8.19 (d, *J* = 5.2 Hz, 2H), 7.85 (m, 2H), 7.79 (m, 2H), 7.56 – 7.47 (m, 3H), 7.36 (s, 1H), 7.01 (dd, *J* = 8.8, 2.4 Hz, 1H), 6.29 (s, 1H), 3.86 (s, 3H), 3.78 – 3.57 (s, 1H), 3.32 (m, 3H), 1.76 – 1.52 (m, 2H), 1.43 (s, 9H), 1.28 (m, 1H), 1.25 (d, *J* = 7.1 Hz, 2H), 1.17 – 0.79 (m, 1H). HRMS *m/z* calcd for $C_{32}H_{35}N_6O_3$ [M+H]$^+$: 551.2765, Found 551.2755.

21b (159 mg, 66 %); ^{1}H NMR (400 MHz, $CDCl_3$)δ8.20(d,*J* = 5.2 Hz, 1H), 7.64 (d, *J* = 8.8 Hz, 1H), 7.29 (s, 1H), 7.08 (s, 1H), 6.96 (dd, *J* = 8.4, 1.7 Hz, 1H), 6.91 (dd, *J* = 8.8, 2.2 Hz, 1H), 6.80 (d, *J* = 8.4 Hz, 1H), 6.19 (s, 1H), 5.88 (s, 1H), 4.21 (d, *J* = 6.5 Hz, 2H), 4.17 (d, *J* = 6.5 Hz, 2H), 3.78 (s, 3H), 3.45 (m, 1H), 3.20 (m, 2H), 1.67 (m, 1H), 1.47 (m, 3H), 1.35 (s, 9H), 1.20 (m, 2H). HRMS *m/z* calcd for $C_{30}H_{35}N_6O_5$ [M+H]$^+$: 559.2663, Found 559.3102.

21c (90 mg, 52 %); ^{1}H NMR (400 MHz, $CDCl_3$)δ8.21(s,1H),7.98(s,1H),7.86(d,*J* = 8.8 Hz, 1H), 7.69 (d, *J* = 1.7 Hz, 1H), 7.53 (d, *J* = 8.0 Hz, 1H), 7.47 (d, *J* = 8.0 Hz, 1H), 7.33 (s, 1H), 7.06 (d, *J* = 8.8 Hz, 1H), 6.84 (d, *J* = 1.7 Hz, 1H), 6.26 (s, 1H), 3.86 (s, 3H), 3.70 (s, 1H), 3.46 (s, 1H), 3.31 (m, 2H), 1.70 (m, 2H), 1.48 (m, 2H), 1.45 (m, 2H), 1.42 (s, 9H). HRMS *m/z* calcd for for $C_{30}H_{37}N_6O_5$ [M+H]$^+$: 561.2820, found 561.4378.

21d (210 mg, 55 %); ^{1}H NMR (400 MHz, $CDCl_3$)δ8.32(d,*J* = 4.8 Hz, 1H), 7.78 (s, 1H), 7.70 (d, *J* = 8.8 Hz, 1H), 7.41 (d, *J* = 8.3 Hz, 1H), 7.26 (dd, *J* = 8.3, 1.9 Hz, 1H), 7.21 (s, 1H), 6.98 (dd, *J* = 8.8, 2.4 Hz, 1H), 6.36 (s, 1H), 5.78 (s, 1H), 3.83 (s, 3H), 3.65 (m, 1H), 3.43 (m, 1H), 3.25 (sm, 2H), 1.76 – 1.61 (m, 2H), 1.42 (s, 9H), 1.25 (m, 2H), 1.24 (m, 1H). HRMS *m/z* calcd for $C_{28}H_{31}N_6O_3$ [M+H]$^+$: 569.1829, Found 569.1723.

21e (93 mg, 50 %); ^{1}H NMR (400 MHz, $CDCl_3$) δ 8.34 (d, *J* = 4.1 Hz, 1H), 7.93 (d, *J* = 4.1 Hz, 1H), 7.70 (d, *J* = 8.8 Hz, 1H), 7.62 (s, 1H), 7.17 (m, 2H), 6.97 (dd, *J* = 8.8, 2.4 Hz, 1H), 6.37 (s, 1H), 5.78 (s, 1H), 3.82 (s, 3H), 3.60 (m, 1H), 3.41 (m, 1H), 3.22 (m, 2H), 1.65 (m, 2H), 1.42 (m, 3H), 1.39 (s, 9H). HRMS *m/z* calcd for $C_{29}H_{31}F_4N_6O_3$ [M+H]$^+$: 587.69.1829, Found 587.2894.

21f (100 mg, 50 %); ^{1}H NMR (400 MHz, $CDCl_3$) δ 8.26 (d, J = 4.0 Hz, 1H), 8.17 (s, 2H), 7.73 (dd, J = 8.3,

3.7 Hz, 2H), 7.64 (d, J = 7.2 Hz, 1H), 7.59 – 7.52 (m, 1H), 7.49 – 7.42 (m, 1H), 7.06 (s, 1H), 6.94 (dd, J = 8.8, 2.4 Hz, 1H), 6.44 (d, J = 4.0 Hz, 1H), 5.28 (s, 1H), 3.79 (s, 3H), 1.56 – 1.35 (m, 4H), 1.20 (m, 2H), 1.07 – 0.86 (m, 4H), 0.80 (m, 1H). HRMS *m/z* calcd for $C_{31}H_{34}N_7O_3$ [M+H]$^+$: 552.2718, Found 552.3469.
21g (190 mg, 58 %); ^{1}H NMR (400 MHz, $CDCl_3$)δ8.21(d,*J* = 5.2 Hz, 1H), 7.63 (d, *J* = 8.8 Hz, 1H), 7.26 (d, *J* = 2.9 Hz, 1H), 7.02 – 6.98 (m, 2H), 6.91 (dd, *J* = 8.8, 2.9 Hz, 1H), 6.76 (d, *J* = 8.4 Hz, 1H), 6.19 (s, 1H), 5.95 (s, 2H), 3.78 (s, 3H), 3.44 (s, 1H), 3.20 (m, 2H), 1.83 (m, 1H), 1.68 (m, 2H), 1.53 – 1.45 (m, 2H), 1.45 – 1.31 (s, 9H), 1.21 (m, , 2H). HRMS *m/z* calcd for $C_{29}H_{33}N_6O_5$ [M+H]$^+$: 545.2507, Found 545.0368.

2.2.5. (S)-4-(5-methoxy-2-(naphthalen-2-yl)-1H-benzo[d]imidazol-1-yl)-N-(piperidin-3-yl)pyrimidin-2-amine (11a)

Compound **8a** (377 mg, 0.61 mmol) was dissolved in 1, 4-dioxane (6.1 ml) and treated with 1,4- dioxane in 4 M-HCl (3 ml) at room temperature. The reaction mixture was stirred at room temperature for 20 minutes. The mixture was diluted with ether and stirred until the product separated into a solid. The solid product was filtered and washed with ether followed by hexane. The crude product was then crystallized to obtain the title compound **11a** (290 mg, 65%).; ^{1}H NMR (400 MHz, DMSO) δ 9.48 (s, 1H), 7.97 (d, *J* = 5.2 Hz, 3H), 7.72 (m, 2H), 7.64 – 7.53 (m, 3H), 7.35 (s, 1H), 7.00 (s, 1H), 6.64 (s, 1H), 3.85 (s, 3H), 3.37 (s, 1H), 3.17 – 2.88 (m, 2H), 2.86 – 2.57 (m, 2H), 1.89 (m, 2H), 1.20 (m, 3H). ; HRMS *m/z* calcd for $C_{16}H_{13}N_5O_4S$ 371.3710, Found 372.4070 (M+H$^+$). HRMS *m/z* calcd for $C_{27}H_{27}N_6O$ [M+H]$^+$: 451.2241 , Found 451.2632.

11b (54 mg, 73 %); ^{1}H NMR (400 MHz, CD_3OD) δ 8.32 (s, 1H), 7.52 (s, 1H), 7.22 (s, 1H), 6.99 (s, 1H), 6.90 (d, *J* = 8.5 Hz, 2H), 6.80 (d, *J* = 8.5 Hz, 1H), 6.46 (s, 1H), 4.22 (d, *J* = 4.8 Hz, 2H), 4.20 (d, *J* = 4.8 Hz, 2H), 3.99 (s, 1H), 3.81 (s, 3H), 3.57 (s, 1H), 3.24 (m, 1H), 3.03 – 2.85 (m, 2H), 1.97 (m, 2H), 1.66 (m, 3H), 1.22 (m, 1H). HRMS *m/z* calcd for $C_{25}H_{27}N_6O_3$ [M+H]$^+$: 459.2139, Found 459.3300.

11c (10 mg, 61%); ^{1}H NMR (400 MHz, MeOD) δ 8.55 (s, 1H), 8.12 (s, 1H), 8.01 (d, *J* = 2.0 Hz, 1H), 7.91 (s, 1H), 7.80 (d, *J* = 8.3 Hz, 1H), 7.64 (d, *J* = 8.3 Hz, 1H), 7.41 (s, 1H), 7.33 (d, *J* = 8.1 Hz, 1H), 7.07 (s, 1H), 6.75 (s, 1H), 4.31 (s, 1H), 3.98 (m, 3H), 3.72–3.51 (m,1H), 3.28 – 3.18 (m, 1H), 2.98 (m, 2H), 2.02 (s, 1H), 1.86 (m, 1H), 1.73 (m, 3H), 1.29 (m, 1H); HRMS *m/z* calcd for $C_{25}H_{25}N_6O_2$ [M+H]$^+$: 441.2034, Found 441.46 31.
11d (71 mg, 83 %); ^{1}H NMR (400 MHz, CD_3OD) δ 8.46 (s, 1H), 7.77 (d, *J* = 1.8 Hz, 1H), 7.61 (d, *J* = 8.3 Hz, 2H), 7.45 – 7.40 (m, 1H), 7.27 (d, *J* = 1.4 Hz, 1H), 7.02 (d, *J* = 8.3 Hz, 1H), 6.69 (s, 1H), 3.86 (s, 3H), 3.35 (s, 1H), 3.15 – 2.85 (m, 3H), 2.14 – 1.90 (m, 2H), 1.65 (m, 4H), 1.20 (m, 1H). HRMS *m/z* calcd for $C_{23}H_{23}Cl_2N_6O$ [M+H]$^+$: 469.1305, Found 469.1930.
11e (50 mg, 67 %); ^{1}H NMR (400 MHz, CD_3OD) δ 8.46 (d, *J* = 4.8 Hz, 1H), 7.92 (d, *J* = 5.7 Hz, 1H), 7.85 (s, 1H), 7.63 (s, 1H), 7.47 (m, 1H), 7.25 (d, *J* = 2.2 Hz, 1H), 7.03 (d, *J* = 8.2 Hz, 1H), 6.65 (s, 1H), 3.95 (s, 1H), 3.87 (s, 3H), 3.26 (s, 1H), 3.01 – 2.84 (m, 2H), 2.19 – 1.91 (m, 2H), 1.91 – 1.53 (m, 3H), 1.53 – 1.22 (m, 1H), 1.21 – 0.47 (m, 1H). HRMS *m/z* calcd for $C_{24}H_{23}F_4N_6$ [M+H]$^+$: 487.1864, Found 487.3115.
11f (47 mg, 61%); ^{1}H NMR (400 MHz, CD_3OD) δ 8.47 (d, *J* = 7.8 Hz, 2H), 8.08 (s, 1H), 7.97 (d, *J* = 8.0 Hz, 1H), 7.72 (s, 2H), 7.62 (s, 1H), 7.56 (d, *J* = 7.5 Hz, 1H), 7.33 (s, 1H), 7.06 (d, *J* = 8.9 Hz, 1H), 6.76 (s, 1H), 3.87 (s, 3H), 3.84 (s, 1H), 3.62 (s, 1H), 3.19 (s, 1H), 3.06 (m, 1H), 2.88 (m, 1H), 2.74 (m, 1H), 1.81 (m, 2H), 1.63 – 1.25 (m, 3H). HRMS *m/z* calcd for $C_{26}H_{26}N_7O$ [M+H]$^+$: 452.2193, Found 452.3661.
11g (41 mg, 92%); ^{1}H NMR (400 MHz, CD_3OD) δ 8.40 (s, 1H), 7.61 (s, 1H), 7.30 (s, 1H), 7.00 (m, 3H), 6.87 (s, 1H), 6.45 (s, 1H), 6.02 (s, 2H), 4.08 (s, 1H), 3.85 (s, 3H), 3.62 (s, 1H), 3.35 (m, 1H), 2.99 (m, 2H), 2.00 (m, 2H), 1.71 (m, , 3H), 1.23 (m, 1H). HRMS *m/z* calcd for $C_{24}H_{25}N_6O_3$ [M+H]$^+$: 445.1983, Found 445.3390.
24a (52 mg, 67 %); ^{1}H NMR (400 MHz, DMSO) δ 8.41 (s, 1H), 8.17 (s, 1H), 7.99 – 7.92 (m, 3H), 7.79 (d, *J* = 26.2 Hz, 1H), 7.71 (d, *J* = 8.8 Hz, 1H), 7.57 (m, 3H), 7.33 (m, 1H), 7.00 (dd, *J* = 8.8, 2.4 Hz, 1H), 6.51 (s, 1H), 3.84 (s, 3H), 3.69 (s, 1H), 3.43 – 3.20 (m, 1H), 2.96 (m, 2H), 2.81 – 2.53 (m, 2H), 1.99 – 1.45 (m, 2H), 1.41 – 1.04 (m, 3H). HRMS *m/z* calcd for $C_{27}H_{26}N_6O$ [M+H]$^+$: 450.2168, Found 450.1288.

24b (110 mg, 90 %); ^{1}H NMR (400 MHz, DMSO) δ 8.13 (s, 1H), 7.35 (d, *J* = 8.7 Hz, 1H), 6.87 (s, 1H), 6.69

(m, 3H), 6.57 (d, *J* = 8.7 Hz, 1H), 6.31 (s, 1H), 4.02 – 3.86 (m, 4H), 3.69 (s, 1H), 3.54 (s, 3H), 3.04 (s, 1H), 3.03 – 2.96 (m, 2H), 2.78 – 2.54 (m, 2H), 1.71 (m, 2H), 1.41 (m, 2H), 1.12 – 0.53 (m, 1H). HRMS *m/z* calcd for $C_{25}H_{27}N_6O_3$ [M+H]$^+$: 459.2139, Found 459.3300.

24c (30 mg, 43 %); ^{1}H NMR (400 MHz, CD_3OD) δ 8.79 (s, 1H), 8.25 (d, *J* = 2.1 Hz, 1H), 8.23 (s, 1H), 8.07 (d, *J* = 8.8 Hz, 1H), 7.96 (d, *J* = 8.5 Hz, 1H), 7.84 (d, *J* = 8.5 Hz, 1H), 7.63 (s, 1H), 7.40 (dd, *J* = 8.8, 2.3 Hz, 1H), 7.30 (d, *J* = 1.6 Hz, 1H), 7.08 (s, 1H), 4.25 (s, 3H), 4.04 (s, 1H), 3.65 (s, 1H), 3.38 (m, 2H), 2.41 (m, 1H), 2.25 (m, 1H), 1.89 (m, 3H), 1.67 (m, 1H), 1.58 – 1.10 (m, 1H). ; HRMS *m/z* calcd for $C_{25}H_{25}N_6O_2$ [M+H]$^+$: 441.2034, Found 441.3390.

24d (110 mg, 62 %); ^{1}H NMR (400 MHz, DMSO) δ 9.69 (s, 1H), 9.41 (m, 3H), 8.58 – 8.46 (m, 1H), 8.26 (m, 1H), 7.90 (s, 1H), 7.74 (m, 2H), 7.50 (dd, *J* = 8.4, 2.1 Hz, 1H), 7.08 (dd, *J* = 8.9, 2.1 Hz, 1H), 6.84 – 6.50 (m, 1H), 3.97 (m, 1H), 3.84 (s, 3H), 3.55 (s, 1H), 3.45 – 3.28 (m, 1H), 3.15 (s, 1H), 3.09 (s, 1H), 2.80 (m, 2H), 1.84 (m, 2H), 1.47 (m, 2H). HRMS *m/z* calcd for $C_{23}H_{23}Cl_2N_6O$ [M+H]$^+$: 469.1305, Found 469.2560.

24e (57 mg, 80 %); ^{1}H NMR (400 MHz, CD_3OD) δ 8.50 (s, 1H), 7.88 (d, *J* = 5.7 Hz, 1H), 7.82 (s, 1H), 7.63 (d, *J* = 8.8 Hz, 1H), 7.45 (m, 1H), 7.18 (s, 1H), 7.01 (dd, *J* = 8.8, 2.3 Hz, 1H), 6.73 (s, 1H), 3.84 (s, 3H), 3.35 (s, 1H), 3.13 (m, 2H), 2.87 (m, 2H), 2.18 – 1.86 (m, 2H), 1.63 (m, 3H), 1.26 (m, 1H).; HRMS *m/z* calcd fo r $C_{24}H_{23}F_4N_6$ [M+H]$^+$: 487.1864, Found 487.2800.

24f (43 mg, 81 %); ^{1}H NMR (400 MHz, CDCl3) δ 8.33 (d, J = 6.7 Hz, 2H), 7.76 (d, J = 8.0 Hz, 2H), 7.62 (d, J = 8.4 Hz, 1H), 7.56 (m, 2H), 7.43 (m, 1H), 7.02 (s, 1H), 6.97 (d, J = 8.9 Hz, 1H), 6.78 (s, 1H), 4.15 – 3.80 (s, 1H), 3.60 (s, 3H), 3.28 – 3.22 (m, 1H), 3.20 – 2.99 (m, 1H), 2.96 (m, 1H), 2.94 – 2.75 (m, 1H), 2.62 (m, 2H), 1.87 – 1.60 (m, 1H), 1.39 (m, 1H), 1.24 (m, 2H). HRMS *m/z* calcd for $C_{26}H_{26}N_7O$ [M+H]$^+$: 452.2193, Found 452.3030.

24g (118 mg, 77 %); ^{1}H NMR (400 MHz, CD_3OD) δ 8.41 (s, 1H), 7.59 (d, *J* = 8.8 Hz, 1H), 7.17 (s, 1H), 6.99 (dd, *J* = 8.1, 1.5 Hz, 1H), 6.98 – 6.95 (m, 2H), 6.86 (d, *J* = 8.1 Hz, 1H), 6.62 (s, 1H), 6.02 (d, *J* = 1.0 Hz, 1H), 6.01 (d, *J* = 1.0 Hz, 1H), 4.00 (s, 1H), 3.81 (s, 3H), 3.35 (s, 1H), 3.26 (m 1H), 3.05 – 2.86 (m, 2H), 1.99 (m, 2H), 1.70 (m, 3H), 1.26 (m, 1H). HRMS *m/z* calcd for $C_{24}H_{25}N_6O_3$ [M+H]$^+$: 445.1983, Found 445.2760.

2.2.6. (S)-cyclopropyl(3-((4-(5-methoxy-2-(naphthalen-2-yl)-1H-benzo[d]imidazol-1-yl)pyrimidin-2-yl)amino)piperidin-1-yl)methanone (12a)

Compound **11a** (100 mg, 0.22 mmol) was cooled to 0 °C in THF (0.55 ml) and treated with TEA (46 μL). To the mixture, cyclopropanecarbonyl chloride (23 mg) was added at 0 °C, and the mixture was raised to room temperature and stirred for 1 hour. The reaction mixture was concentrated in vacuo, diluted with methylene chloride, and washed with water and a saturated aqueous sodium chloride solution. The organic layer was dried over sodium sulfate, filtered, and the filtrate was distilled under reduced pressure, and the residue was purified by column chromatography (silica gel, DCM: MEOH 40: 1) to obtain the title compound **12a** (73 mg, 64%).; ^{1}H NMR (400 MHz, $CDCl_3$) δ 8.18 (s, 2H), 7.81 (m, 4H), 7.57–7.47 (m, 3H), 7.32 (s, 1H), 7.01 (dd, *J* = 8.8, 2.2 Hz, 1H), 6.36 (m, 1H), 4.04 (s, 1H), 3.86 (s, 3H), 3.74 (m, 1H), 3.46 (m, 2H), 3.10 (m, 1H), 1.59 (m, 4H), 1.33 – 1.12 (m, 1H), 1.05 – 0.70 (m, 4H), 0.44 (s, 1H).; HRMS *m/z* calcd for $C_{31}H_{31}N_6O_2$ [M+H]$^+$: 519.2503, Found 519.2940.

12b (48 mg, 77 %); ^{1}H NMR (400 MHz, CD_3OD) δ 8.32 (d, *J* = 33.2 Hz, 1H), 7.70 – 7.44 (m, 1H), 7.21 (s, 1H), 7.04 (s, 1H), 6.94 (m, 2H), 6.86 (d, *J* = 8.4 Hz, 1H), 6.48 (d, *J* = 33.2 Hz, 1H), 4.25 (d, *J* = 6.6 Hz, 4H), 3.97 (s, 1H), 3.85 (s, 3H), 3.52 (m, 1H), 3.31 (m, 1H), 3.03 – 2.85 (m, 1H), 1.95 (m, 1H), 1.77 – 1.69 (m, 2H), 1.59 – 1.52 (m, 1H), 1.31 (m, 1H), 0.88 (m, 3H), 0.84 – 0.68 (m, 2H). ; HRMS *m/z* calcd for $C_{29}H_{31}N_6O_4$ [M+H]$^+$: 527.2401, Found 527.7069.

12c (5 mg, 55 %): ^{1}H NMR (400 MHz, MeOD) δ 8.32 (m, 1H), 7.86 (d, *J* = 2.2 Hz, 2H), 7.74 (d, *J* = 8.5 Hz, 1H), 7.58 (d, *J* = 8.5 Hz, 1H), 7.46 (s, 1H), 7.27 (s, 1H), 7.00 (d, *J* = 8.8 Hz, 1H), 6.92 (s, 1H), 6.65 (s, 1H), 4.14 (s, 1H), 3.98 (m, 1H), 2.05 – 1.91 (m, 2H), 1.59 – 1.52 (m, 3H), 1.33 – 1.23 (m, 4H), 0.90 – 0.85 (m, 5H). HRMS *m/z* calcd for $C_{29}H_{29}N_6O_3$ [M+H]$^+$: 509.2296, Found 509.5260.

12d (60 mg, 78 %): ^{1}H NMR (400 MHz, $CDCl_3$)δ8.27(s,1H),7.76(d,*J* = 22.0 Hz, 1H), 7.60 (d, *J* = 30.5 Hz,

1H), 7.44 (d, *J* = 8.3 Hz, 1H), 7.30 (d, *J* = 2.2 Hz, 2H), 6.97 (s, 1H), 6.47 – 6.16 (m, 1H), 4.01 (s, 1H), 3.86 (s, 3H), 3.46 (m, 1H), 3.18 (m, 1H), 1.74 (m, 2H), 1.61 – 1.52 (m, 2H), 1.52 – 1.44 (m, 1H), 1.40 (m, 1H), 1.23 (m, 1H), 0.98 – 0.92 (m, 1H), 0.89 – 0.71 (m, 4H). ;HRMS *m/z* calcd for $C_{27}H_{27}Cl_2N_6O_2$ $[M+H]^+$: 537.1567, Found 537.2227.

12e (32 mg, 61 %): ^{1}H NMR (400 MHz, CD_3OD) δ 8.50 – 8.39 (m, 1H), 7.95 (s, 1H), 7.81 (s, 1H), 7.65 (m, 1H), 7.44 (m, 1H), 7.27 (s, 1H), 7.02 (d, *J* = 8.6 Hz, 1H), 6.76 (s, 1H), 4.12 – 4.01 (s, 1H), 3.88 (s, 3H), 3.27 – 3.14 (m, 1H), 3.07 – 2.93 (m, 1H), 2.82 (s, 1H), 1.93 (m, 1H), 1.81 – 1.69 (m, 2H), 1.62 – 1.51 (m, 2H), 1.30 (m, 1H), 0.88 (m, 3H), 0.81 (m, 2H). HRMS *m/z* calcd for $C_{28}H_{27}F_4N_6O_2$ $[M+H]^+$: 555.2126, Found 555.6 237.

12f (41 mg, 79 %); ^{1}H NMR (400 MHz, CD_3OD) δ 8.43 (d, *J* = 8.5 Hz, 1H), 8.42 – 8.34 (m, 1H), 8.18 (d, *J* = 8.5 Hz, 1H), 7.95 (d, *J* = 7.7 Hz, 1H), 7.69 (dd, *J* = 13.5, 6.1 Hz, 2H), 7.61 (dd, *J* = 12.6, 5.5 Hz, 2H), 7.32 (s, 1H), 7.05 (d, *J* = 8.9 Hz, 1H), 6.77 (s, 1H), 4.18 – 4.06 (br, 1H), 3.97 (m, 1H), 3.89 (s, 3H), 3.13 (m, 1H), 2.86 (m, 2H), 1.91 (m, 1H), 1.48 (m, 4H), 1.22 – 1.03 (m, 1H), 0.93 – 0.48 (m, 4H), 0.15 (m, 1H). HRMS *m/z* ca lcd for $C_{30}H_{30}N_7O_2$ $[M+H]^+$: 520.2455, Found 520.3653.

12g (37 mg, 84 %): ^{1}H NMR (400 MHz, $CDCl_3$) δ8.22 (s,1H), 7.63 (d,*J* = 32.3 Hz, 1H), 7.33 (s, 1H), 7.06 (d, *J* = 24.5 Hz, 2H), 6.95 (d, *J* = 7.2 Hz, 1H), 6.82 (d, *J* = 8.0 Hz, 1H), 6.26 (d, *J* = 32.3 Hz, 1H), 6.01 (s, 2H), 4.15 – 4.04 (s, 1H), 3.87 (s, 3H), 3.82 – 3.71 (m, 1H), 3.56 – 3.26 (m, 2H), 1.89 – 1.71 (m, 2H), 1.67 – 1.54 (m, 3H), 1.03 – 0.96 (m, 3H), 0.88 – 0.80 (m, 3H). HRMS *m/z* calcd for $C_{28}H_{29}N_6O_4$ $[M+H]^+$: 513.2245, Found 513.6853.

25a (31 mg, 52 %)1H NMR (400 MHz, CDCl3) d 8.20 (1H, s), 8.19 (1H, s),7.80–7.85 (3H, m), 7.78 (1H, d, J = 8.8 Hz), 7.51–7.54 (3H, m), 7.30 (1H, s), 7.02 (1H, dd, J = 8.8 Hz, J = 2.4 Hz), 6.20–6.38 (1H, m), 5.47 (1H, br, s), 3.87 (3H, s), 3.69–3.89 (2H, m), 3.40–3.62 (2H, m), 3.12–3.31 (1H, m), 1.54–1.78 (5H, m), 0.93–0.97 (2H, m), 0.72–0.75 (2H, m); HRMS (ESI) calcd for $C_{31}H_{31}N_6O_2$ [M+H]+: 519.2503, found 519.2940.

25b (76 mg, 60 %); ^{1}H NMR (400 MHz, $CDCl_3$)δ8.19(s,1H),7.67(d,*J* = 8.8 Hz, 1H), 7.24 (s, 1H), 7.07 (s, 1 H), 6.98 (dd, *J* = 8.4, 2.0 Hz, 1H), 6.93 (dd, *J* = 8.8, 2.2 Hz, 1H), 6.82 (d, *J* = 8.4 Hz, 1H), 6.24 (s, 1H), 4.24 (d, *J* = 5.0 Hz, 2H), 4.21 (d, *J* = 5.0 Hz, 2H), 4.15 (s, 1H), 3.87 (m, 1H), 3.44 (m, 1H), 3.35 – 3.09 (m, 1H), 1.73 (m, 2H), 1.67 – 1.48 (m, 3H), 1.04 – 0.98 (m, 1H), 0.92 (m, 1H), 0.86 – 0.78 (m, 2H), 0.73 (m, 1H), 0.34 (m, 1H). ; HRMS *m/z* calcd for $C_{29}H_{31}N_6O_4$ $[M+H]^+$: 527.2401, Found 527.6124.

25c (20 mg, 61 %); ^{1}H NMR (400 MHz, CD_3OD) δ 8.41 – 8.22 (m, 1H), 7.84 (d, *J* = 2.1 Hz, 2H), 7.69 – 7.60 (m, 1H), 7.59 – 7.52 (m, 1H), 7.42 (d, *J* = 8.3 Hz, 1H), 7.29 (m, 1H), 7.11 – 6.98 (m, 1H), 6.90 (s, 1H), 6.58 (s, 1H), 4.25 (m, 1H), 4.02 (s, 1H), 3.84 (s, 3H), 3.29 – 3.08 (m, 1H), 2.95 (m, 1H), 1.98 – 1.84 (m, 1H), 1.77 (s, 1H), 1.69 – 1.38 (m, 3H), 0.99 – 0.57 (m, 5H), 0.49 – 0.09 (m, 1H). ; HRMS *m/z* calcd for $C_{29}H_{29}N_6O_3$ $[M+H]^+$: 509.2296, Found 509.4000.

25d (16 mg, 39 %); ^{1}H NMR (400 MHz, CDCl3) δ 8.29 (s, 1H), 7.86 – 7.70 (m, 2H), 7.45 (d, J = 8.0 Hz, 1H), 7.35 – 7.28 (m, 1H), 7.20 (s, 1H), 7.01 (d, J = 8.8 Hz, 1H), 6.41 (s, 1H), 4.10 (s, 1H), 3.85 (s, 3H), 3.58 (s, 1H), 3.24 (m, 1H), 1.76 (m, 3H), 1.05 (m, 5H), 0.92 (m, 5H). HRMS *m/z* calcd for $C_{27}H_{27}Cl_2N_6O_2$ $[M+H]^+$: 537.1567, Found 537.2227.

25e (49 mg, 81 %); ^{1}H NMR (400 MHz, $CDCl_3$)δ8.23(s,1H),7.81(d,*J* = 32.4 Hz, 1H), 7.58 (m, 2H), 7.16 – 7.02 (m, 2H), 6.88 (dd, *J* = 8.8, 2.3 Hz, 1H), 6.29 (d, *J* = 32.4 Hz, 1H), 3.96 (s, 1H), 3.72 (s, 3H), 3.61 – 3.35 (m, 2H), 1.72 – 1.52 (m, 3H), 1.50 – 1.33 (m, 2H), 1.07 – 0.99 (m, 1H), 0.92 – 0.79 (m, 3H), 0.77 – 0.59 (m, 3H). HRMS *m/z* calcd for $C_{28}H_{27}F_4N_6O_2$ $[M+H]^+$: 555.2126, Found 555.6237.

25f (27 mg, 97 %); ^{1}H NMR (400 MHz, CD_3OD) δ 8.43 (d, *J* = 8.5 Hz, 1H), 8.42 – 8.34 (m, 1H), 8.18 (d, *J* = 8.5 Hz, 1H), 7.95 (d, *J* = 7.7 Hz, 1H), 7.69 (dd, *J* = 13.5, 6.1 Hz, 2H), 7.61 (dd, *J* = 12.6, 5.5 Hz, 2H), 7.32 (s, 1H), 7.05 (d, *J* = 8.9 Hz, 1H), 6.77 (s, 1H), 4.18 – 4.06 (br, 1H), 3.97 (m, 1H), 3.89 (s, 3H), 3.13 (m, 1H), 2.86 (m, 2H), 1.91 (m, 1H), 1.48 (m, 4H), 1.22 – 1.03 (m, 1H), 0.93 – 0.48 (m, 4H), 0.15 (m, 1H). HRMS *m/z* ca lcd for $C_{30}H_{30}N_7O_2$ $[M+H]^+$: 520.2455, Found 520.0187.

25g (60 mg, 66 %); ^{1}H NMR (400 MHz, $CDCl_3$)δ8.24(s,1H),7.67(d,*J* = 8.6 Hz, 1H), 7.24 (s, 1H), 7.02 (m, 2H), 6.94 (dd, *J* = 8.6, 2.0 Hz, 1H), 6.78 (d, *J* = 8.2 Hz, 1H), 6.35 – 6.14 (m, 1H), 5.98 (s, 2H), 4.16 (s, 1H),

3.81 (s, 3H), 2.01 (m, 1H), 1.74 (s, 2H), 1.66 (m, 2H), 1.29 – 1.18 (m, 1H), 1.13 (m, 2H), 1.00 (m, 3H), 0.85 (m, 2H), 0.74 (m, 1H). HRMS *m/z* calcd for $C_{28}H_{29}N_6O_4$ [M+H]$^+$: 513.2245, Found 513.2441.

2.2.7 General syntheses of (S)-cyclopropyl(3-((4-(5-hydroxy-2-(naphthalen-2-yl)-1H-benzo[d]imidazol-1-yl)pyrimidin-2-yl)amino)piperidin-1-yl)methanone (13a-13g, 26a-26g)

2.2.7.1. (S)-cyclopropyl(3-((4-(5-hydroxy-2-(naphthalen-2-yl)-1H-benzo[d]imidazol-1-yl)pyrimidin-2-yl) amino piperidin-1-yl)methanone (13a)

Compound **12a** (66 mg, 0.127 mmol) was dissolved in methylene chloride (1.3 ml), BBr_3 (60μl) was added at -78 °C, and the reaction was stirred for 1 h and then at room temperature for 2 h. After confirming the completion of the reaction, MeOH was added to quench the reaction, the organic solvent was removed under reduced pressure, and the residue was extracted with methylene chloride and washed with a saturated $NaHCO_3$ aqueous solution. The extracted organic layer was dried with anhydrous magnesium sulfate and filtered, the filtrate was concentrated under reduced pressure, and the residue was purified by column chromatography (silica gel, methylene chloride: MeOH = 20: 1), to give the target compound **13a** (39 mg, 61%) was obtained; ^{1}H NMR (400 MHz, DMSO-d_6) δ 9.36 (s, 1H), 8.42 – 8.18 (m, 2H), 7.96 – 7.94 (m, 3H), 7.61 – 7.54 (m, 5H), 7.11 (d, *J* = 2.4 Hz, 1H), 6.84 (d, *J* = 7.6 Hz, 1H), 6.67 – 6.25 (m, 1H), 4.78 (s, 1H), 4.14 – 3.84 (m, 2H), 3.17 – 2.85 (m, 2H), 1.97 – 1.91 (m, 2H), 1.75 (s, 1H), 1.45 – 1.14 (m, 4H), 0.85 – 0.69(m, 2H).; HRMS *m/z* calcd for $C_{15}H_{12}Cl_2N_4OS$ 367.2480, Found 368.2729 (M+H$^+$). HRMS (ESI) calcd for $C_{30}H_{29}N_6O_2$ [M+H]+: 505.2347, found 505.2722.

(S)-Cyclopropyl(3-((4-(2-(2,3-dihydrobenzo[b][1,4]dioxin-6-yl)-5-hydroxy-1H-benzo[d] imidazol-1-yl)pyrimidin-2-yl)amino)piperidin-1-yl)methanone ***(13b,*** 52 %);^{1}H NMR (400 MHz, CD_3OD) δ 8.33 (s, 1H), 7.63 – 7.41 (m, 1H), 7.09 (s, 1H), 7.03 (s, 1H), 6.97 (d, *J* = 8.4 Hz, 1H), 6.92 – 6.82 (m, 2H), 6.57 (s, 1H), 4.26 (d, *J* = 6.3 Hz, 4H), 4.07 (s, 1H), 3.49 (s, 1H), 2.97 (m, 1H), 2.02 (m, 2H), 1.79 (m, 2H), 1.61 (m, 4H), 0.95 – 0.79 (m, 3H), 0.69 (m, 2H). HRMS (ESI) calcd for $C_{28}H_{29}N_6O_4$ [M+H]+: 513.2245, found 513.0551.

(S)-(3-((4-(2-(Benzofuran-5-yl)-5-hydroxy-1H-benzo[d]imidazol-1-yl)pyrimidin-2-yl) amino)piperidin-1-yl)(cyclopropyl)methanone ***(13c,*** 57 %); ^{1}H NMR (400 MHz, DMSO) δ 9.33 (s, 1H), 8.33 (d, *J* = 47.4 Hz, 1H), 8.07 (s, 1H), 7.87 (d, *J* = 23.5 Hz, 1H), 7.65 (s, 2H), 7.45 (d, *J* = 18.7 Hz, 1H), 7.08 (d, *J* = 2.2 Hz, 1H), 7.02 (s, 1H), 6.82 (d, *J* = 7.4 Hz, 1H), 4.08 (s, 1H), 2.95 (s, 1H), 1.95 (m, 2H), 1.76 (m, 2H), 1.56 (m, 2H), 1.23 (m, 2H), 0.89 – 0.66 (m, 4H), 0.63 – 0.54 (m, 1H), 0.23 (m, 1H). ; ^{13}C NMR (101 MHz, DMSO) δ 176.04 (s), 174.74 (s), 171.96 (s), 165.03 (s), 154.13 (s), 152.23 (s), 147.17 (s), 146.98 (d, *J* = 4.0 Hz), 146.35 (s), 143.82 (s), 127.34 (d, *J* = 6.5 Hz), 125.56 (s), 122.32 (s), 113.03 (s), 107.09 (s), 104.00 (s), 100.58 (s), 91.72 (s), 61.51 (s), 53.26 (s), 50.02 (s), 46.33 (s), 29.73 (s), 10.40 (s), 6.90 (d, *J* = 6.8 Hz).; m.p. 125~127 °C; HRMS (ESI) calcd for $C_{28}H_{27}N_6O_3$ [M+H]+: 495.2139, found 495.6932.

(S)-Cyclopropyl(3-((4-(2-(3,4-dichlorophenyl)-5-hydroxy-1H-benzo[d]imidazol-1-yl) pyrimidin-2-yl)amino)piperidin-1-yl)methanone ***(13d,*** 41%): ^{1}H NMR (400 MHz, MeOD) δ 8.43 (d, *J* = 18.2 Hz, 1H), 7.76 (s, 1H), 7.59 (m, 2H), 7.40 (d, *J* = 8.4 Hz, 1H), 7.14 (s, 1H), 6.92 (d, *J* = 8.7 Hz, 1H), 6.78 (s, 1H), 4.20 (s, 1H), 4.08 (m, 1H), 3.15 (m, 1H), 2.92 (s, 1H), 2.06 – 1.93 (m, 1H), 1.83 (m, 2H), 1.57 (m, 3H), 1.28 (m, 1H), 0.92 – 0.77 (m, 3H), 0.65 (m, 1H), 0.36 (m, 1H). HRMS (ESI) calcd for $C_{26}H_{25}Cl_2N_6O_2$ [M+H]+: 523.1411, found 523.3586.

(S)-Cyclopropyl(3-((4-(2-(4-fluoro-3-(trifluoromethyl)phenyl)-5-hydroxy-1H-benzo[d] imidazol-1-yl)pyrimidin-2-yl)amino)piperidin-1-yl)methanone ***(13e***, 50%); ^{1}H NMR (400 MHz, CD_3OD) δ 8.49 – 8.37 (m, 1H), 7.92 (s, 1H), 7.80 (s, 1H), 7.64 – 7.49 (m, 1H), 7.45 (d, *J* = 9.5 Hz, 1H), 7.14 (s, 1H), 6.92 (d, *J* = 8.8 Hz, 1H), 6.77 (s, 1H), 4.19 (s, 1H), 2.93 – 2.72 (m, 1H), 2.05 (m, 1H), 1.91 – 1.73 (m, 3H), 1.72 – 1.64 (m, 1H), 1.61 – 1.50 (m, 2H), 0.94 – 0.85 (m, 2H), 0.84 – 0.78 (m, 2H), 0.74 (m, 1H), 0.62 (m, 1H).; ^{13}C NMR (101 MHz, DMSO) δ 170.91 (s), 169.52 (s), 162.02 (s), 161.66 (s), 158.47 (s), 154.40 (s), 143.61 (s), 135.67 (s), 127.89 (t, J = 57.6 Hz), 123.61 (s), 120.90 (s), 117.58 (d, J = 6.4 Hz), 117.36 (s), 116.89 (s), 113.96 (s), 104.20 (s), 52.76 (s), 47.85 (s), 41.99 (s), 29.88 (s), 24.65 (s), 23.21 (s), 10.48 (s), 6.81 (s).; m.p. 113~115 °C; HRMS (ESI) calcd for $C_{27}H_{25}F_4N_6O_2$ [M+H]+: 541.1970, found 541.3818.

(S)-Cyclopropyl(3-((4-(5-hydroxy-2-(quinolin-2-yl)-1H-benzo[d]imidazol-1-yl)pyrimidin-2-yl)amino)piperidin-

1-yl)methanone (**13f,** 30 %); ^{1}H NMR (400 MHz, CD_3OD) δ 8.49 – 8.41 (m, 2H), 8.19 (d, *J* = 8.5 Hz, 1H), 7.95 (d, *J* = 7.5 Hz, 1H), 7.74 – 7.67 (m, 2H), 7.61 (m, 2H), 6.95 (dd, *J* = 8.5, 2.1 Hz, 2H), 6.76 (s, 1H), 4.02 (s, 1H), 3.18 (s, 1H), 2.06 – 1.93 (m, 1H), 1.60 (m, 3H), 1.39 (m, 4H), 1.29 (m, 4H), 0.97 – 0.78 (m, 5H), 0.60 (m, 1H). ; ^{13}C NMR (101 MHz, DMSO) δ 155.75 (s), 148.43 (d, *J* = 4.0 Hz), 146.17 (s), 136.83 (d, *J* = 7.0 Hz), 135.99 (s), 135.58 (s), 130.21 (d, *J* = 2.0 Hz), 128.53 (d, *J* = 2.9 Hz), 127.99 (s), 127.37 (s), 127.31 (s), 120.76 (d, *J* = 4.9 Hz), 113.63 (d, *J* = 7.8 Hz), 113.52 (d, *J* = 6.7 Hz), 49.28 (s), 30.97 (s), 22.08 (s), 13.98 (s), 10.51 (d, *J* = 2.8 Hz), 6.84 (d, *J* = 5.7 Hz).; m.p. 139~141 °C; HRMS (ESI) calcd for $C_{29}H_{28}N_7O_2$ $[M+H]^+$: 506.2299, found 506.4381.

(S)-(3-((4-(2-(Benzo[d][1,3]dioxol-5-yl)-5-hydroxy-1H-benzo[d]imidazol-1-yl)pyrimidin-2-yl)amino)piperidin-1-yl)(cyclopropyl)methanone (**13g,** 89%); ^{1}H NMR (400 MHz, MeOD) δ 8.41 – 8.25 (m, 1H), 7.55 (m, 1H), 7.08 (s, 1H), 6.94 (s, 1H), 6.90 – 6.77 (m, 3H), 6.49 (m, 1H), 4.25 (s, 1H), 4.02 (m, 1H), 3.59 – 3.36 (m, 1H), 3.08 (m, 2H), 2.06 – 1.86 (m, 2H), 1.77 (s, 1H), 1.61 (m, 3H), 1.29 (m, 1H), 0.90 – 0.57 (m, 4H), 0.28 (m, 1H). HRMS (ESI) calcd for $C_{27}H_{27}N_6O_4$ $[M+H]^+$: 506.2299, found 506.4381.

(S)-Cyclopropyl(3-((4-(6-hydroxy-2-(naphthalen-2-yl)-1H-benzo[d]imidazol-1-yl)pyrimidin-2-yl)amino) piperidin-1-yl)methanone (**26a,** 93%); ^{1}H NMR (400 MHz, DMSO-d_6) δ 9.52 (s,1H), 8.3 (m, 2H), 7.92–7.95 (m, H), 7.55–7.61 (m, 5H), 6.84 (dd, *J* = 8.8, 2.0 Hz, 1H), 6.34 – 6.67 (m, 1H), 3.87 – 4.39 (m, 3H), 2.80 – 3.05 (m, 1H), 1.98 (m, 1H), 1.34 – 1.51 (m, 4H), 0.70 – 0.85 (m, 4H). ; ^{13}C NMR (100 MHz, DMSO-d_6) δ 162.2, 160.4, 157.3, 155.0, 136.1, 132.9, 132.5, 128.4 127.8, 127.6, 127.2, 126.8, 125.7, 120.1, 113.2, 113.0, 105.4, 97.5, 49.1, 48.0, 45.1, 29.6, 22.9, 10.5, 6.9 ppm; HRMS (ESI) calcd for $C_{30}H_{29}N_6O_2$ $[M+H]^+$: 505.2347, found 505.0201.

(S)-Cyclopropyl(3-((4-(2-(2,3-dihydrobenzo[b][1,4]dioxin-6-yl)-6-hydroxy-1H-benzo[d] imidazol-1-yl)pyrimidin-2-yl)amino)piperidin-1-yl)methanone (**26b,** 64 %); ^{1}H NMR (400 MHz, DMSO) δ 9.47 (s, 1H), 8.51 – 8.28 (m, 1H), 7.80 – 7.62 (m, 1H), 7.53 (d, *J* = 8.6 Hz, 1H), 7.01 (m, 1H), 6.90 (m, 2H), 6.79 (dd, *J* = 8.6, 2.1 Hz, 1H), 4.26 (m, 4H), 4.19 – 4.07 (m, 1H), 3.86 (s, br, 1H), 3.52 (m, 1H), 3.05 (m, 1H), 2.63 (m, 1H), 1.96 (m, 1H), 1.81 (m, 2H), 1.64 – 1.35 (m, 2H), 1.21 (m, 1H), 0.83 (m, 3H), 0.58 – 0.01 (m, 2H). HRMS (ESI) calcd for $C_{28}H_{29}N_6O_4$ $[M+H]^+$: 513.2245, found 513.3702.

(S)-(3-((4-(2-(Benzofuran-5-yl)-6-hydroxy-1H-benzo[d]imidazol-1-yl)pyrimidin-2-yl) amino)piperidin-1-yl)(cyclopropyl)methanone (**26c,** 18 %); ^{1}H NMR (400 MHz, CD_3OD) δ 8.35 (s, 1H), 7.84 (m, 2H), 7.56 (dd, *J* = 8.4, 3.8 Hz, 2H), 7.42 (d, *J* = 8.1 Hz, 1H), 7.27 – 7.14 (m, 1H), 7.04 (dd, *J* = 14.9, 8.4 Hz, 1H), 6.89 (d, *J* = 8.2 Hz, 2H), 4.22 (brs, 1H), 4.02 (s, 1H), 3.17 (m, 1H), 2.84 (m, 2H), 2.10 – 1.96 (m, 2H), 1.81 (m, 2H), 1.56 (m, 2H), 0.88 (m, 5H); HRMS (ESI) calcd for $C_{28}H_{27}N_6O_3$ $[M+H]^+$: 495.2139, found 495.6932.

(S)-Cyclopropyl(3-((4-(2-(3,4-dichlorophenyl)-6-hydroxy-1H-benzo[d]imidazol-1-yl)pyrimidin-2-yl)amino) piperidin-1-yl)methanone (**26d,** 57 %); ^{1}H NMR (400 MHz, CD_3OD) δ 8.39 (d, J = 57.2 Hz, 1H), 7.75 (s, 1H), 7.58 (t, J = 8.2 Hz, 2H), 7.37 (d, J = 6.7 Hz, 1H), 7.05 (s, 1H), 6.91 (d, J = 8.8 Hz, 1H), 6.42 (d, J = 5.3 Hz, 1H), 4.24 (d, J = 12.4 Hz, 1H), 3.60 (s, 1H), 3.27 – 3.13 (m, 1H), 3.09 – 2.61 (m, 2H), 2.01 (s, 1H), 1.92 – 1.50 (m, 5H), 1.02 – 0.81 (m, 3H), 0.66 – 0.22 (m, 2H). HRMS (ESI) calcd for $C_{26}H_{25}Cl_2N_6O_2$ $[M+H]^+$: 523.1411, found 523.1561.

(S)-Cyclopropyl(3-((4-(2-(4-fluoro-3-(trifluoromethyl)phenyl)-6-hydroxy-1H-benzo[d] imidazol-1-yl)pyrimidin-2-yl)amino)piperidin-1-yl)methanone (**26e,** 65 %); ^{1}H NMR (400 MHz, CD_3OD) δ 8.37 (s, 1H), 7.91 (d, J = 4.7 Hz, 1H), 7.77 (s, 1H), 7.67~7.54 (m, 1H), 7.42 (m, 1H), 7.02 (s, 1H), 6.90 (d, J = 8.7 Hz, 1H), 6.37 (s, 1H), 4.14 (s, 1H), 3.59 (s, 1H), 2.15 – 1.95 (m, 2H), 1.59 (m, 3H), 1.28 (m, 4H), 0.93 – 0.57 (m, 4H), 0.29 (m, 1H). ; HRMS(ESI) calcd for $C_{27}H_{25}F_4N_6O_2$ [M+H]+: 541.1970, found 541.3818.

(S)-Cyclopropyl(3-((4-(6-hydroxy-2-(quinolin-2-yl)-1H-benzo[d]imidazol-1-yl)pyrimidin-2-yl)amino)piperidin-1-yl)methanone (**26f,** 83 %); ^{1}H NMR (400 MHz, MeOD) δ 8.45 (m, 2H), 8.35 – 8.15 (m, 1H), 7.97 (s, 1H), 7.79 – 7.57 (m, 3H), 7.22 (s, 1H), 6.97 (m, 1H), 6.77 (s, 1H), 4.07 (m, 2H), 3.25 – 3.08 (s, 1H), 2.69 (m, 1H), 2.08 – 1.96 (m, 1H), 1.87 – 1.49 (m, 4H), 1.29 (m, 3H), 0.89 (m, 3H), 0.65 (m, 1H). ; ^{13}C NMR (101 MHz, DMSO) δ 154.83 (s), 144.03 (d, *J* = 5.1 Hz), 143.02 (s), 138.70 (d, *J* = 4.8 Hz), 137.54 (d, *J* = 18.9 Hz), 130.75 (d, *J* = 2.0 Hz), 129.18 (d, *J* = 11.7 Hz), 128.49 (s), 127.95 (d, *J* = 9.7 Hz), 115.20 (d, *J* = 4.2 Hz), 104.70 (d, *J* = 6.0 Hz), 104.63 (d, *J* = 3.9 Hz), 49.69 (s), 48.73 (s), 42.45 (s), 30.08 (s), 24.98 (s), 15.38 (s), 7.13 (d, *J* = 18.7

Hz). m.p. 147~149 °C; HRMS (ESI) calcd for $C_{29}H_{28}N_7O_2$ [M+H]+: 506.2299, found 506.4696.

(S)-(3-((4-(2-(Benzo[d][1,3]dioxol-5-yl)-6-hydroxy-1H-benzo[d]imidazol-1-yl)pyrimidin-2-yl)amino)piperidin-1-yl)(cyclopropyl)methanone (**26g**, 72%); ^{1}H NMR (400 MHz, DMSO) δ 9.34 (d, *J* = 46.0 Hz, 2H), 8.48 – 8.23 (m, 1H), 7.70 (d, *J* = 32.8 Hz, 1H), 7.48 (d, *J* = 8.6 Hz, 1H), 6.96 (s, 1H), 6.76 (d, *J* = 6.1 Hz, 2H), 4.19 (d, *J* = 11.6 Hz, 1H), 3.88 (d, *J* = 68.9 Hz, 1H), 2.87 (d, *J* = 96.9 Hz, 1H), 2.11 – 1.75 (m, 3H), 1.74 – 1.37 (m, 3H), 1.20 (d, *J* = 23.5 Hz, 2H), 0.88 – 0.63 (m, 3H), 0.52 (d, *J* = 37.8 Hz, 1H), 0.14 (d, *J* = 90.2 Hz, 1H). ; ^{13}C NMR (101 MHz, DMSO) δ 170.93 (s), 162.36 (s), 155.79 (d, J = 4.7 Hz), 154.36 (s), 146.34 (s), 143.57 (d, J = 5.6 Hz), 136.88 (s), 130.22 (s), 128.34 (d, J = 68.6 Hz), 127.46 (d, J = 16.7 Hz), 120.80 (s), 114.71 (s), 113.64 (s), 104.19 (s), 49.25 (s), 41.96 (d, J = 3.4 Hz), 29.00 (s), 22.09 (s), 13.96 (s), 6.69 (d, J = 22.8 Hz).; m.p.118~119 °C; HRMS (ESI) calcd for $C_{27}H_{27}N_6O_4$ [M+H]+: 506.2299, found 506.4381.

2.3.4. Syntheses of 2-Aryl-5, 6-dihydroxy-1-pyrimidin-4-yl-11H-imidazol derivatives (Scheme 2)

2.3.4.1. 4,5-dimethoxybenzene-1,2-diamine (28)

Compound **27** (5 g, 21.9 mmol) was dissolved in MeOH (147 ml), and then 10% Pd/C (639 mg) was added, followed by stirring at room temperature for 6 h under hydrogen gas. After the reaction was terminated, it was filtered with celite and the filtrate was distilled under reduced pressure. The target compound **28** was obtained without further purification of the residue (dark brown solid, 99%).;^{1}H NMR (400 MHz, DMSO) δ 6.26 (s, 2H), 4.11 (s, 4H), 3.58 (s, 6H).; HRMS (ES$^+$) calcd for $C_8H_{12}N_2O_2$ [M+H]$^+$: 170.1044, Found 170.2144.

2.3.4.2. Syntheses of 5,6-dimethoxy-2-aryl-1H-benzo[d]imidazole derivartives (29)

Compound **28** (2.97 mmol) and $Na_2S_2O_5$ (148.5 mmol) were dissolved in DMF (29.7 ml), followed by stirring in a microwave at 120 °C, 150 W, 1 h 30 min. After the completion of the reaction, the solvent was poured into ice water to precipitate. After filtering the precipitated reaction product, the filtrate was distilled under reduced pressure, and the crude product was purified using flash column chromatography on silica gel using a mobile phase of EA: HEX (3:1) to obtain compound **29a (**as a yellow solid, 50%) ; ^{1}H NMR (400 MHz, DMSO) δ 12.79 (s, 1H), 8.61 (s, 1H), 8.25 (dd, *J* = 8.6, 1.7 Hz, 1H), 8.04 (d, *J* = 8.7 Hz, 1H), 8.02 – 7.94 (m, 2H), 7.61 – 7.53 (m, 2H), 7.14 (d, *J* = 76.5 Hz, 2H), 3.83 (s, 6H). ; HRMS (ESI) calcd for $C_{19}H_{17}N_2O_2$ [M+H]$^+$: 305.1285, Found 305.9705.

29b (as a white solid, 45%); ^{1}H NMR (400 MHz, CDCl$_3$) 7.76 (d, *J* = 8.9 Hz, 1H), 7.30 (d, *J* = 2.3 Hz, 1H), 7.13 (d, *J* = 2.1 Hz, 1H), 7.00 – 6.93 (m, 2H), 4.28 (m, 2H), 4.27 – 4.23 (m, 2H), 3.87 (s, 6H). ; HRMS (ESI) calcd for $C_{17}H_{17}N_2O_4$ [M+H]$^+$: 313.1183, Found 313.1690.

29c (as a yellow solid, 67%) ; ^{1}H NMR (400 MHz, CDCl$_3$) δ 8.34 (s, 1H), 8.06 (d, *J* = 8.3 Hz, 1H), 7.58 (s, 1H), 7.45 (d, *J* = 8.5 Hz, 1H), 6.91 (s, 2H), 6.59 (s, 1H), 3.66 (s, 6H). ; HRMS (ESI) calcd for $C_{17}H_{15}N_2O_3$ [M+H]$^+$: 295.1077, Found 295.2672.

29d (as a yellow solid, 56%); ^{1}H NMR (400 MHz, CDCl$_3$) δ 9.15 (s, 1H), 8.05 (d, *J* = 1.5 Hz, 1H), 7.82 (dd, *J* = 8.4, 1.6 Hz, 1H), 7.35 (d, *J* = 8.4 Hz, 1H), 7.02 (s, 2H), 3.80 (s, 6H). ; HRMS (ESI) calcd for $C_{15}H_{13}Cl_2N_2O_2$ [M+H]$^+$: 323.0349, Found 323.3259.

29e (as a yellow solid, 65%); ^{1}H NMR (400 MHz, CDCl$_3$) δ 8.23 – 8.16 (m, 2H), 7.15 (m, 1H), 7.09 (s, 2H), 3.84 (s, 6H); HRMS (ESI) calcd for $C_{16}H_{13}F_4N_2O_2$ [M+H]$^+$: 341.0908, Found 341.5966.

2.3.4.3. Syntheses of 5,6-dimethoxy-1-(2-(methylthio)pyrimidin-4-yl)-2-aryl-1H-benzo[d]imidazole (30)

Compounds **29** (1.3 mmol), 4-chloro-2-(methylthio)pyrimidine (4-chloro-2-(methylthio)pyrimidine (320 mg, 1.3 mmol), palladium (II) acetate (88 mg, 0.13 mmol), X-Phos (62 mg, 0.13 mmol), and Cs_2CO_3 were purged with nitrogen, and then toluene (13 mL) was added and mixed, sonication under nitrogen

for 5 minutes. After sonication, the mixture was heated under nitrogen to 130° C, and stirred without nitrogen for 3 hours at 130° C. After cooling to ambient temperature, the reaction mixture was filtered through a celite pad. Then, the solvent was removed *in vacuo*, and purified by flash column chromatography on silica gel using a mobile phase of MC:MeOH (40:1). Compounds **30a** (as pale yellow solids, 27%) ; ^{1}H NMR (400 MHz, $CDCl_3$) δ 8.37 (d, *J* = 5.4 Hz, 1H), 8.22 (s, 1H), 7.88 – 7.83 (m, 3H), 7.59 – 7.49 (m, 4H), 7.41 (s, 1H), 6.54 (d, *J* = 5.4 Hz, 1H), 3.99 (s, 3H), 3.96 (s, 3H), 2.56 (s, 3H). ; HRMS (ESI) calcd for $C_{24}H_{21}N_4O_2S$ [M+H]+ : 429.1380, Found 429.3940.

30b (as a yellow solid, 42%) ; ^{1}H NMR (400 MHz, $CDCl_3$) δ 8.41 (d, *J* = 5.4 Hz, 1H), 7.44 (s, 1H), 7.30 (s, 1H), 7.08 (d, *J* = 2.0 Hz, 1H), 6.96 (dd, *J* = 8.4, 2.1 Hz, 1H), 6.84 (d, *J* = 8.4 Hz, 1H), 6.54 (d, *J* = 5.4 Hz, 1H), 4.29 – 4.25 (m, 2H), 4.23 (dd, *J* = 3.7, 1.5 Hz, 2H), 3.93 (s, 3H), 3.90 (s, 3H), 2.57 (s, 3H). ; LRMS (ESI) calcd for $C_{22}H_{20}N_4O_4S$ [M+H]+ : 437.1278, Found 437.4296.

30c (as a yellow solid,58%) ; ^{1}H NMR (400 MHz, DMSO) δ 9.07 (d, *J* = 5.5 Hz, 1H), 8.15 (d, *J* = 2.4 Hz, 1H), 8.03 (d, *J* = 1.7 Hz, 1H), 8.01 (d, *J* = 9.1 Hz, 1H), 7.76 (d, *J* = 8.6 Hz, 1H), 7.56 (dd, *J* = 8.6, 1.7 Hz, 1H), 7.43 (m, 2H), 7.16 (dd, *J* = 9.1, 2.4 Hz, 1H), 7.09 (d, *J* = 1.5 Hz, 1H), 3.88 (s, 6H), 3.36 (s, 3H). ; HRMS (ESI) calcd for $C_{22}H_{19}N_4O_5S$ [M+H]+ : 419.1172, Found 419.2470.

30d (as a yellow solid, 35%); ^{1}H NMR (400 MHz, $CDCl_3$) δ 8.47 (d, *J* = 5.4 Hz, 1H), 7.73 (d, *J* = 2.0 Hz, 1H), 7.41 (d, *J* = 8.3 Hz, 1H), 7.35 (s, 1H), 7.24 (dd, *J* = 8.5, 2.2 Hz, 2H), 6.57 (d, *J* = 5.4 Hz, 1H), 3.92 (s, 3H), 3.89 (s, 3H), 2.50 (s, 3H). ; LRMS (ESI) calcd for $C_{20}H_{17}Cl_2N_4O_2S$ [M+H]+ : 447.0444, Found 447.2927.

30e (as a yellow solid, 37%); ^{1}H NMR (400 MHz, $CDCl_3$) δ 8.52 (d, *J* = 5.3 Hz, 1H), 7.92 (d, *J* = 5.0 Hz, 1H), 7.67 – 7.60 (m, 1H), 7.30 (d, *J* = 6.3 Hz, 2H), 7.21 (t, *J* = 9.2 Hz, 1H), 6.64 (d, *J* = 5.3 Hz, 1H), 3.92 (d, *J* = 9.7 Hz, 6H), 2.46 (s, 3H). ; HRMS (ESI) calcd for $C_{21}H_{17}F_4N_4O_2S$ [M+H]+ : 465.1003, Found 465.4117.

2.3.4.4. Syntheses of 5,6-dimethoxy-1-(2-(methylsulfonyl)pyrimidin-4-yl)-2-aryl-1H-benzo[d]imidazole (31)

Compound **30** (1 mmol) and potassium peroxomonosulfate (5 mmol) were stirred in methanol: water = 1: 1 (5 ml) at ambient temperature for 1 hour. When compounds 30a to 30e disappeared from TLC, methanol was concentrated in vacuo. After adding water to the mixture, it was diluted and stirred until the product was separated into a solid. The solid product was filtered and washed with water. The crude product was then crystallized to give compounds **31a** (as a brown solid, 68%) ; ^{1}H NMR (400 MHz, DMSO) δ 9.03 (d, *J* = 5.6 Hz, 1H), 8.29 (s, 1H), 8.04 – 7.99 (m, 3H), 7.80 (s, 1H), 7.64 (m, 3H), 7.47 (d, *J* = 5.6 Hz, 1H), 7.42 (s, 1H), 3.89 (s, 3H), 3.86 (s, 3H), 3.35 (s, 3H). ; HRMS (ESI) calcd for $C_{24}H_{21}N_4O_4S$ [M+H]+ : 461.1278, Found 461.4098.

31b (as a white solid, 65%) ; ^{1}H NMR (400 MHz, DMSO) δ 9.12 (d, *J* = 5.5 Hz, 1H), 7.93 (d, *J* = 9.0 Hz, 1H), 7.51 (d, *J* = 5.5 Hz, 1H), 7.37 (d, *J* = 2.4 Hz, 1H), 7.19 (d, *J* = 2.0 Hz, 1H), 7.10 (dd, *J* = 9.0, 2.4 Hz, 1H), 7.02 (dd, *J* = 8.4, 2.0 Hz, 1H), 6.97 (d, *J* = 8.4 Hz, 1H), 4.33 (d, *J* = 4.7 Hz, 2H), 4.30 (d, *J* = 4.7 Hz, 2H), 3.86 (s, 6H), 3.41 (s, 3H). ; HRMS (ESI) calcd for $C_{22}H_{21}N_4O_6S$ [M+H]+ : 469.1176, Found 469.2560.

31c (as a white solid, 70%) ; ^{1}H NMR (400 MHz, $CDCl_3$) δ 8.33 (d, *J* = 5.4 Hz, 1H), 7.83 (d, *J* = 1.4 Hz, 1H), 7.66 (d, *J* = 2.2 Hz, 1H), 7.50 (m, 2H), 7.41 (dd, *J* = 8.6, 1.8 Hz, 1H), 7.32 (s, 1H), 6.77 (dd, *J* = 2.2, 0.9 Hz, 1H), 6.43 (d, *J* = 5.4 Hz, 1H), 3.94 (d, *J* = 7.7 Hz, 6H), 2.55 (s, 3H). ; HRMS (ESI) calcd for $C_{22}H_{19}N_4O_5S$ [M+H]+ : 451.1071 Found 451.2001.

31d (as a white solid, 60%) ; ^{1}H NMR (400 MHz, DMSO) δ 9.12 (d, *J* = 5.5 Hz, 1H), 7.94 (d, *J* = 2.0 Hz, 1H), 7.73 – 7.69 (m, 2H), 7.61 (d, *J* = 5.5 Hz, 1H), 7.52 – 7.49 (m, 1H), 7.38 (s, 1H), 3.86 (s, 3H), 3.83 (s, 3H), 3.39 (s, 3H). ; HRMS (ESI) calcd for $C_{20}H_{17}Cl_2N_4O_4S$ [M+H]+ : 479.0342, Found 479.2764.

31e (as a white solid, 70%) ; 1H NMR (400 MHz, DMSO) δ 9.11 (d, J = 5.6 Hz, 1H), 8.07 (dd, J = 6.7, 1.9 Hz, 1H), 7.88 (m, 1H), 7.72 (d, J = 12.8 Hz, 1H), 7.63 (d, J = 5.6 Hz, 1H), 7.59 (d, J = 10.3 Hz, 1H), 7.41 (s, 1H), 3.85 (d, J = 8.6 Hz, 6H), 3.16 (s, 3H). ; HRMS (ESI) calcd for $C_{21}H_{17}F_4N_4O_4S$ [M+H]+ : 497.0901, Found 497.3002.

2.3.4.5. Syntheses of tert-butyl3-((4-(5,6-dimethoxy-2aryl-1H-benzo[d]imidazol-1-yl)pyrimidin-2-yl)amino)piperidine-1-carboxylate (32)

Compounds **31** (1 mmol) and (S)-tert-butyl 3-aminopiperidine-1-carboxylate (2 mmol) were stirred in THF (10 ml) at 60° C. for 12 hours. After compounds 31a-31e disappeared from TLC, the reaction mixture was cooled to ambient temperature and concentrated in vacuo. The crude product was purified by flash column chromatography on silica gel using a mobile phase of Hex:EA (1:1) to obtain compounds **32a** (as a yellow solid, 37%) ; ^{1}H NMR (400 MHz, MeOD) δ 8.28 (s, 1H), 8.13 (s, 1H), 7.93 – 7.88 (m, 3H), 7.57 (m, 3H), 7.47 (s, 1H), 7.32 (s, 1H), 6.52 (s, 1H), 3.94 (s, 3H), 3.92 (s, 3H), 3.69 – 3.58 (s, 1H), 3.20 (m, 1H), 2.79 (m, 2H), 1.45 (s, 9H), 1.12 (m, 4H), 0.91 (m, 2H). ; HRMS (ESI) calcd for $C_{33}H_{37}N_6O_4$ [M+H]+ : 581.2871, Found 581.4050

32b (as a yellow solid, 57%) ; ^{1}H NMR (400 MHz, $CDCl_3$) δ8.23(d,*J* = 5.0 Hz, 1H), 7.69 (d, *J* = 8.5 Hz, 1H), 7.31 (s, 1H), 7.14 (s, 1H), 7.04 (d, *J* = 7.9 Hz, 1H), 6.96 (d, *J* = 2.0 Hz, 1H), 6.85 (d, *J* = 8.4 Hz, 1H), 6.23 (s, 1H), 4.28 (d, *J* = 4.9 Hz, 2H), 4.24 (d, *J* = 4.9 Hz, 2H), 3.86 (s, 6H), 3.78 (s, 1H), 3.45 (m, 2H), 3.37 (m, 1H), 1.66 (m, 2H), 1.53 (m, 2H), 1.43 (s, 9H), 1.23 (m, 2H). ; HRMS (ESI) calcd for $C_{31}H_{37}N_6O_6$ [M+H]+ : 589.2769, Found 589.4319.

32c (as a yellow solid, 65%) ; ^{1}H NMR (400 MHz, $CDCl_3$) δ 8.33 (d, *J* = 5.4 Hz, 1H), 7.83 (d, *J* = 1.4 Hz, 1H), 7.66 (d, *J* = 2.2 Hz, 1H), 7.50 (m, 2H), 7.41 (dd, *J* = 8.6, 1.8 Hz, 1H), 7.32 (s, 1H), 6.77 (dd, *J* = 2.2, 0.9 Hz, 1H), 6.43 (d, *J* = 5.4 Hz, 1H), 3.94 (d, *J* = 7.7 Hz, 6H), 2.55 (s, 3H). ; HRMS (ESI) calcd for $C_{31}H_{35}N_6O_5S$ [M+H]+ : 571.2663, Found 571.6614.

32d (as a yellow solid, 51%) ; ^{1}H NMR (400 MHz, $CDCl_3$) δ 8.31 (s, 1H), 7.77 (s, 1H), 7.44 (d, *J* = 8.3 Hz, 1H), 7.32 (dd, *J* = 7.5, 2.9 Hz, 2H), 7.28 – 7.24 (m, 1H), 6.31 (s, 1H), 3.96 (s, 3H), 3.92 (s, 3H), 3.84 – 3.59 (m, 2H), 3.32 (s, 2H), 1.71 (m, 2H), 1.59 – 1.47 (m, 2H), 1.42 (m, 8H), 0.90 (m, 1H). ; HRMS (ESI) calcd for $C_{29}H_{33}Cl_2N_6O_4S$ [M+H]+ : 599.1935, Found 599.5143.

32e (as a yellow solid, 53%) ; ^{1}H NMR (400 MHz, $CDCl_3$) δ 8.33 (s, 1H), 7.93 (d, *J* = 4.9 Hz, 1H), 7.67 (s, 1H), 7.31 (s, 1H), 7.21 (m, 2H), 6.34 (d, J = 3.4 Hz, 1H), 3.93 (d, J = 15.5 Hz, 6H), 3.71 – 3.57 (s, 1H), 3.53 – 3.36 (m, 1H), 3.30 (m, 2H), 1.69 (m, 2H), 1.46 – 1.42 (m, 3H), 1.40 (s, 9H), 1.22 (m, 1H). ; HRMS (ESI) calcd for $C_{30}H_{33}F_4N_6O_4$ [M+H]+ : 617.2494, Found 617.4105.

2.3.4.6. Syntheses of 4-(5,6-dimethoxy-2-aryl-1H-benzo[d]imidazol-1-yl)-N-(piperidin-3-yl)pyrimidin-2-amine (33)

Compounds **32** (0.033 mmol) were dissolved in 0.33 ml of 1,4-dioxane and treated with 4 M-HCl (0.17 ml) in 1,4-dioxane at ambient temperature. The reaction mixture was stirred at ambient temperature for 20 minutes. The mixture was diluted with ether and stirred until the product separated into a solid. The solid product was filtered and washed with ether followed by hexane. The crude product was then crystallized to obtain compounds **33a (**as a white solid, 69%); ^{1}H NMR (400 MHz, MeOD) δ 8.38 (s, 1H), 8.10 (s, 1H), 7.95 – 7.86 (m, 3H), 7.61 – 7.52 (m, 3H), 7.32 (s, 2H), 6.74 (s, 1H), 3.94 (s, 3H), 3.92 (s, 3H), 3.73 – 3.46 (s, 1H), 3.19 – 2.92 (m, 2H), 2.68 (m, 2H), 1.52 (m, 2H), 1.29 (m, 2H), 0.98 – 0.80 (m, 2H). ; HRMS (ESI) calcd for $C_{28}H_{29}N_6O_2$ $[M+H]^+$: 481.2347, Found 481.0066.

33b (as a yellow solid, 86%) ; ^{1}H NMR (400 MHz, MeOD) δ 8.49 (s, 1H), 7.30 (d, *J* = 52.5 Hz, 2H), 7.10 (s, 1H), 7.03 (s, 1H), 6.94 (d, *J* = 6.7 Hz, 1H), 6.63 (d, *J* = 131.7 Hz, 1H), 4.30 (d, *J* = 8.5 Hz, 4H), 3.93 (d, *J* = 10.6 Hz, 6H), 3.67 (s, 1H), 3.43 – 3.26 (m, 4H), 3.03 (s, 2H), 2.19 – 1.96 (m, 2H), 1.90 – 1.71 (m, 2H), 1.28 (s, 1H). ; HRMS (ESI) calcd for $C_{26}H_{29}N_6O_4$ [M+H]+ : 489.2245, Found 490.3050.

33c (as a yellow solid, 89%) ; ^{1}H NMR (400 MHz, DMSO) δ 8.51 (s, 1H), 8.18 (s, 1H), 8.08 (d, *J* = 13.9 Hz, 1H), 7.82 (d, *J* = 8.6 Hz, 1H), 7.57 (d, *J* = 8.6 Hz, 1H), 7.41 (s, 1H), 7.38 (s, 1H), 7.15 (s, 1H), 6.62 (s, 1H), 3.97 (s, 1H), 3.90 (d, *J* = 7.4 Hz, 6H), 3.23 – 2.99 (m, 2H), 2.74 (s, 3H), 2.02 – 1.70 (m, 2H), 1.47 (d, *J* = 87.5, 3H). ; HRMS (ESI) calcd for $C_{26}H_{27}N_6O_3$ [M+H]+ : 471.2139, Found 471.3042.

33d (as a yellow solid, 78%) ; ^{1}H NMR (400 MHz, MeOD) δ 8.63 (s, 1H), 7.98 (s, 1H), 7.81 (d, *J* = 7.6 Hz, 1H), 7.62 (s, 1H), 7.41 (m, 2H), 6.90 (s, 1H), 4.32 (s, 1H), 4.00 (s, 3H), 3.96 (s, 3H), 3.68 – 3.50 (m, 1H), 3.37 (m, 1H), 3.00 (m, 2H), 2.03 (m, 2H), 1.79 (m, 2H), 1.64 (m, 1H). ; HRMS (ESI) calcd for $C_{24}H_{25}Cl_2N_6O_2$ [M+H]+ : 499.1411, Found 499.4429.

33e (as a white solid, 80%) ; ^{1}H NMR (400 MHz, $CDCl_3$) δ 8.43 (d, *J* = 21.5 Hz, 1H), 7.93 – 7.76 (m, 2H), 7.39 (s, 1H), 7.31 (m, 1H), 7.17 (s, 1H), 6.56 (s, 1H), 3.98 (s, 3H), 3.93 (s, 3H), 3.70 (s, 1H), 3.46 (m, 1H), 3.33 (s, 1H), 2.12 – 1.87 (m, 1H), 1.65 (m, 4H), 1.24 (m, 2H), 0.87 (m, 1H). ; HRMS (ESI) calcd for $C_{25}H_{25}F_4N_6O_2$ [M+H]+ : 517.1970, Found 517.4329.

2.3.4.7. Syntheses of cyclopropyl(3-((4-(5,6-dimethoxy-2-aryl-1H-benzo[d]imidazol-1-yl)pyrimidin-2-yl)amino)piperidin-1-yl)methanone (34)

Compounds **33a** (0.024 mmol) were cooled to 0°C in THF (0.24 ml) and treated with TEA (5 μL, 0.038 mmol). The mixture was added cyclopropanecarbonyl chloride (6.5 mg, 0.024 mmol) at 0 °C, and the mixture was raised to ambient temperature and stirred for 1 hour. The reaction mixture was concentrated in vacuo, diluted with methylene chloride, washed with water and a saturated aqueous sodium chloride solution. The organic phase was dried over sodium sulfate and concentrated in vacuo. The crude product was purified by flash column chromatography on silica gel using a mobile phase of DCM:MEOH (40:1) to obtain compounds **34a** (as a yellow solid,74%) ; ^{1}H NMR (400 MHz, MeOD) δ 8.33 (d, *J* = 38.9 Hz, 1H), 8.11 (d, *J* = 7.0 Hz, 1H), 7.95 – 7.84 (m, 3H), 7.59 – 7.51 (m, 2H), 7.48 (s, 1H), 7.33 (m, 2H), 6.66 (d, *J* = 42.4 Hz, 1H), 4.13 (s, 1H), 3.94 (s, 3H), 3.90 (s, 3H), 3.35 (s, 1H), 3.15 (m, 1H), 3.01 – 2.57 (m, 2H), 1.96 (m, 1H), 1.61 – 1.40 (m, 2H), 1.28 (m, 2H), 0.95 – 0.76 (m, 3H), 0.60 (m, 1H), 0.32 (m, 1H). ; HRMS (ESI) calcd for $C_{32}H_{33}N_6O_3$ [M+H]+ : 549.2609, Found 549.4281.

34b (as a yellow solid, 38%) ; 1H NMR (400 MHz, MeOD) δ 8.32 (s, 1H), 7.23 (s, 2H), 7.00 (s, 1H), 6.93 (d, J = 8.6 Hz, 1H), 6.86 (d, J = 8.6 Hz, 1H), 6.51 (s, 1H), 4.29 – 4.24 (m, 4H), 3.90 (s, 3H), 3.85 (s, 3H), 3.54 (s, 1H), 3.16 – 2.90 (m, 1H), 1.90 – 1.74 (m, 2H), 1.72 – 1.37 (m, 4H), 1.27 (m, 1H), 0.93 – 0.75 (m, 3H), 0.63 (s, 1H), 0.43 – 0.07 (m, 1H). ; HRMS (ESI) calcd for $C_{30}H_{33}N_6O_5$ [M+H]+ : 557.2507, Found 557.3567.

34c (as a yellow solid, 38%) ; ^{1}H NMR (400 MHz, MeOD) δ 8.30 (s, 1H), 7.94 – 7.77 (m, 2H), 7.57 (d, *J* = 8.6 Hz, 1H), 7.42 (m, 2H), 7.29 (s, 1H), 6.91 (s, 1H), 6.56 (s, 1H), 4.10 – 3.97 (s, 1H), 3.93 (s, 3H), 3.89 (s, 3H), 3.68 – 3.56 (m, 1H), 2.93 (m, 1H), 1.88 – 1.73 (m, 2H), 1.63 – 1.51 (m, 2H), 1.29 (m, 2H), 0.92 – 0.83 (m, 5H). ; HRMS (ESI) calcd for $C_{30}H_{31}N_6O_4$ [M+H]+ : 539.2401, Found 539.3022.

34d (as a yellow solid, 52%) ; ^{1}H NMR (400 MHz, MeOD) δ 8.51 – 8.39 (m, 1H), 7.78 (s, 1H), 7.61 (dd, *J* = 8.2, 5.4 Hz, 1H), 7.39 (dd, *J* = 14.6, 8.2 Hz, 1H), 7.32 (d, *J* = 8.7 Hz, 2H), 6.60 (m, 1H), 4.28 (d, *J* = 13.4 Hz, 1H), 4.19 – 4.04 (s, 1H), 3.93 (d, *J* = 14.9 Hz, 6H), 2.89 (s, 1H), 1.83 (m, 2H), 1.62 (m, 3H), 1.41 – 1.25 (m, 2H), 0.93 – 0.75 (m, 3H), 0.67 (m, 1H), 0.34 (m, 1H). ; HRMS (ESI) calcd for $C_{28}H_{29}Cl_2N_6O_3$ [M+H]+ : 567.1613, Found 567.4708.

34e (as a yellow solid, 38%) ; ^{1}H NMR (400 MHz, MeOD) δ 8.50 – 8.39 (m, 1H), 7.92 (s, 1H), 7.78 (s, 1H), 7.43 (m, 1H), 7.30 (s, 2H), 6.60 (s, 1H), 4.28 (m, 1H), 4.14 (s, 1H), 3.93 (s, 3H), 3.89 (s, 3H), 2.95 – 2.74 (m, 1H), 2.06 – 1.92 (m, 1H), 1.75 (m, 2H), 1.68 – 1.46 (m, 2H), 1.28 (m, 2H), 0.89 (m, 3H), 0.62 (m, 1H), 0.44 – 0.10 (m, 1H). ; HRMS (ESI) calcd for $C_{29}H_{29}F_4N_6O_3$ [M+H]+ : 585.2232, Found 585.3674.

2.3.4.8. Syntheses of cyclopropyl(3-((4-(5,6-dihydroxy-2-aryl-1H-benzo[d]imidazol-1-yl)pyrimidin-2-yl)amino)piperidin-1-yl)methanone (35)

Compound **34a** (0.053 mmol) was dissolved in methylene chloride (0.53 ml), 1 M boron tribromide (25μl) was added at -78 °C, and the reaction was stirred for 1 h and then at room temperature for 2 h. The mixture was quenched with methanol (0.2 ml) at 0 °C and stirred for an additional hour at room temperature. The mixture was diluted with methylene chloride (5 ml) and washed 3 times with saturated sodium bicarbonate solution (3 ml), 2 times with 5 ml of water, and 2 times with 5 ml of saturated sodium chloride solution. The organic phase was dried over sodium sulfate and concentrated in vacuo to obtain a white solid product. The crude product was purified by flash column chromatography on silica gel using a mobile phase of CH_2Cl_2: MeOH (40:1) to give product **35a** (as a yellow solid, 62%); ^{1}H NMR (400 MHz, DMSO) δ 8.24 (s, 1H), 8.12 (s, 1H), 7.93 (m, 3H), 7.61 – 7.52 (m, 3H), 7.49 (s, 1H), 7.09 (s, 1H), 6.26 (d, *J* = 4.7 Hz, 1H), 3.83 (s, 1H), 3.41 (m, 2H), 3.17 (m, 2H), 2.94 (m, 2H), 1.99 (s, 1H), 1.43 (m, 4H), 0.89 – 0.66 (m, 4H), 0.57 (m, 1H).; HRMS (ESI) calcd for $C_{30}H_{29}N_6O_3$ [M+H]+ : 521.2296, Found 521.0140.

(S)-Cyclopropyl(3-((4-(2-(2,3-dihydrobenzo[b][1,4]dioxin-6-yl)-5,6-dihydroxy-1H-benzo[d]imidazol-1-yl) pyrimidin-2-yl)amino)piperidin-1-yl)methanone (***35b,*** as a yellow solid, 42%) ; ^{1}H NMR (400 MHz, MeOD) δ 8.30 (s, 1H), 7.51 (s, 1H), 7.12 (d, *J* = 5.9 Hz, 1H), 7.02 (s, 1H), 6.95 (d, *J* = 2.9 Hz, 1H), 6.93 – 6.85 (m, 1H), 6.20 (d, *J* = 5.0 Hz, 1H), 4.69 (s, 1H), 4.29 (m, 4H), 3.93 (s, 1H), 3.57 (s, 1H), 3.22 (m, 1H), 2.87 (m, 1H), 1.96 (m, 3H), 1.61 (m, 3H), 1.31 (m, 1H), 1.06 – 0.62 (m, 4H), 0.34 (m, 1H). ; ^{13}C NMR (101 MHz, DMSO) δ 170.99 (s), 160.18 (s), 157.75 (s), 148.90 (s), 144.28 (s), 144.11 (s), 143.46 (d, J = 4.0 Hz), 143.10 (s), 135.67 (s), 128.78 (s), 123.92 (s), 122.09 (s), 117.05 (s), 104.84 (s), 104.13 (s), 97.64 (s), 96.37 (s), 64.14 (d, *J* = 20.5 Hz), 49.20 (s), 42.06 (s), 40.34 (s), 29.83 (s), 24.69 (s), 10.48 (s), 6.84 (d, *J* = 6.7 Hz).; m.p. 130~131 °C; HRMS (ESI) calcd for $C_{28}H_{28}N_6O_5$ [M+H]+ : 529.2194, Found 529.3455.
(S)-(3-((4-(2-(Benzofuran-5-yl)-5,6-dihydroxy-1H-benzo[d]imidazol-1-yl)pyrimidin-2-yl)amino)piperidin-1-yl)(cyclopropyl)methanone (***35c,*** as a white solid, 42%) ; ^{1}H NMR (400 MHz, MeOD) δ 8.36 – 8.07 (m, 1H), 7.84 (d, *J* = 1.9 Hz, 1H), 7.80 (s, 1H), 7.56 (m, 2H), 7.41 (d, *J* = 7.7 Hz, 1H), 7.13 (d, *J* = 7.1 Hz, 1H), 6.90 (d, *J* = 4.7 Hz, 1H), 6.11 (m, 1H), 4.22 (s, 1H), 3.96 (s, 1H), 3.23 (s, 1H), 2.92 (m, 1H), 2.06 – 1.72 (m, 3H), 1.59 (m, 3H), 1.15 (m, 2H), 0.86 (m, 3H), 0.67 – 0.20 (m, 2H). ; ^{13}C NMR (101 MHz, MeOD) δ 174.78 (s), 163.68 (s), 160.94 (s), 156.91 (s), 147.95 (s), 145.89 (d, *J* = 5.1 Hz), 145.25 (d, *J* = 6.1 Hz), 141.98 (s), 136.76 (s), 129.30 (s), 126.72 (d, *J* = 13.7 Hz), 123.61 (s), 112.51 (d, *J* = 6.1 Hz), 107.93 (s), 104.64 (s), 99.28 (s), 46.97 (s), 31.51 (s), 31.01 (s), 11.92 (d, J = 7.1 Hz), 8.10 (d, *J* = 17.1 Hz), 7.71 (s).; m.p. 158~160 °C; HRMS (ESI) calcd for $C_{28}H_{27}N_6O_4$ $[M+H]^+$: 511.2088, Found 511.2906.
(S)-Cyclopropyl(3-((4-(2-(3,4-dichlorophenyl)-5,6-dihydroxy-1H-benzo[d]imidazol-1-yl) pyrimidin-2-yl)amino)piperidin-1-yl)methanone (***35d,*** as a yellow solid, 58%) ; ^{1}H NMR (400 MHz, DMSO) δ 9.16 (s, 1H), 8.44 (d, *J* = 44.8 Hz, 1H), 7.74 (s, 2H), 7.30 (d, *J* = 39.1 Hz, 1H), 7.08 (s, 1H), 6.57 (m, 1H), 4.26 (s, 1H), 3.91 (s, 1H), 2.99 (s, 1H), 2.84 – 2.53 (m, 1H), 2.08 – 1.86 (m, 1H), 1.66 (m, 2H), 1.51 (m, 2H), 1.23 (m, 3H), 0.88 – 0.65 (m, 3H), 0.57 (m, 1H), 0.25 (m, 1H). ; m.p. 238~239 °C; HRMS (ESI) calcd for $C_{26}H_{25}Cl_2N_6O_3$ [M+H]+ : 539.1360, Found 541.2558.
(S)-Cyclopropyl(3-((4-(2-(4-fluoro-3-(trifluoromethyl)phenyl)-5,6-dihydroxy-1H-benzo[d] imidazol-1-yl)pyrimidin-2-yl)amino)piperidin-1-yl)methanone (***35e,*** as a yellow solid, 60%) ; ^{1}H NMR (400 MHz, MeOD) δ 8.36 (d, *J* = 68.0 Hz, 1H), 7.88 (d, *J* = 4.4 Hz, 1H), 7.75 (s, 1H), 7.48 – 7.37 (m, 1H), 7.13 (d, *J* = 6.5 Hz, 1H), 6.88 (d, *J* = 147.6 Hz, 1H), 6.34 (s, 1H), 4.60 (s, 1H), 4.31 – 3.82 (s, 2H), 3.13 (m, 1H), 2.76 (m, 1H), 2.06 – 1.70 (m, 3H), 1.60 (m, 2H), 1.29 (m, 2H), 1.01 – 0.54 (m, 4H), 0.32 (m, 1H). ; ^{13}C NMR (101 MHz, MeOD) δ 174.76 (s), 163.74 (s), 162.69 (s), 161.68 (s), 158.97 (s), 146.61 (d, *J* = 10.4 Hz), 145.64 (d, *J* = 3.4 Hz), 136.83 (s), 136.39 (s), 129.04 (d, *J* = 14.7 Hz), 118.65 (s), 118.44 (s), 106.24 (s), 104.82 (s), 103.08 (s), 98.83 (s), 50.93 (s), 46.96 (s), 43.93 (s), 31.42 (s), 31.03 (s), 11.85 (s), 7.97 (d, *J* = 2.6 Hz).; m.p. 146~147 °C; HRMS (ESI) calcd for $C_{27}H_{25}F_4N_6O_3$ $[M+H]^+$: 557.1919, Found 557.2937.

3. Evaluation of IC_{50} on JNK3

We used Reaction Biology Corp.'s Kinase HotSpotSM service (www.reactionbiology.com) for IC_{50} determination of all compounds and kinase profiles. Assay protocol: in a final reaction volume of 25 μL, substrate ATF2 5 μM, ATP 10 μM, and JNK3(h) (5-10 mU) were incubated with 25 mM Tris (pH 7.5), 0.02 mM EGTA, 0.66 mg/mL myelin basic protein, 10 mM Mg acetate, and [γ-33P-ATP] (specific activity approx. 500 cpm/pmol, concentration as required). The reaction was initiated by the addition of the Mg-ATP mix. After incubation for 40 minutes at room temperature, the reaction was stopped by the addition of 5 μL of a 3% phosphoric acid solution. Then, 10 μL of the reaction was spotted onto a P30 filtermat and washed three times for 5 minutes in 75 mM phosphoric acid and once in methanol prior to drying and scintillation counting.

4. Selected Kinase Profiling

Base reaction buffer: 20 mM Hepes (pH 7.5), 10 mM $MgCl_2$, 1 mM EGTA, 0.01% Brij35, 0.02 mg/ml BSA, 0.1 mM Na_3VO_4, 2 mM DTT, 1% DMSO, Required cofactors are added individually to each kinase reaction. Procedure Step-by-Step ① Prepare substrate in freshly prepared base reaction buffer ②

Deliver any required cofactors to the substrate solution above ③ Deliver indicated kinase into the substrate solution and gently mix ④ Deliver compounds in 100% DMSO into the kinase reaction mixture by Acoustic technology (Echo550; nanoliter range), incubate for 20 minutes at room temperature ⑤ Deliver 33P-ATP into the reaction mixture to initiate the reaction. ⑥ Incubate kinase reaction for 2 hours at room temperature ⑦ Detect kinase activity by P81 filter-binding method.

5. Cell viability assays

Primary Rat Cortex Neurons, Sprague Dawley (Gibco, A36512) were cultured in Neurobasal™ Plus culture medium (Gibco, A3582901), supplemented with B-27™ Supplement (Gibco, A3582801) and 0.5mM GlutaMAX™ Supplement (Gibco, 35050061) at 37 °C in a humidified 5% CO_2 atmosphere. Plate ~2 × 105 live cells per well in a poly-D-lysine/laminin coated 24-well plate. For neural differentiation, half of the medium was replaced with fresh complete medium every third day. On day 6, remove half the volume of media from the culture plate and add an equal amount of complete culture media containing test compounds or vehicle to each well and incubated for 90 min at 37 °C and 5% CO_2. Immediately prior to use, amyloid β-Protein (1-42) (HFIP-treated) (Bachem, 4090148.0100) were dissolved in 1% NH_4OH, further diluted with culture medium, and added into the plates to a final concentration of 10 μM, and incubated cells for 24 h with vehicle control or test compounds in the presence or absence of $A\beta_{42}$. Cell viability was measured using the MTT [3-(4, 5-dimethylthiazol-2-yl)-2,5-diphenyltetrazolium bromide] assay. MTT solution was added into each well at a final concentration of 0.5mg/ml and cells were incubated at 37 °C for 4 h. The absorbance was detected at 540 nm (reference 650nm) with a Microplate Reader. All results were normalized to OD values measured from the vehicle control (DMSO).

5.1. Effect of selective JNK3 inhibitors on amyloid-β-induced apoptosis signaling activation and amyloid-β-induced JNK activation

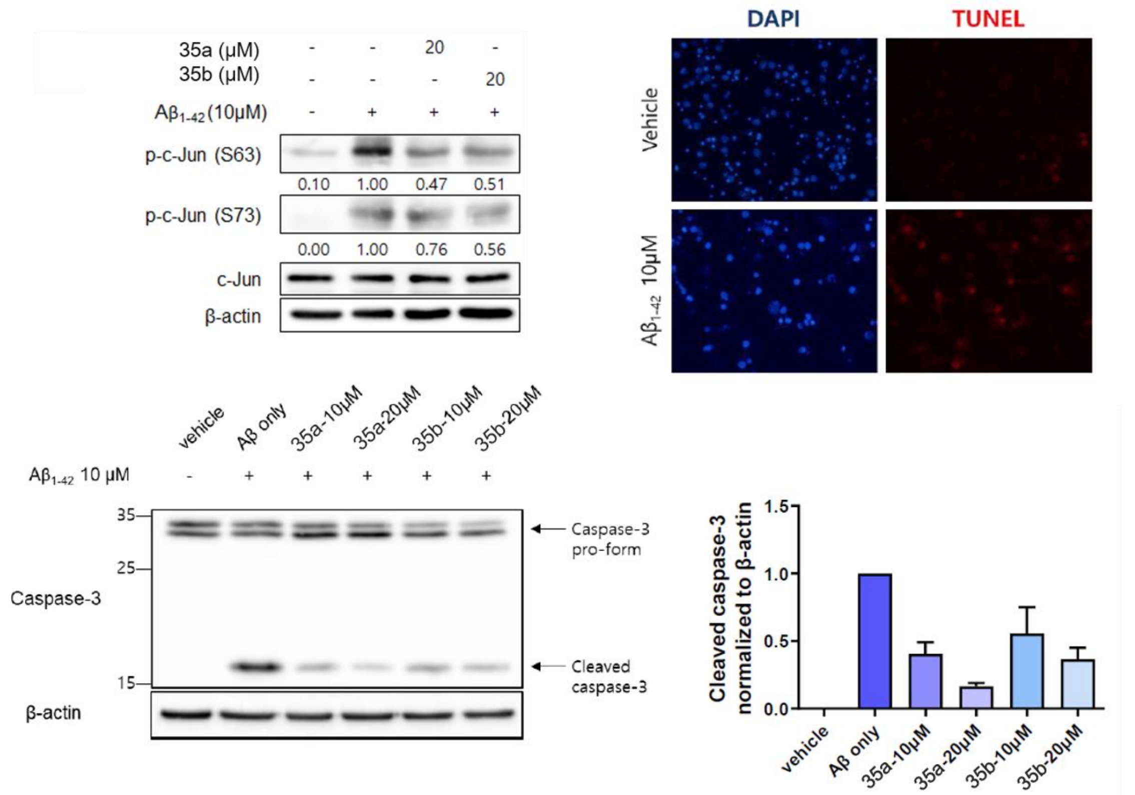

Figure S1. Effect of selective JNK3 inhibitors on amyloid-β-induced apoptosis in primary rat neuron

Amyloid-β is known to mainly cause apoptosis during cell death, so it was confirmed whether the derived selective JNK3 inhibitors **35a** and **35b** affect the activation (cleavage) of caspase-3, which are apoptosis-related signaling substances. On the 5th day of rat primary cortical neuron differentiation, each compound was pre-treated for 90 minutes and then treated with 10 μM amyloid-β1-42 (HFIP-treated) for 24 hours. The signaling activation of caspase-3 was confirmed by western blot. It was confirmed that the cleaved form, an activated form of caspase-3, was increased when amyloid-β was treated in neurons, and compounds **35a** and **35b** inhibited the activation of caspase-3 by amyloid-β treatment (Figure S1). This means that selective JNK3 inhibitors can inhibit apoptosis signaling by amyloid-β in neurons, thereby inhibiting apoptosis. Also it was confirmed whether JNK activation was induced by amyloid-β in neurons, and whether the derived selective JNK3 inhibitors (**35a** and **35b**) could inhibit JNK activation induced by amyloid-β. It was confirmed that compounds **35a** and **35b** inhibit phosphorylation of c-jun induced by amyloid-β in neurons.

5.2 Cytotoxic effect of selective JNK3 inhibitors in neurons without amyloid-β presence

The experiment on cell viability was conducted with selected compounds (001, 150, 151, 166-previous developed JNK3 inhibitors) preceded by a single concentration (20 μM), and it was confirmed that there was no toxicity to the cell viability of primary rat cortical neurons**.**

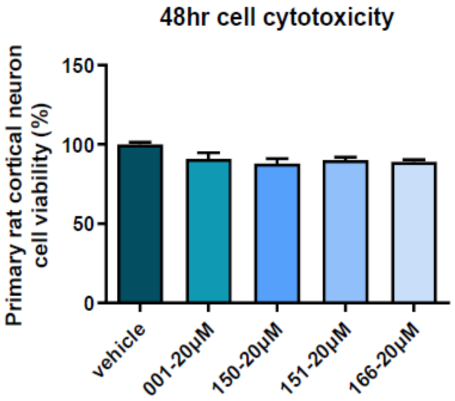

Figure S2. Cytotoxic effect of JNK3 inhibitors in neurons

6. ^{1}H NMR Spectra of Final products

9c

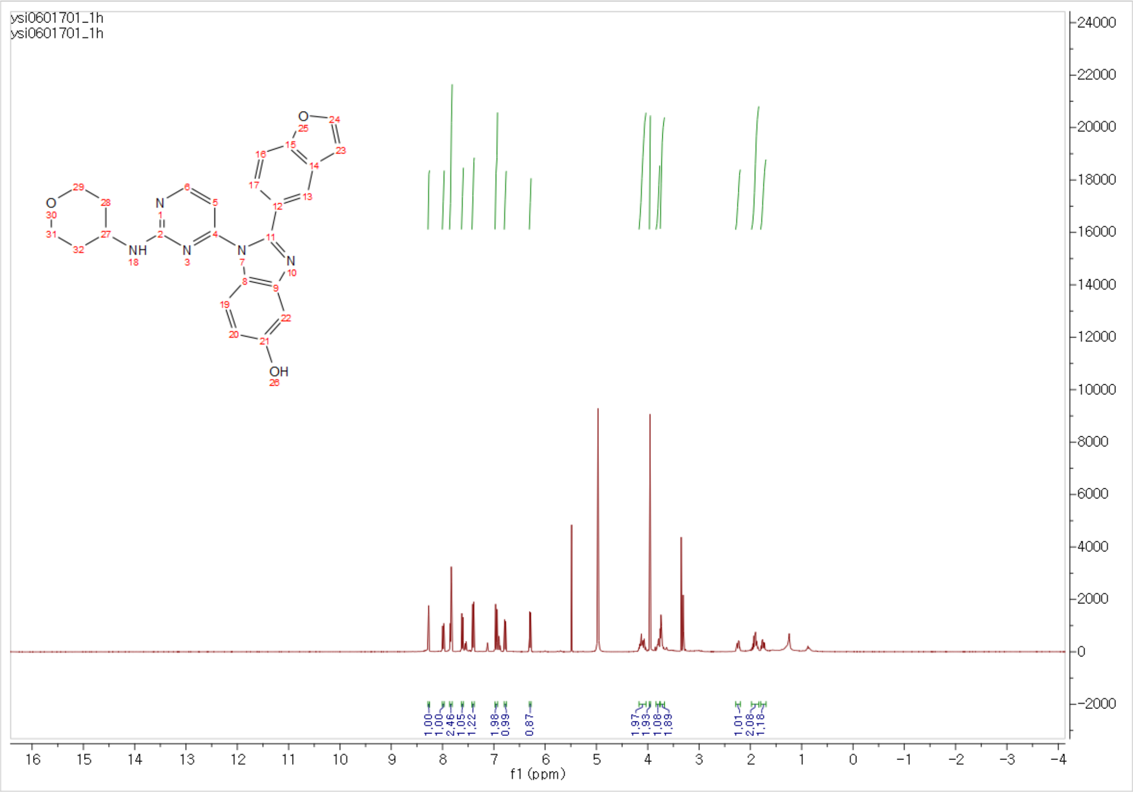

10f

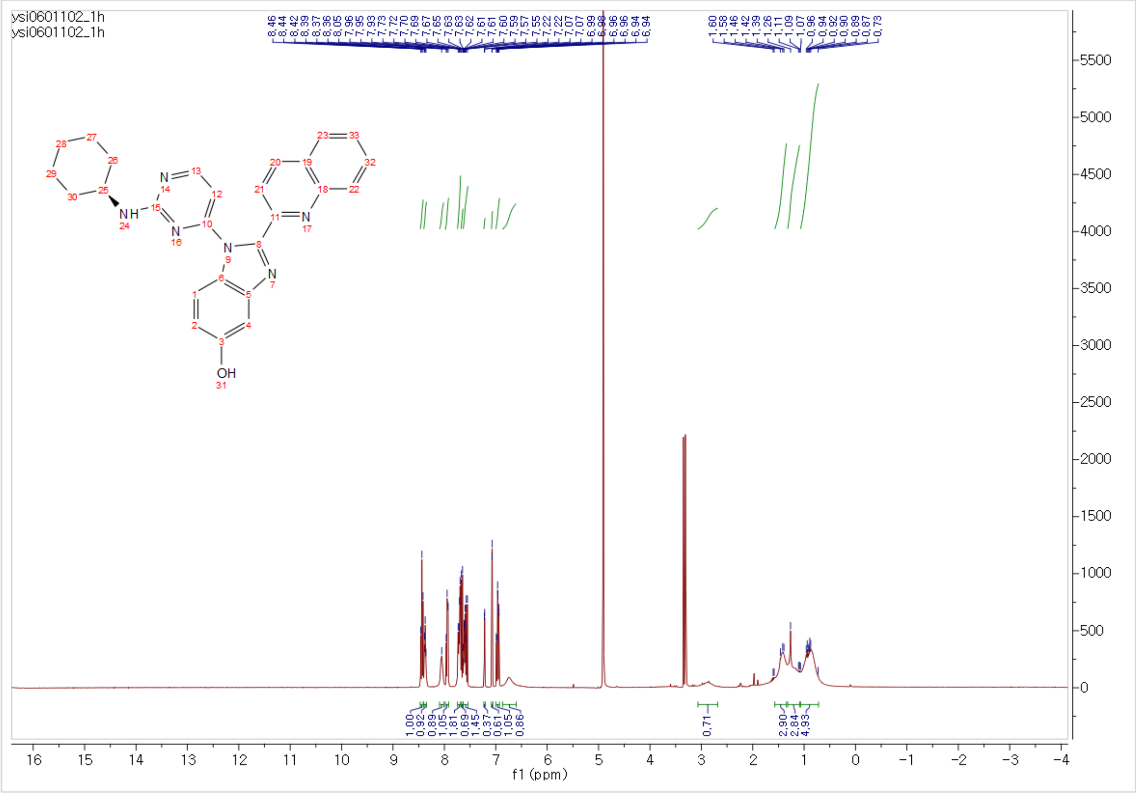

13d

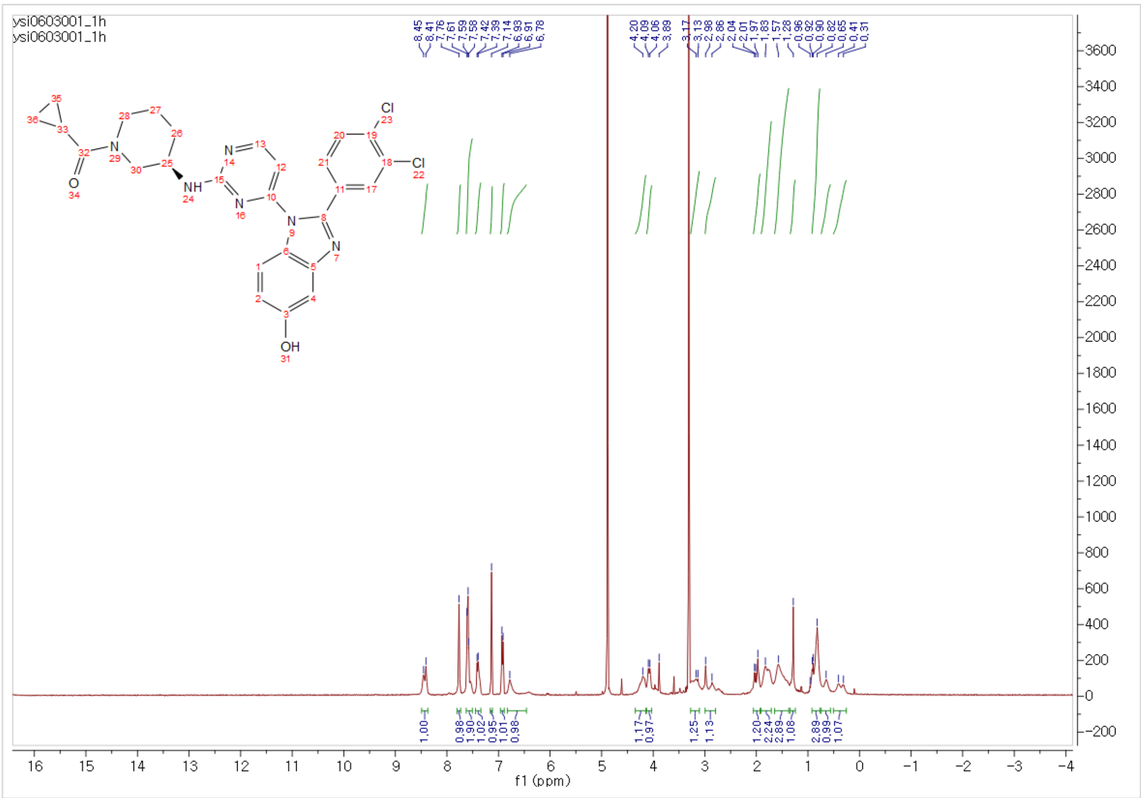

22b

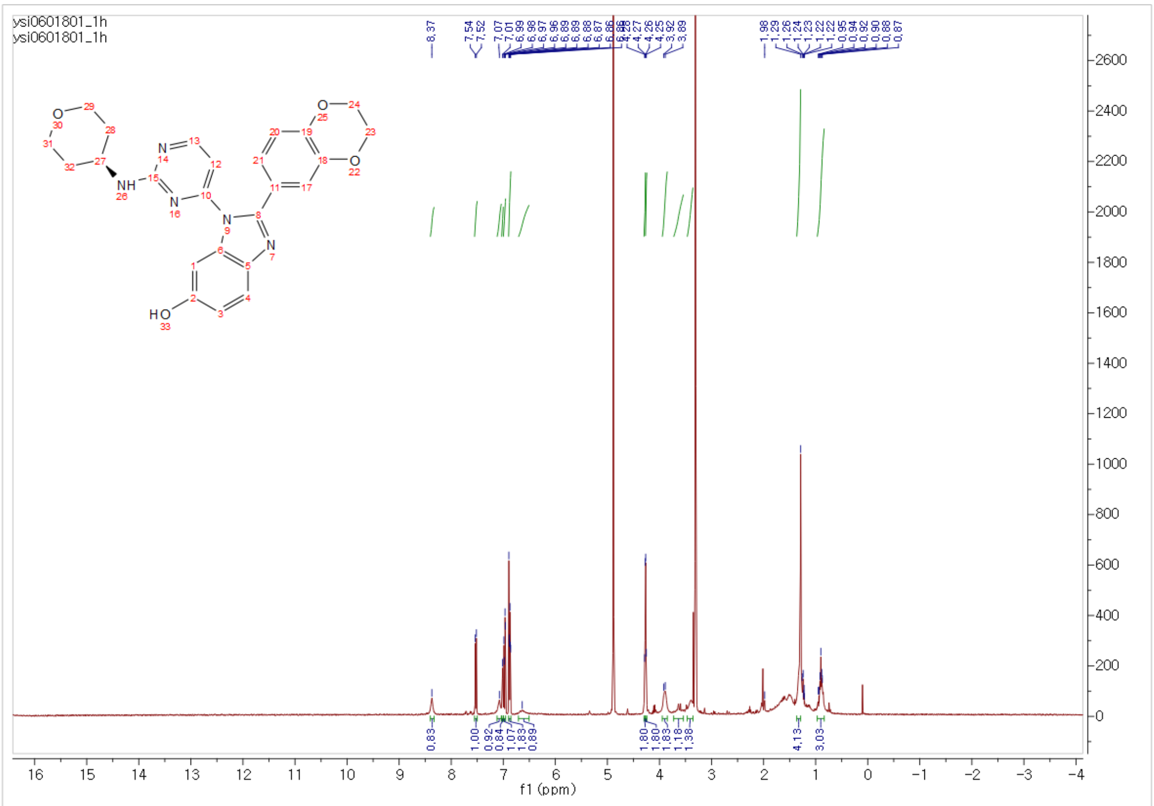

22c

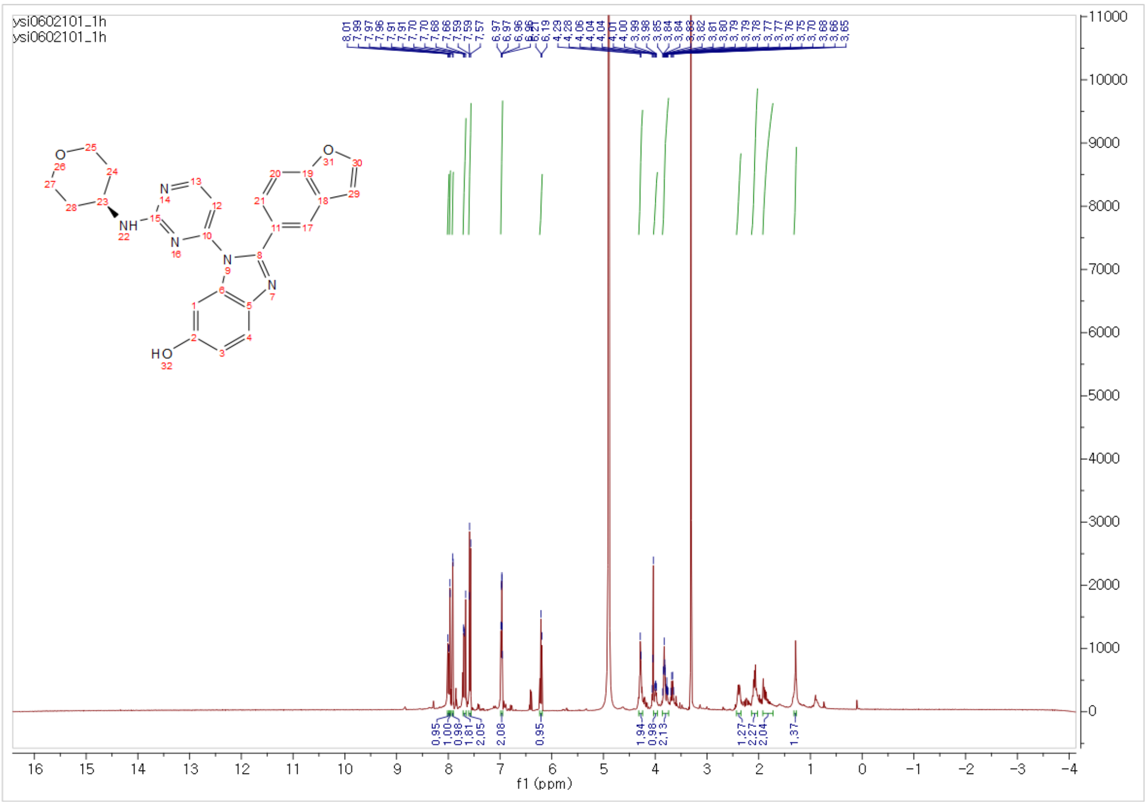
ysi0602101_1h
ysi0602101_1h
f1 (ppm)

35b

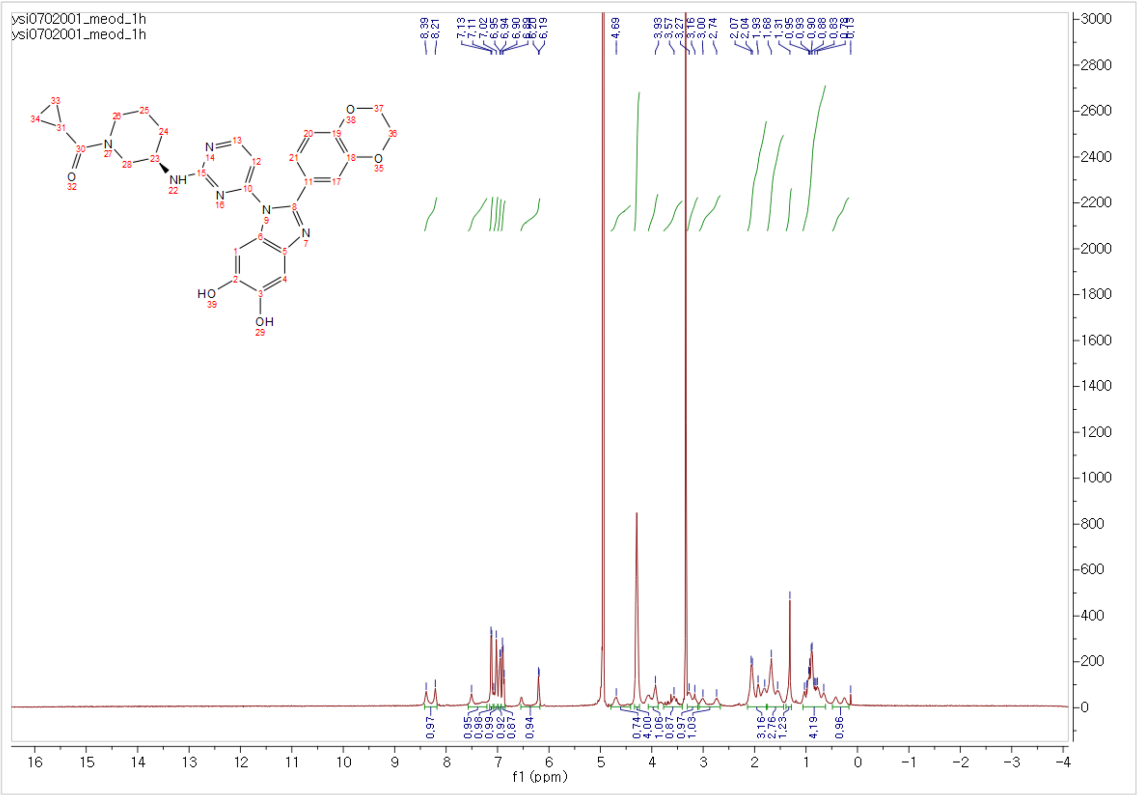
ysi0702001_meod_1h
ysi0702001_meod_1h
f1 (ppm)

35d

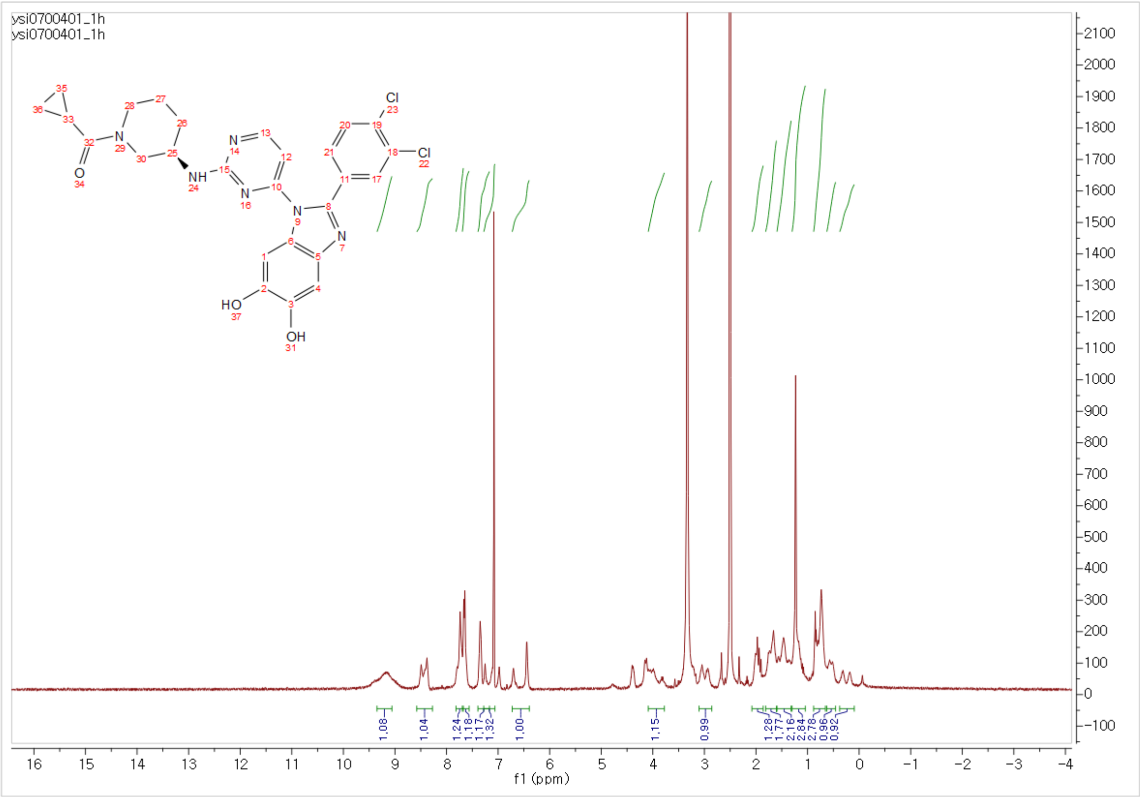
ysi0700401_1h
ysi0700401_1h
f1 (ppm)

35d

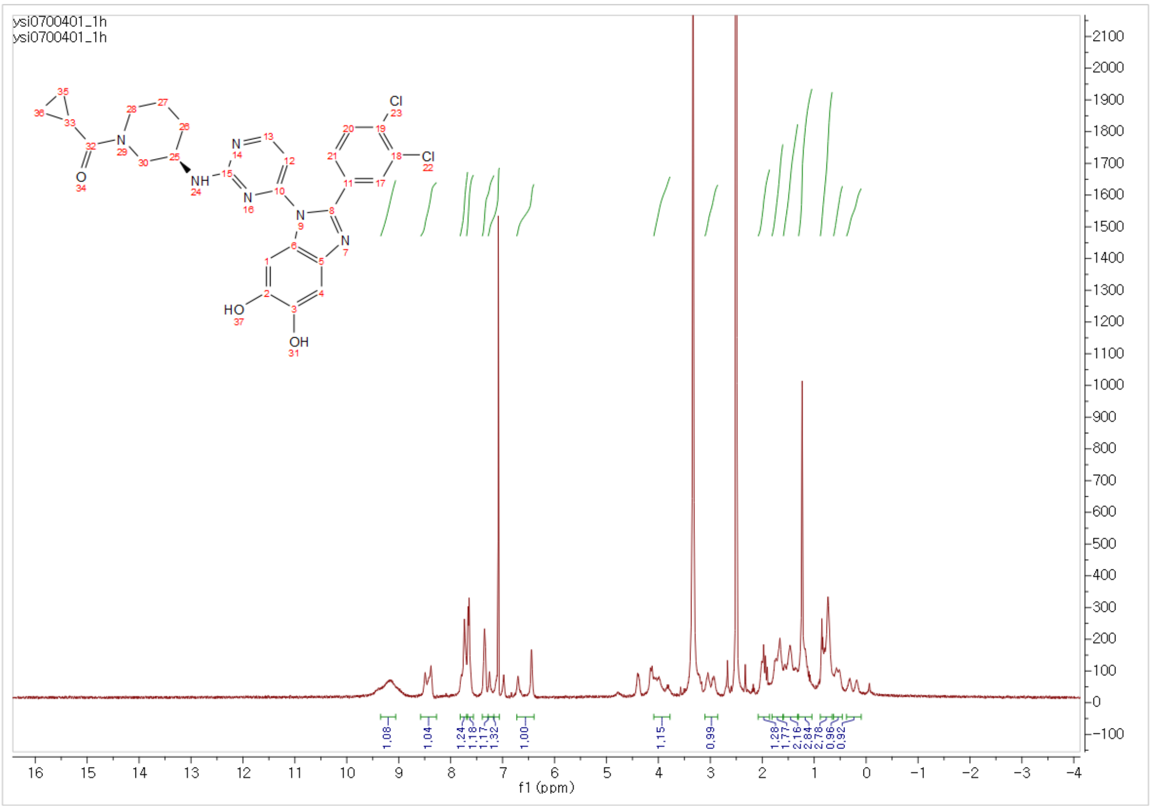
ysi0700401_1h
ysi0700401_1h
f1 (ppm)

35e

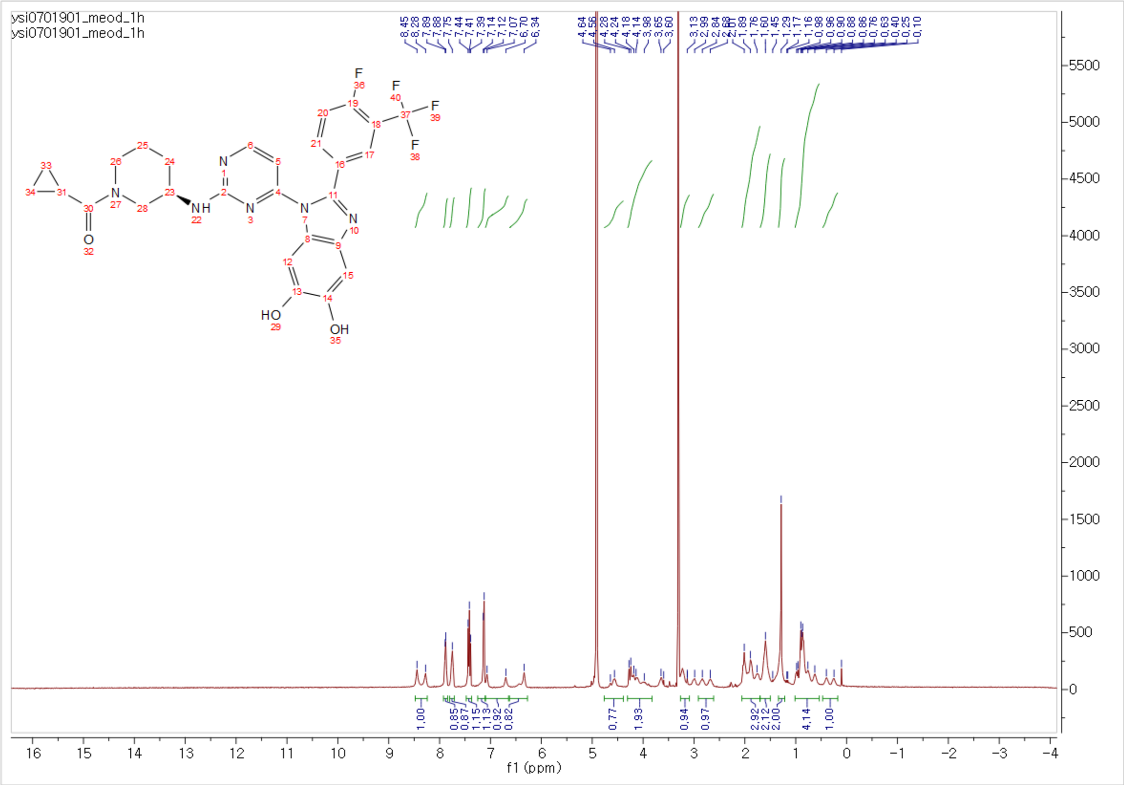

7. ^{13}C NMR Spectra of Final products

9c

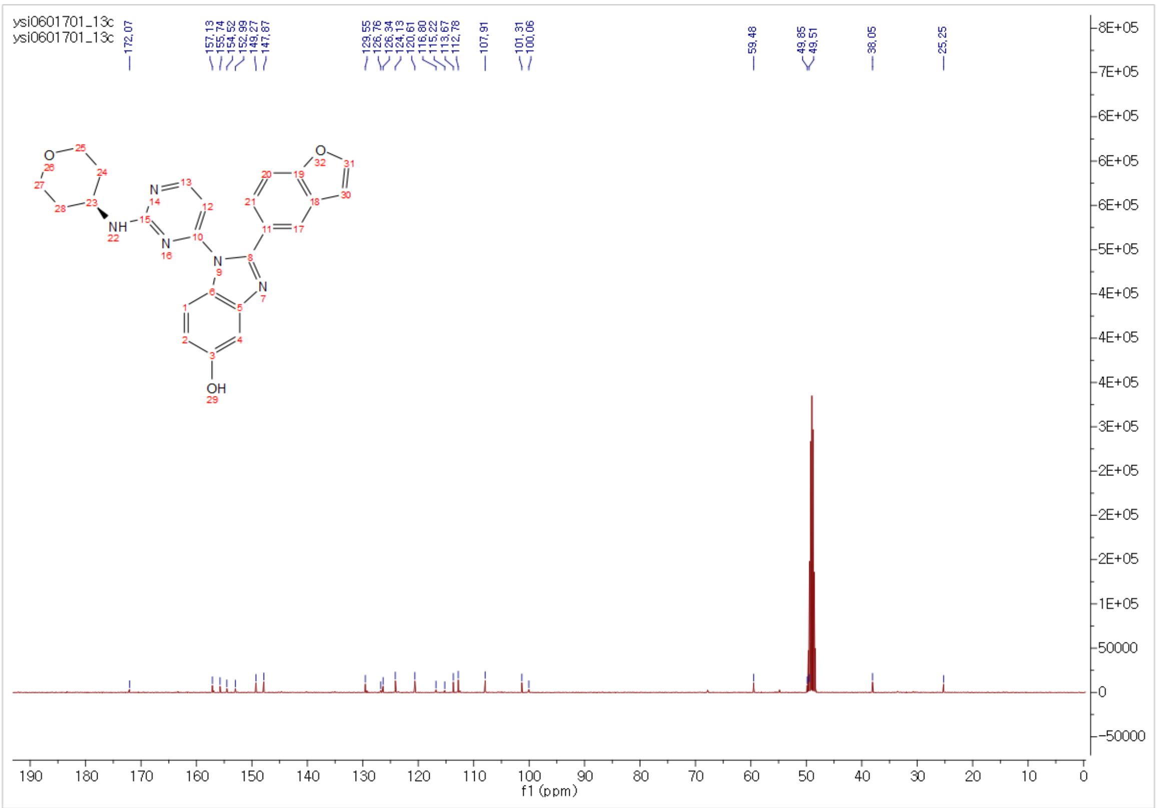

10b

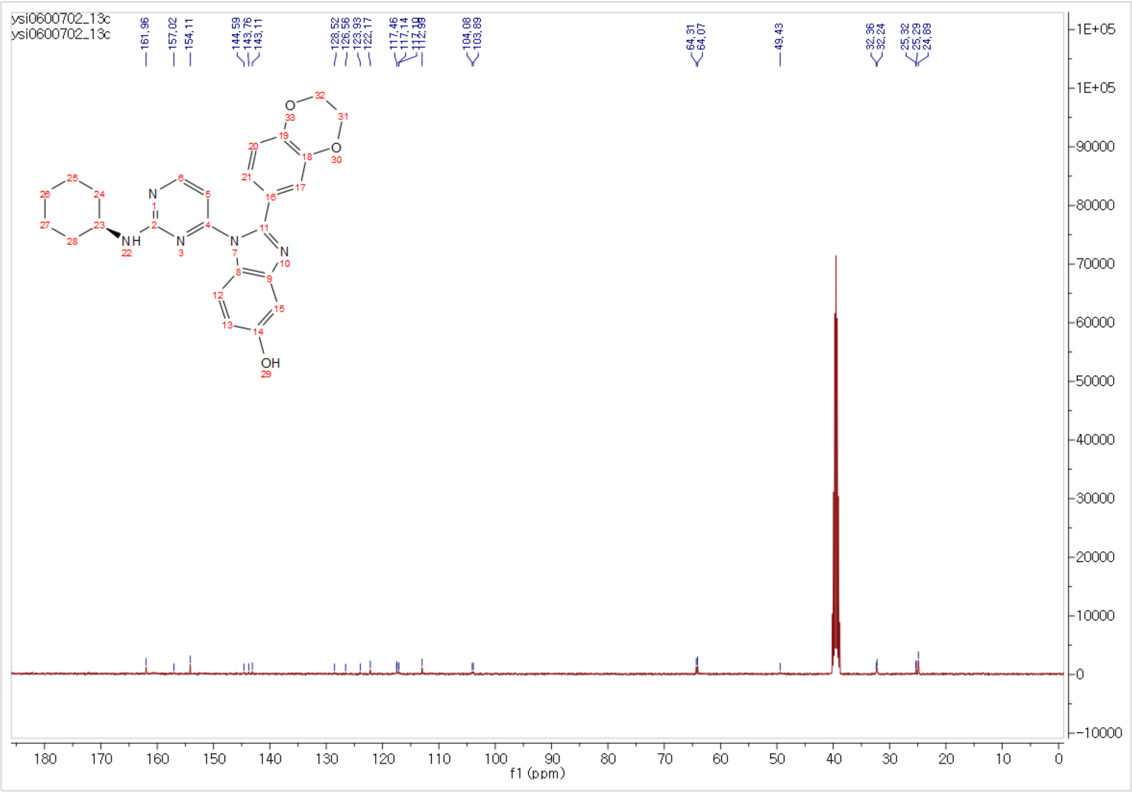
ysi0600702_13c
ysi0600702_13c
161.96
157.02
154.11
144.59
143.76
143.11
128.52
126.56
123.93
122.17
117.46
117.14
104.08
103.89
64.31
64.07
49.43
32.36
32.24
25.32
25.29
24.89
f1 (ppm)

10f

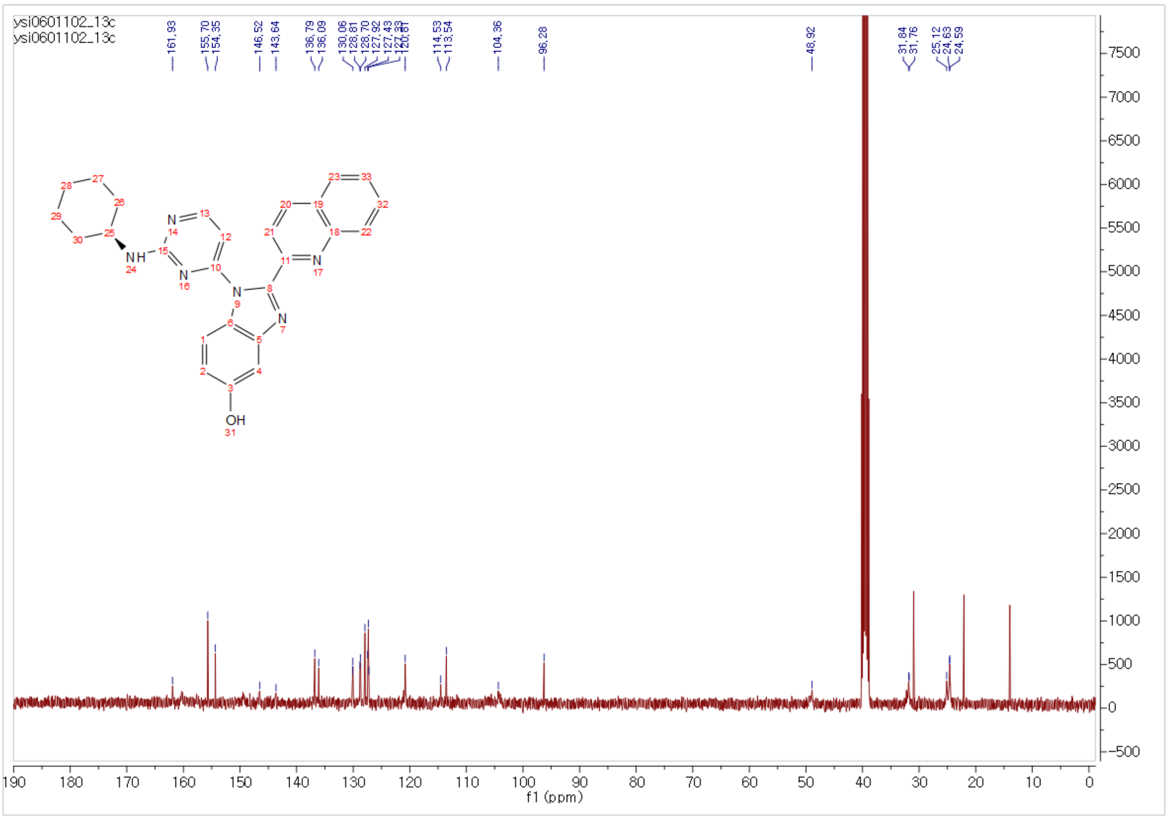
ysi0601102_13c
ysi0601102_13c
161.93
155.70
154.35
146.52
143.64
136.79
136.09
130.06
128.81
128.70
127.92
127.43
120.81
114.53
113.54
104.36
96.28
48.92
31.84
31.76
25.12
24.63
24.59
f1 (ppm)

13f

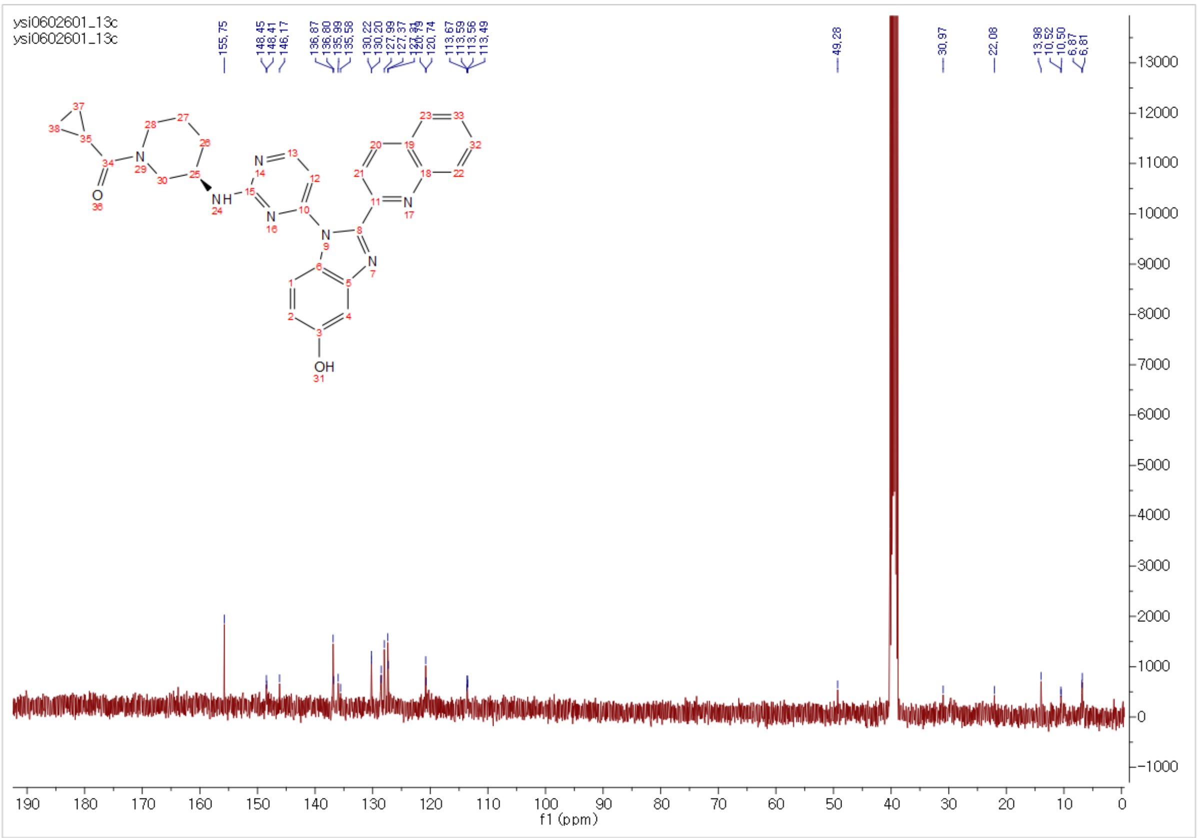

13e

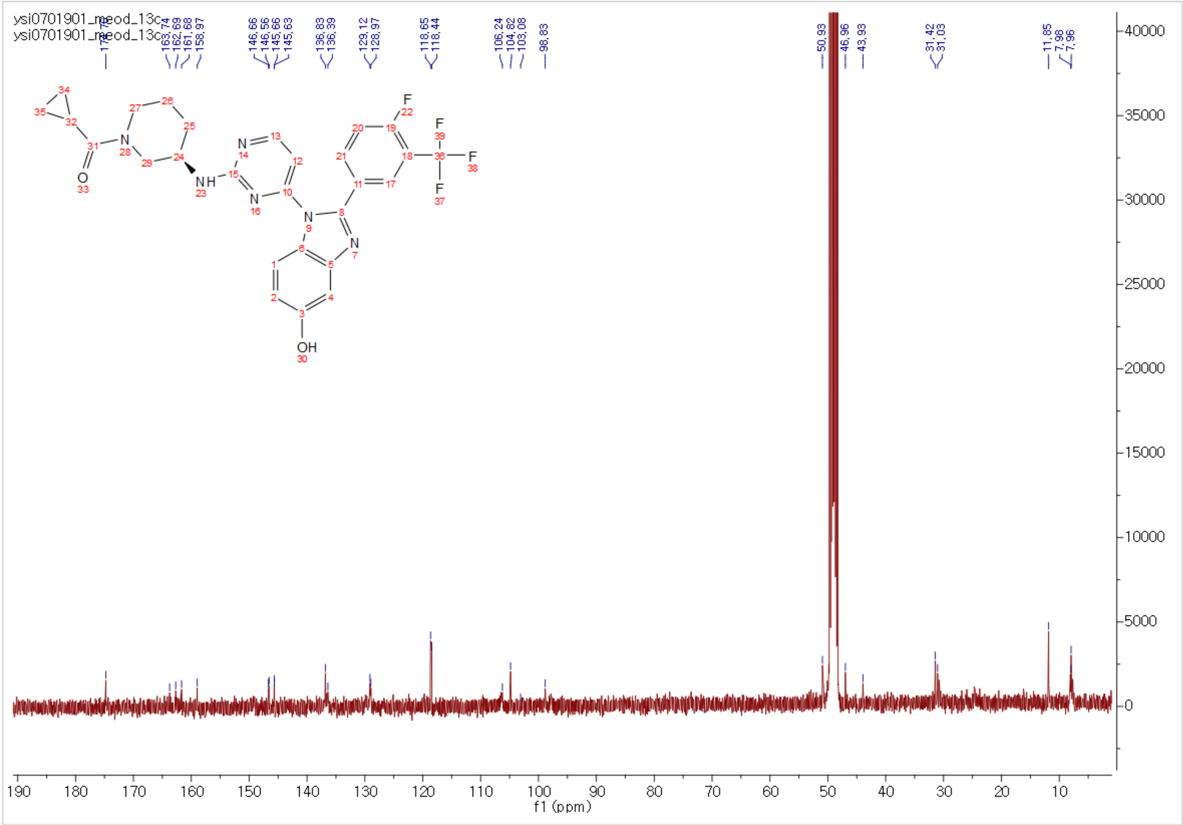

Supplement: Supplementary file 1 [file ijms-22-11084-s001.zip › ijms-1381681-supplementary.pdf]
